# Van Der Waals gap-rich BiOCl atomic layers realizing efficient, pure-water CO_2_-to-CO photocatalysis

**DOI:** 10.1038/s41467-021-26219-6

**Published:** 2021-10-11

**Authors:** Yanbiao Shi, Jie Li, Chengliang Mao, Song Liu, Xiaobing Wang, Xiufan Liu, Shengxi Zhao, Xiao Liu, Yanqiang Huang, Lizhi Zhang

**Affiliations:** 1 Key Laboratory of Pesticide & Chemical Biology of Ministry of Education, Institute of Environmental & Applied Chemistry, College of Chemistry, Central China Normal University, 152 Luoyu Road, 430079 Wuhan, China; 2 State Key Laboratory of Catalysis, Dalian Institute of Chemical Physics, Chinese Academy of Sciences, 116023 Dalian, China

**Keywords:** Photocatalysis, Photocatalysis

## Abstract

Photocatalytic CO_2_ reduction (PCR) is able to convert solar energy into chemicals, fuels, and feedstocks, but limited by the deficiencies of photocatalysts in steering photon-to-electron conversion and activating CO_2_, especially in pure water. Here we report an efficient, pure water CO_2_-to-CO conversion photocatalyzed by sub-3-nm-thick BiOCl nanosheets with van der Waals gaps (VDWGs) on the two-dimensional facets, a graphene-analog motif distinct from the majority of previously reported nanosheets usually bearing VDWGs on the lateral facets. Compared with bulk BiOCl, the VDWGs-rich atomic layers possess a weaker excitonic confinement power to decrease exciton binding energy from 137 to 36 meV, consequently yielding a 50-fold enhancement in the bulk charge separation efficiency. Moreover, the VDWGs facilitate the formation of VDWG-Bi-V_O_^••^-Bi defect, a highly active site to accelerate the CO_2_-to-CO transformation via the synchronous optimization of CO_2_ activation, *COOH splitting, and *CO desorption. The improvements in both exciton-to-electron and CO_2_-to-CO conversions result in a visible light PCR rate of 188.2 μmol g^−1^ h^−1^ in pure water without any co-catalysts, hole scavengers, or organic solvents. These results suggest that increasing VDWG exposure is a way for designing high-performance solar-fuel generation systems.

## Abstract (web summary)

Efficient CO_2_ photoreduction in pure water remains challenging. Here, the authors propose to use van der Waals gaps-rich BiOCl atomic layers with low exciton binding energy and abundant surface oxygen vacancies for CO_2_ to CO conversion.

## Introduction

Interest in the photocatalytic CO_2_ reduction (PCR) process has increased in recent years because of its potential to solve the energy crises and greenhouse effect^1–5^. More specifically, PCR can harvest the intermittent solar light by storing the solar energy into the chemical bonds of value-added chemicals, fuels, and feedstocks^6–12^. Through the use of PCR to convert atmospheric “CO_2_ waste”, the anthropogenic carbon cycle could be potentially closed, thereby rendering our planet more sustainable^13–18^.

However, the industrialization of a large-scale, low-cost, and green PCR process remains an ongoing issue. PCR reactions tend to proceed in media containing KHCO_3_ or organic solvents, such as acetonitrile and dimethylformamide^19^. Although enhancing CO_2_ solubility, availability, and activation, these organic additives increase costs and environmental risks. In the majority of cases, noble metal-based macromolecules including [Ru(bpy)_3_]Cl_2_, Ru(phen)_3_^2+^, and Ir(ppy)_3_ are used as co-catalysts to furnish CO_2_-reducing sites^20,21^. Another obstacle to PCR industrialization lies in the use of hole scavengers such as triethanolamine, ascorbic acid, and ethylenediaminetetraacetic acid^22^. These species quench holes to restrain electron–hole recombination, but are expended in an unrenewable manner. To sustain the PCR, constant feeding of fresh scavengers is thus required.

An appealing alternative is to operate the PCR process using only light, a suitable photocatalyst, and water^23–30^. However, this route typically results in lower performances than its counterparts employing organic media, co-catalysts, and hole scavengers, with PCR rates of < 50 μmol g^−1^ h^−1^. This phenomenon is generally believed to arise from insufficient exciton dissociation and a lack of catalytically active sites^31,32^. Overcoming these limitations to attain a high-performance PCR process in pure water is, therefore, a subject of interest and priority, despite its challenging nature.

Edge facets of two-dimensional layered nanomaterials with rich van der Waals gaps (VDWGs) are catalytically active. Sites on VDWGs include abundant unsaturated coordination and intermediate binding sites^33,34^. Furthermore, VDWGs have great potential in weakening excitonic binding to boost electron and hole separation^35^. Therefore, designing VDWGs-rich low-dimensional nanostructures may provide an efficient route to high-performance pure water PCR, which remains unexplored.

In this study, we tackle the challenges related to the pure water PCR process by employing a photocatalyst composed of ultrathin BiOCl nanosheets where the basic plane bears van der Waals gaps (VDWGs). The VDWG exposure in this system reaches up to 99% across the entire nanosheet, which is achieved by a gas-phase exfoliation process driven by a reaction similar to that used to prepare syngas. The presence of plentiful VDWGs results in more electrons for CO_2_ reduction by weakening the exciton binding strength and, in turn, accelerating electron–hole separation. These surviving electrons further reduce CO_2_ to CO along low-energy trajectories, catalyzed by VDWG-Bi-V_O_^••^-Bi structural motifs. These benefits from the VDWGs, therefore, offered ultrathin BiOCl nanosheets a pure water PCR rate of 188.2 μmol g^−1^ h^−1^, which is comparable to many co-catalyst-, scavenger-, and organic-engaged PCR processes.

## Results and discussion

### Chemical structure characterization

We utilized bismuth oxychloride as a PCR executor because of its adjustable optical absorption, prominent electronic structure, and tunable reactive sites, which have widely been investigated in renewable energy and environmental applications^23–40^. Bismuth oxychloride consists of a layered structure of [Cl–Bi–O_2_–Bi–Cl] monolayers that are stacked along the *c*-axis and interact through van der Waals forces (Supplementary Fig. 1). Such a layered geometry leads to the preferential exposure of (001)-faceted nanosheets with lateral (100), (010), and (110) facets featuring abundant VDWGs. Our group has pioneered in manipulating pH to increase VDWG exposure, although the achieved VDWG-rich BiOCl facet generally exhibits an exposure level of <80%^33^. Our prior works have also demonstrated the incorporation of carbon nanoclusters into the [Bi_2_O_2_] interior—which attenuates Bi–O bonding—and this provides a further opportunity for enriching VDWGs^36,37^. Thus, we sought to integrate the above two strategies with a high-temperature, gas-phase chemical exfoliation route. We initially combined the pH tuning and carbon modification to prepare the carbon-incorporated VDWG-faceted BiOCl (CBOC-VDWGs). The nanosheet thickness was ~38 nm and the VDWG exposure percentage was 88% (Supplementary Fig. 2). Subsequently, we exfoliated CBOC-VDWGs at 360 °C for 6 h under an atmosphere of argon mixed with water vapor (Supplementary Fig. 3). During this process, the lattice-embedded carbon nanoclusters react with the vapor in a manner similar to the preparation of syngas, i.e., C_(s)_ + H_2_O_(g)_ → CO_(g)_ + H_2(g)_ (Supplementary Fig. 4). The released CO and H_2_ bubbles destroyed the Bi-O covalent coordination, resulting in delaminated nanosheets, and increased VDWG exposure. Meanwhile, the generated H_2_ extracts oxygen out from the lattice to induce the in situ formation of oxygen vacancies (V_O_^••^). The resultant product, the VDWG-rich BiOCl atomic layers, was donated as BOC-VDWGs-AL.

To confirm the synthetic mechanism, we utilized identical experimental parameters to exfoliate two carbon-free samples. More specifically, CBOC-VDWGs-O_2_ was prepared by the calcination of CBOC-VDWGs in O_2_ at 450 °C for 8 h, during which all carbon species were burned out, while BiOCl with a VDWG ratio of 76% (BOC-VDWGs-76) was prepared without using any carbon-contained precursors^36^. Aberration-corrected high-angle annular dark-field scanning transmission electron microscopy (HAADF-STEM) confirmed that BOC-VDWGs-76 features periodic VDWGs on its basic plane (Supplementary Fig. 5). Upon exfoliation, no variations in thicknesses were observed for either sample (Supplementary Figs. 6–7), thereby confirming that the exfoliation was initiated by the carbon embedded in CBOC-VDWGs. The unsuccessful exfoliation of CBOC-VDWGs in the absence of water vapor confirmed the mentioned-above carbon-water reaction (Supplementary Fig. 8). Moreover, the CO and H_2_ signals detected by gas analysis mass spectrometry during the exfoliation of CBOC-VDWGs further clarifies that the carbon-driven exfoliation indeed obeys a manner similar to that involved in the synthesis of syngas (Supplementary Fig. 9). It is noteworthy that the application of such a gas-phase exfoliation process to obtain ultrathin nanomaterials with high product yield (gram scale) renders this process of particular interest (Supplementary Fig. 10).

To examine the generalizability of the exfoliation methodology, we initially synthesized carbon-modified Bi_3_O_4_Cl, BiOBr, BiOI, and MoS_2_, which were subsequently thermally delaminated under the above-described argon/water vapor conditions. The exfoliated samples are all ultrathin nanosheets bearing VDWGs on their basic planes (Supplementary Figs. 11–15). These results suggest that our developed exfoliation strategy could be potentially generalized to convert other bulk layered materials into VDWGs-rich graphene-analogs.

**Fig. 1:**
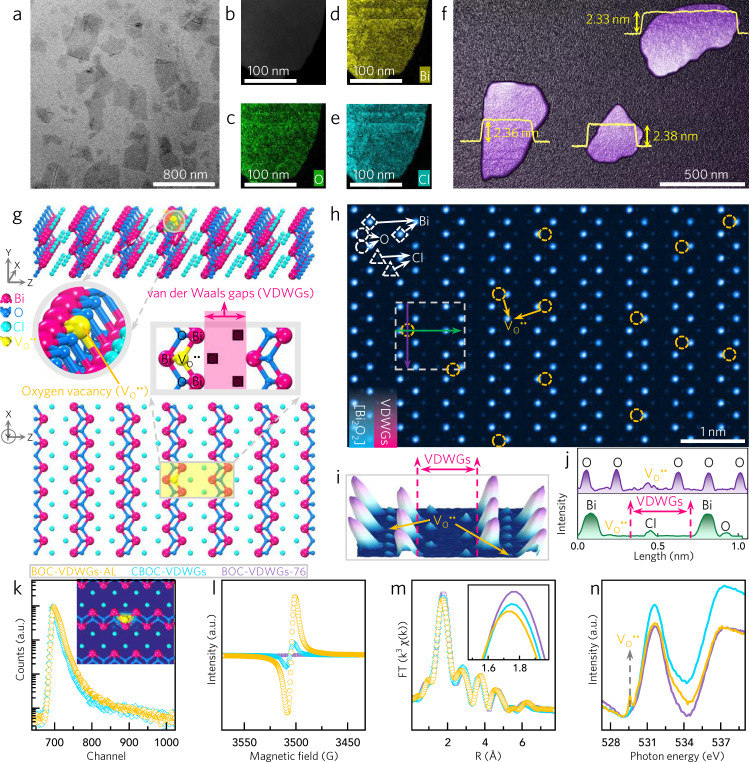
Characterizations of VDWGs-rich structure of BiOCl atomic layers. **a** TEM image, **b** dark-filed STEM image, **c**–**e** elemental mappings, **f** AFM image, **g** theoretical crystalline structures, **h** aberration-corrected HAADF-STEM image, **i** three-dimensional topographic color-coded intensity image (taken from **h**), and **j** intensity profiles (taken along the lines of **h**) of BOC-VDWGs-AL. **k** PAS, **l** EPR, **m** Bi L_3_-edge EXAFS, and **n** O K-edge XANES of BOC-VDWGs-AL, CBOC-VDWGs, and BOC-VDWGs-76. The inset in **k** is the simulation result of the positron density distribution of VDWG-Bi-V_O_^••^-Bi.

We then sought to characterize the as-exfoliated atomically thin BOC-VDWGs-AL featuring VDWGs-rich nanosheet. As suggested by the obtained X-ray diffraction (XRD) pattern, the nanosheets exhibit a BiOCl tetragonal phase (Supplementary Fig. 16). According to some prior studies on the facet engineering of bismuth oxyhalides^36–40^, the lower (002)/(200) peak intensity ratio of BOC-VDWGs-AL (0.54) compared to that of CBOC-VDWGs (0.83) indicates a greater exposure of the VDWG-dominated surfaces in the case of BOC-VDWGs-AL. In addition, transmission electron microscopy (TEM) imaging indicated the presence of an ultrathin nanosheet according to its transparent nature (Fig. 1a). Bi, O, and Cl were found to be uniformly distributed within the nanosheet (Fig. 1b–e), and the selected area electron diffraction (SAED) pattern presented two kinds of spots, corresponding to the (002) and (102) facets (Supplementary Fig. 17). Their intersection angle of 43.4° indicates that the top and bottom of the sheet are capped with the BiOCl (010) facet, which has numerous VDWGs^33,36^. Furthermore, atomic force microscopy (AFM) confirmed the ultrathin nature of the film, with a thickness of ~2.36 nm (Fig. 1f). Given the averaged lateral width estimated as 400–600 nm, we determined the ratio of the (010) facet to the total facets to be 99% (Supplementary Fig. 18), which was calculated based on the previously reported method^40,41^. To the best of our knowledge, such an ultrahigh VDWG coverage percentage is not achieved for other layered materials, such as MoS_2_, C_3_N_4_, black phosphorus, and perovskites.

To directly visualize the VDWGs, we performed atomic-resolution aberration-corrected HAADF-STEM image. The brightest observed in the obtained image spots are Bi (Fig. 1g, h and Supplementary Figs. 19–20) because the signal intensity is proportional to *Z*^2^, where *Z* represents the atomic number; the values of *Z* decrease in the order Bi (83), Cl (17), then O (8)^42,43^. Bi covalently bonds with O to form [Bi_2_O_2_] layers, and so in a single unit cell of BiOCl, two rows of Bi spots that are parallel and neighboring with each other can be observed. Legible gaps, where Cl atoms interact via van der Waals force, were thus mapped between two adjacent [Bi_2_O_2_] layers (Fig. 1i, j). The generation of such a VDWGs configuration is ascribable to the longer bond lengths of Cl–Cl and Bi–Cl compared to that of Bi–O, and also to the ultra-dark Cl signals. The large-area HAADF-STEM image further confirms that the entire nanosheet is dominated by VDWGs.

To identify the VDWG-associated V_O_^••^, we turned to positron annihilation spectroscopy (PAS). The PAS data could be fitted into three positron lifetime components, namely *τ*_1_ (192 ps), *τ*_2_ (396 ps), and *τ*_3_ (2.36 ns) (Fig. 1k and Supplementary Table 1). Previous studies have reported that the values of *τ*_1_, *τ*_2_, and *τ*_3_ originate from the positron annihilations in mono-atom vacancies, cluster-like vacancies, and interfaces or grain boundaries, respectively^44^. Our simulations show that the positrons trapped by Bi-V_O_^••^-Bi on BiOCl VDWGs process a lifetime of 195 ps. As the relative intensity of the experimentally calculated *τ*_1_ is 86.3%, we concluded that BOC-VDWGs-AL is dominated by VDWG-Bi-V_O_^••^-Bi defects. We also found that the VDWGs promoted the emergence of VDWG-Bi-V_O_^••^-Bi, as demonstrated by the significantly lower formation energy of V_O_^••^ close to BiOCl VDWGs than on BiOCl facets free from VDWGs (Supplementary Fig. 21).

The presence of VDWG-Bi-V_O_^••^-Bi was also verified by electron paramagnetic resonance (EPR). More specifically, a peak centered at *g* = 2.004 was observed (Fig. 1l), which has been previously attributed to V_O_^••^^33^. We found that the peak intensity was positively proportional to the VDWG coverage percentage. The trend confirms the promoting role of VDWGs in creating the VDWG-Bi-V_O_^••^-Bi. These findings were also supported by the observation of VDWG-dependent Raman bands, in which the bands at 74 and 102 cm^−1^ were assigned to the first-order vibration modes of *E*_g_ and A_1g_ of Bi metal—proof of the formation of Bi-V_O_^••^-Bi (Supplementary Fig. 22)^36–38^. We further confirmed the presence of VDWG-Bi-V_O_^••^-Bi defects through synchrotron characterizations (Fig. 1m). More specifically, V_O_^••^ results in an increased number of unsaturated Bi atoms, as indicated by the reduced amplitude of the Bi-O peak at 1.77 Å shown in the Bi L_3_-edge extended X-ray absorption fine structure (EXAFS) oscillation curve. Simultaneously, the out-of-plane relaxation of atoms around VDWG-Bi-V_O_^••^-Bi shifts the peak position of BOC-VDWGs-AL to a lower *R* direction compared with that of BOC-VDWGs-76, i.e., by 0.03–0.04 Å. Moreover, O K-edge X-ray absorption near-edge structure (XANES) spectra of BOC-VDWGs-AL revealed an additional peak centered at 529.6 eV (Fig. 1n)^45^, again confirming the presence of VDWG-Bi-V_O_^••^-Bi.

To further confirm the VDWG-Bi-V_O_^••^-Bi defect, we dissected the HAADF-STEM images by extracting the integrated pixel intensity profiles of the lines along the oxygen atomic column (Fig. 1h). For V_O_^••^-free sample, the line intensities did not change obviously (Supplementary Fig. 23). When V_O_^••^ emerged, a significant decline in the line intensity was observed (Fig. 1i, j and Supplementary Fig. 24). The corresponding HAADF-STEM images revealed relatively darker oxygen signals. These results provide a direct and visualized evidence for the formation of VDWG-Bi-V_O_^••^-Bi defects on BOC-VDWGs-AL.

The VDWG-Bi-V_O_^••^-Bi, therefore, imparts BiOCl, which is photoactive only under ultraviolet irradiation, with optical absorption across the whole visible-light region (Supplementary Fig. 25a, b). This is because that VDWG-Bi-V_O_^••^-Bi introduces intraband states between the conduction and valence bands, which narrow the band-gap and hence make the adsorption of long-wavelength visible-light photons feasible^33^. Estimated from ultraviolet photoelectron spectra (UPS), BOC−VDWGs−AL has a valence band maxima of 2.21 eV, thus possessing sufficient potential to photocatalytically oxidize H_2_O into O_2_ (Supplementary Fig. 25c, d).

### PCR activity test

In light of these encouraging observations, we sought to investigate the PCR performance of VDWGs-rich BiOCl. For this purpose, we used a reactor containing only the photocatalyst powder, pure water, and high-purity CO_2_ (Supplementary Fig. 26). The reaction temperature was maintained by continuously incorporating water at 20 °C into the outer jacket of the reactor. Visible light was used as the energy source by employing a 400 nm cutoff filter, and gas chromatography (GC) was employed to identify and quantify the products (Supplementary Figs. 27–29). Consistent with other PCR results obtained using bismuth oxyhalides^29,30^, our designed photocatalyst produces CO as the PCR product.

**Fig. 2:**
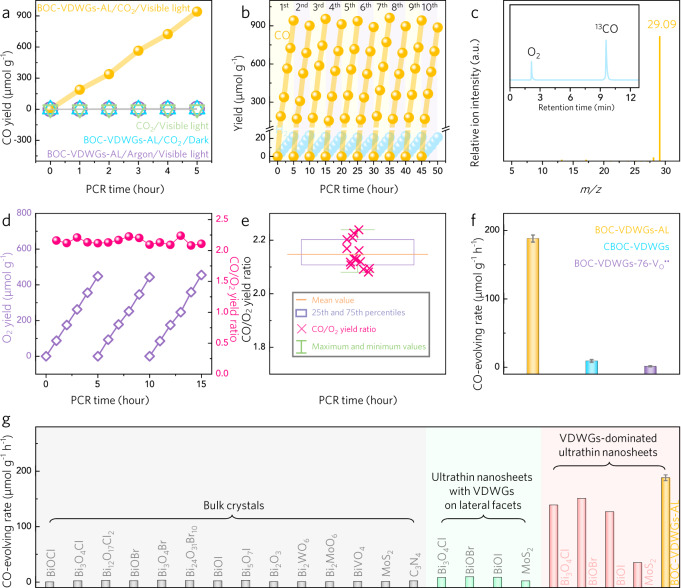
Pure-water PCR performances of VDWGs-rich BiOCl atomic layers. **a** CO yields of four parallel experiments of pure-water PCRs. The first used BOC-VDWGs-AL under visible light in the presence of CO_2_, the second did not involve BOC-VDWGs-AL but still using visible light and CO_2_, the third involved BOC-VDWGs-AL and CO_2_ but under dark, and the fourth employed BOC-VDWGs-AL and visible light but fed with argon. **b** Cycling tests of pure-water PCR performances over BOC-VDWGs-AL for 50 hours. **c** Mass spectra of the product generated from pure-water PCR over visible-light-irradiated BOC-VDWGs-AL fed with ^13^CO_2_. The inset in panel c shows the corresponding GC pattern. **d** Yield of O_2_ detected during pure-water PCR photocatalyzed by BOC-VDWGs-AL, as well as the calculated yield ratio of CO to O_2_. This set of data were obtained from an online PCR system. **e** Statistical result for the CO/O_2_ yield ratio. **f** Comparison of CO-evolving rate of BOC-VDWGs-AL, CBOC-VDWGs, and BOC-VDWGs-76, all under visible light in the presence of pure-water and CO_2_. **g** Comparison of pure-water, CO-evolving rate of BOC-VDWGs-AL and some representative layered photocatalysts. The nanosheet photocatalysts with VDWGs on lateral facets were obtained by conventional exfoliation method, while VDWGs-dominated nanosheets were synthesized by our developed exfoliation strategy. The error bars derived from triplicate experiments.

In the absence of light, CO was not detected (Fig. 2a and Supplementary Fig. 30). CO was also not obtained under visible light when argon or N_2_ was used as the input gas instead of CO_2_. Thus, only when CO_2_, light, and the photocatalyst were introduced into the process did the production of CO via our pure water PCR system proceed (Supplementary Fig. 31). These control experiments, therefore, confirm that CO is produced via this photocatalytic process.

Our results indicated that the visible-light-irradiated BOC-VDWGs-AL catalyzed the transformation of CO_2_-to-CO in pure water at a rate of 188.2 μmol g^−1^ h^−1^, which is a record for bismuth oxyhalide photocatalysts (Supplementary Table 2). The quantum yield reached 2.5% at 400 nm (Supplementary Fig. 32), which is noteworthy in the absence of a co-catalyst, hole scavenger, and organic solvent. Indeed, our result is superior to the rates of CO evolution reported in other pure water PCR systems, and is comparable to those with using co-catalysts, scavengers, and organic solvents (Supplementary Table 3). Under the irradiation of AM1.5 G simulated sunlight, a CO production rate of up to 152.92 μmol g^−1^ h^−1^ was attained (Supplementary Fig. 33 and Supplementary Table 4).

Consecutively operating the pure water PCR for 5 h (one cycle) yielded 900 μmol g^−1^ of CO (Fig. 2b; Supplementary Table 5). The CO_2_-to-CO selectivity exceeded 97% with a trace of H_2_ as the byproduct, which was usually less than 5 μmol g^−1^ h^−1^ in pure water. Such high selectivity could be attributed to the lower barrier for proton transfer to CO_2_ than proton dimerization to H_2_ (Supplementary Fig. 34). NMR spectroscopy and GC–MS unraveled that no gaseous or liquid hydrocarbons were detected (Supplementary Figs. 35 and 36). After each cycling test, the reaction cell was vacuumized and the water was renewed, while the catalyst was not subject to any treatments. Under these conditions, the obtained photoactivity was maintained over 10 cycles, with the rate varying only within 5% (Supplementary Fig. 37). Moreover, the carbon mass balance for the BOC-VDWGs-AL was almost 100% (Supplementary Fig. 38). After 20 h of reaction, we observed no appreciable impairment in the structural motif, exposure percentage, or V_O_^••^ density of the VDWGs (Supplementary Fig. 39). These observations demonstrate the structural and componential sturdiness of our VDWGs-rich photocatalyst. More importantly, the turnover number (TON) of CO product for the BOC-VDWGs-AL was up to about 26.7 (Supplementary Fig. 40).

Subsequently, ^13^C isotope labeling experiments were carried out to confirm the source of the detected CO (Supplementary Fig. 41). More specifically, a *m*/*z* peak at 29.09, corresponding to ^13^CO, was observed as the main signal throughout 12 h of PCR reaction using ^13^CO_2_ as the source gas (Fig. 2c and Supplementary Fig. 42)^46–53^, confirming that the detected CO was derived from the input CO_2_ gas.

The prerequisite to initiate the PCR in pure water rests in the ability of our BOC-VDWGs-AL to photocatalyze water oxidation. During this step, holes photogenerated from the valence band oxidize water into dioxygen and protons via 4 h^+^ + 2H_2_O → O_2_ + 4H^+^. Subsequently, electrons from the conduction band, cooperating with the protons, reduce CO_2_ to CO via CO_2_ + 2H^+^ + 2*e*^−^ → CO + H_2_O. In our system, the observed CO and O_2_ evolutions gave a rate ratio of 2.14:1 (Fig. 2d, e), close to the ideal stoichiometric ratio. This result can be attributed to the good airtightness of our reaction apparatus and the occurrence of the pure water PCR reaction.

Using the same reactor and experimental condition, the CBOC-VDWGs gave a PCR rate of 9.3 μmol g^−1^ h^−1^, which was approximately 1/20 that of the BOC-VDWGs-AL (Fig. 2f). For further comparison, we prepared BOC-VDWGs-76-V_O_^••^ by the calcination of BOC-VDWGs-76 under H_2_ at 400 °C for 5 h, and found a VDWG-dependent PCR behavior: VDWGs-76-V_O_^••^ (76% VDWGs) < CBOC-VDWGs (88% VDWGs) < BOC-VDWGs-AL (99% VDWGs). The dependence of the PCR reaction on VDWGs was maintained even after normalization of their rates with the corresponding Brunauer–Emmett–Teller surface areas (Supplementary Fig. 43), indicating that the exfoliation-mediated surface area improvement did not play a dominant role in determining the PCR performance. To extend the breadth of the performance comparison, we prepared additional layered photocatalysts (Supplementary Fig. 44), which possessed PCR rates of <5 μmol g^−1^ h^−1^ (Fig. 2g). Further using the conventional exfoliation method to exfoliate some layered photocatalysts into atomically thin nanosheets with VDWGs on their lateral facets was found to enlarge the surface area to boost PCR (Supplementary Figs. 45 and 46), but the rates remained at least 19.8-fold lower than that of BOC-VDWGs-AL. Following exfoliation using our developed strategy, the nanosheets were dominated by VDWGs and remained atomically thin, but their performances were comparable to that of BOC-VDWGs-AL, thereby highlighting the importance of VDWGs in the PCR. No carbon dopants or species were detected in BOC-VDWGs-AL (Fig. 1b, e and Supplementary Figs. 4, 47), ruling out possible doping effects. Taken together, the above results allow us to designate the VDWGs as the performance descriptor.

### Characterization of electron–hole separation efficiency

We subsequently sought to clarify how the VDWGs endow BiOCl with superior pure water PCR ability. In this context, Xie and co-workers reported that deeper VDWGs could restrict exciton, and thereby strengthen the electron–hole attractions in BiOBr^54–56^. As our case (with a VDWG depth of < 3 nm) is the opposite, we therefore postulated that our high-percentage VDWGs may exhibit a lower confinement power and consequently reduce the excitonic effect. Thus, we further envisioned that one contributor to the performance was the VDWGs-promoted electron–hole separation.

**Fig. 3:**
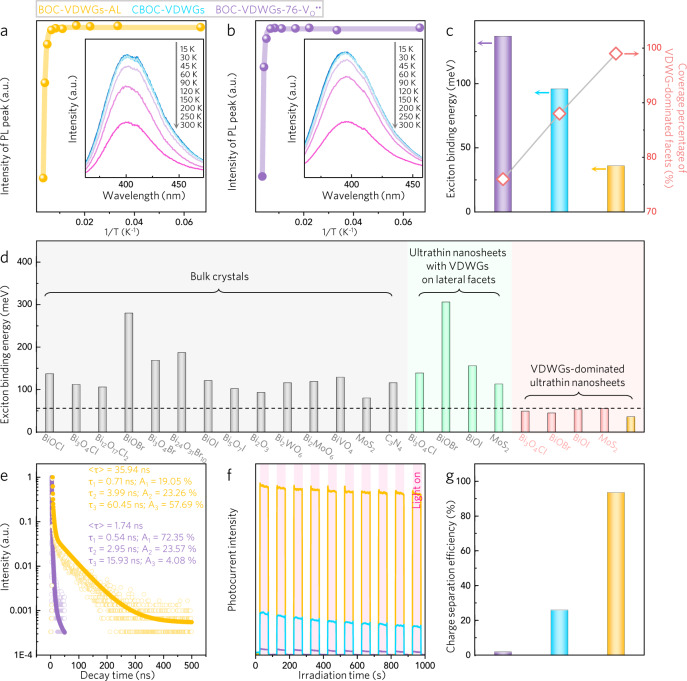
Promoting effect of VDWGs on exciton binding energy and, consequently, on electron–hole separation. **a**, **b** PL of BOC-VDWGs-AL (**a**) and BOC-VDWGs-76 (**b**) as a function of operation temperature. **c** Comparisons of *E*_b_ and VDWG coverage percentage of BOC-VDWGs-AL, CBOC-VDWGs, and BOC-VDWGs-76. **d** Comparisons of *E*_b_ of BOC-VDWGs-AL and some representative layered photocatalysts. **e** Time-resolved PL of BOC-VDWGs-AL and BOC-VDWGs-76. **f**, **g** Transient photocurrent responses (**f**) and bulk charge separation efficiencies (**g**) of BOC-VDWGs-AL, CBOC-VDWGs, and BOC-VDWGs-76.

To test this hypothesis, we experimentally probed the exciton binding energy (*E*_b_) following the method reported by the Wang and Zeng groups^57,58^. Thus, we measured the steady-state photoluminescence (PL) spectra under temperatures ranging from 15 to 300 K (Fig. 3a, b). We plotted the PL peak intensity as a function of the reciprocal temperature and exponentially deconvoluted the obtained curve. The value of *E*_b_ was extracted from the following equation:

$$I(T)=\frac{{I}_{0}}{1+{{A}}\,\exp \left(\frac{-{E}_{{{{{{\mathrm{b}}}}}}}}{{k}_{{{{{{\mathrm{B}}}}}}}T}\right)}$$

Where *I*_0_ is the PL intensity at 0 K, *T* is the temperature, and *k*_B_ is the Boltzmann constant. Thus, the *E*_b_ for BOC-VDWGs-76 was determined to be 137 meV (Fig. 3c), which corresponded with the reported theoretical value^43^. A greater exposure of VDWGs results in a lower *E*_b_. Increasing the VDWG proportion from 76 to 99% decreases the *E*_b_ 3.8-fold to 36 meV, which is significantly lower than those of other layered photocatalysts (i.e., 80–280 meV) (Fig. 3d). This VDWGs-mediated ultralow *E*_b_ can be attributed to a number of factors. More specifically, the VDWGs offer channels along which electrons and holes can diffuse and then separate. In addition, the VDWGs-dominated surfaces possess abundant unsaturated atoms or architectures that can efficiently trap electrons or holes to inhibit their recombination. In contrast, the conventionally exfoliated nanosheets, although being atomically thin, exhibit higher *E*_b_ values (i.e., 113–306 meV) since they contain few VDWGs (Supplementary Fig. 48). Complete quenching of the V_O_^••^ in BOC-VDWGs-AL causes negligible fluctuation in the *E*_b_ (Supplementary Fig. 49), demonstrating the considerable capacity of the VDWGs—rather than V_O_^••^—to weaken the exciton binding. The VDWGs-induced reduction in *E*_b_ endows BOC-VDWGs-AL with enhanced electron–hole separation, as validated by the smaller arc radius in electrochemical impedance spectroscopy (Supplementary Fig. 50), the 20.7-fold longer charge carrier lifetime extracted from the time-resolved PL, and by the 11.5-fold higher photocurrent calculated from the transient photocurrent responses compared to those obtained for BOC-VDWGs-76 (Fig. 3e, f)^59,60^. We then evaluated bulk charge separation efficiencies (η_bulk_) of BOC-VDWGs-76, CBOC-VDWGs, and BOC-VDWGs-AL exploiting our reported methodology^36,39^. Their η_bulk_ values were found to be VDWGs-dependent (Fig. 3g). On account of the significantly smaller *E*_b_ value, BOC-VDWGs-AL exhibits a 50-fold higher η_bulk_ than BOC-VDWGs-76.

For more comparison, we prepared BiOCl with other morphologies, including microsphere, microparticle, and nanowire (Supplementary Fig. 51). The effect of these morphologies on the *E*_b_ and η_bulk_ is weak. Only via tailoring the VDWG exposure could the two values be improved simultaneously.

### PCR mechanism

Leveraging V_O_^••^ as a PCR reactive site has been demonstrated on the (001) facet of layered Bi-based photocatalysts^18,26–30,40,43^, while the defect effects of VDWGs remain unexplored. Reported VDWGs’ assets include abundant unsaturated coordination and intermediate binding sites^36,61^. For these reasons, we posited that VDWG-Bi-V_O_^••^-Bi driven catalysis might be the second performance contributor.

**Fig. 4:**
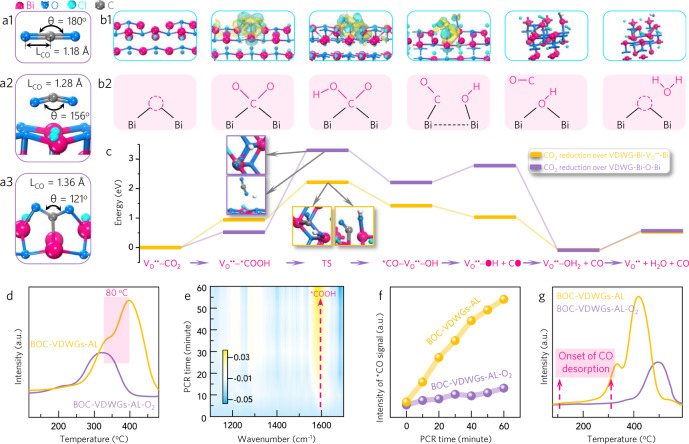
Promoting effect of VDWGs-associated defect on CO_2_-to-CO catalysis. **a** C–O bond length and O–C–O bond angle of free CO_2_ (**a1**), CO_2_ adsorbed on VDWG-Bi-O-Bi (**a2**), and CO_2_ adsorbed on VDWG-Bi-V_O_^••^-Bi (**a3**). **b1** Optimized geometric structures (parts containing charge density differences) and **b2** schematic illustration of intermediates generated during CO_2_-to-CO catalysis over VDWG-Bi-V_O_^••^-Bi. **c** Gibbs free energy diagrams of intermediates generated during CO_2_-to-CO catalysis over VDWG-Bi-O-Bi and VDWG-Bi-V_O_^••^-Bi. **d** CO_2_-TPD of BOC-VDWGs-AL and BOC-VDWGs-AL-O_2_. **e** In situ FTIR of intermediates generated during pure-water PCR over BOC-VDWGs-AL. **f** Comparison of FTIR signal intensity of *CO intermediates from BOC-VDWGs-AL and BOC-VDWGs-AL-O_2_-driven pure-water PCR. **g** CO-TPD of BOC-VDWGs-AL and BOC-VDWGs-AL-O_2_. The VDWG-Bi-O-Bi was constructed by the calcination of VDWG-Bi-V_O_^••^-Bi (BOC-VDWGs-AL) under O_2_ atmonsphere.

To support this assumption, we shifted our attention to computational simulations. In contrast to free CO_2_ with a C–O bond length of 1.18 Å and a O–C–O bond angle of 180°, CO_2_ adsorbed on VDWG-Bi-O-Bi is chemically activated with the two parameters changed to 1.28 Å and 156° (Fig. 4a). The presence of V_O_^••^ further enhances the CO_2_ activation by increasing the bond length to 1.36 Å and reducing the bending angle to 121°. Charge density difference mapping confirms this activation by allowing the observation of substantial electron transfer from VDWG-Bi-V_O_^••^-Bi to CO_2_ along a tridentate trinuclear coordination (Fig. 4b). Reaction pathway simulations reveal that the conversion of CO_2_ to CO over VDWG-Bi-V_O_^••^-Bi and VDWG-Bi-O-Bi takes place via a pathway of *CO_2_ → *COOH → *CO → CO (Supplementary Fig. 52)^62^. In both cases, the transition states (TSs) linking *COOH to *CO + *OH, namely the splitting of *COOH, show the highest energy barriers (Fig. 4c). The barrier is 1.51 eV lower for VDWG-Bi-V_O_^••^-Bi (1.27 eV) than for VDWG-Bi-O-Bi (2.78 eV), indicating that V_O_^••^ promotes *COOH cleavage. In addition, V_O_^••^ is also found to facilitate direct *CO desorption (−0.39 eV) rather than hydrogenation to form *HCO species (+0.25 eV)^63^. Furthermore, the *CO → CO step is exothermal (−0.39 eV) for VDWG-Bi-V_O_^••^-Bi but endothermal (0.57 eV) for VDWG-Bi-O-Bi. Moreover, VDWG-Bi-V_O_^••^-Bi delivers a 0.79 eV higher *CO adsorption energy compared to VDWG-Bi-O-Bi. To provide experimental validation, we carried out CO_2_ and CO temperature-programmed desorption (CO_2_-TPD and CO-TPD) experiments and in situ Fourier-transform infrared (FTIR) spectroscopy. The stronger CO_2_ activation over VDWG-Bi-V_O_^••^-Bi compared to over VDWG-Bi-O-Bi was further confirmed by their CO_2_-TPD signals, which gave peaks at 383 and 303 °C (Fig. 4d), respectively. We also observed increasing trends in the intensities of the FTIR peaks of the *COOH group (1196 and 1541 cm^−1^) and the CO molecule (2017 cm^−1^), in addition to a decreasing trend in the FTIR intensity of CO_2_ (2280–2340 cm^−1^) (Fig. 4e and Supplementary Figs. 53–55), thereby supporting the above-simulated reaction pathways. Moreover, it was found that *CO evolved at a faster rate over VDWG-Bi-V_O_^••^-Bi than over VDWG-Bi-O-Bi (Fig. 4f), thereby experimentally indicating the stronger ability of V_O_^••^ to split *COOH into *CO. Experimental evidence for the simulated V_O_^••^-promoted *CO desorption came from CO-TPD, which revealed that CO desorption began at a considerably lower temperature for VDWG-Bi-V_O_^••^-Bi than for VDWG-Bi-O-Bi (Fig. 4g).

Interestingly, the conventionally exfoliated BiOCl, Bi_3_O_4_Cl, BiOBr, BiOI, and MoS_2_ possessed similar nano-laminar morphologies but significantly lower performances than their VDWGs-rich counterparts exfoliated using our developed method. The key differences between these two kinds of nanosheets are the density and location of their VDWGs. This further indicates that the VDWGs, rather than the ultrathin geometry, are the performance descriptor. We could therefore conclude that an increased exposure of VDWGs results in a lower *E*_*b*_ and in a greater number of VDWG-Bi-V_O_^••^-Bi sites, which enhance electron–hole separation and CO_2_-reducing catalysis to boost the PCR rate.

We also found that quenching the V_O_^••^ could largely decrease the PCR rate because the V_O_^••^ extinction deteriorated the absorption of visible light (Supplementary Fig. 56). To exclude the possibility that VDWG-Bi-V_O_^••^-Bi serves as the exclusive performance contributor, we conducted the following performance comparisons: BOC-VDWGs-AL versus BOC-VDWGs-AL-O_2_ (calcining BOC-VDWGs-AL under O_2_) under ultraviolet irradiation; BOC-VDWGs-AL versus BOC-VDWGs-76-V_O_^••^ under visible light irradiation; and Bi_3_O_4_Cl-VDWGs-AL (VDWGs-dominated Bi_3_O_4_Cl atomic layers) versus Bi_3_O_4_Cl-VDWGs-AL-O_2_ (calcining Bi_3_O_4_Cl-VDWGs-AL under O_2_) under 420 nm monochromatic light irradiation. Relative to BOC-VDWGs-AL, BOC-VDWGs-AL-O_2_ delivered the same ultraviolet absorbance, but only 29% performance decay (Supplementary Fig. 57). In addition, BOC-VDWGs-AL displayed an almost identical V_O_^••^ density but a 134.4-fold higher PCR rate than BOC-VDWGs-76-V_O_^••^. Furthermore, Bi_3_O_4_Cl-VDWGs-AL-O_2_ showed a 35% performance decrease compared with Bi_3_O_4_Cl-VDWGs-AL, despite their equal absorbance at 420 nm (Supplementary Fig. 58). These crucial comparisons clearly support our view that VDWG-Bi-V_O_^••^-Bi is not the sole performance contributor, and further demonstrates that the VDWGs-induced low E_b_ and VDWG-Bi-V_O_^••^-Bi cooperatively lead to the superior CO_2_-to-CO photoreactivity observed in this study.

To further understand the roles of the two performance contributors, we produced a control sample that possesses commensurable VDWGs exposure percentage and V_O_^••^ concentration with BOC-VDWGs-AL, but its VDWGs are blocked with ions. This sample was realized by intercalating Li into the VDWGs of BOC-VDWGs-AL through a reported electrochemical lithiation scenario^64^, and hence named BOC-VDWGs-AL-Li. Although Li was able to fill the V_O_^••^, the in situ electro-reduction process taking place on the cathode where BOC-VDWGs-AL was located generates fresh V_O_^••^, so that the V_O_^••^ concentration was maintained, as confirmed by the nearly lossless V_O_^••^-attributed EPR peaks before and after the Li intercalation. It was found that the Li padding largely deteriorated the capacity of the VDWGs to weaken exciton binding, resulting in a high *E*_b_ of 117 meV and therefore a PCR rate of 3.7 μmol g^−1^ h^−1^ (Supplementary Fig. 59). Besides, we synthesized a (001)-dominated single-layered BiOCl nanosheet without VDWGs (Supplementary Fig. 60). Despite the higher amount of V_O_^••^, this sample delivered much lower PCR performance than BOC-VDWGs-AL. These phenomena suggest that in our PCR process, the VDWGs-mediated *E*_b_ modulation plays a more determinant role than the VDWG-Bi-V_O_^••^-Bi-promoted catalysis. Moreover, when using Ag as a co-catalyst and triethanolamine as a hole scavenger, BOC-VDWGs-AL delivered a PCR rate of 1.98 mmol g^−1^ h^−1^ (Supplementary Fig. 61).

To conclude, we successfully achieved an efficient PCR process for the production of CO using only visible light irradiation, pure water, CO_2_, and atomically thin VDWG-rich BiOCl nanosheets. Nanosheet synthesis was achieved via a generalizable gas-phase exfoliation process that was similar to the syngas synthetic route. The obtained nanosheets contained a conceptually graphene-analog configuration with its basic plane wrapped by VDWGs. Their high VDWG coverage proportion (99%) resulted in two key benefits in terms of the nanosheet properties, namely an ultralow exciton binding energy and catalytically active VDWG-Bi-V_O_^••^-Bi sites. These benefits promote exciton-to-electron and CO_2_-to-CO transitions, resulting in a superior pure water PCR rate of 188.2 μmol g^−1^ h^−1^. As previous works on layered materials have focused overwhelmingly on exfoliation to obtain ultrathin atomic layers (but with low-percentage VDWG coverages), defect engineering to create active sites, and doping and single atom loading to activate the covalently-bonded planes, our developed VDWG engineering strategy potentially adds a dimension to maximizing the performances of layered photocatalysts.

## Methods

### Synthesis of CBOC-VDWGs by our reported carbon-decoration methodology with partial modifications^34,35^

5.55 mmol of glucose was added into 60 mL of deionized water to form a lucid solution. 10 mmol of Bi(NO_3_)_3_•5H_2_O was added drop by drop under fierce stirring, further keeping the solution transparent. The mixed solution experienced vigorous ultrasonication actuated from an ultrasonic cell disrupter for 20 h. 10 mmol of KCl was added, followed by the addition of 2.5 mol L^−1^ KOH to tune the solution pH to 6.5. The mixture was transferred into an autoclave (Anhui Kemi Machinery Technology Co., Ltd) and heated, for the first stage at 160 °C for 6 h and for the second stage at 200 °C for 12 h. The resultant product was subjected to a plasma treatment to remove surface-absorbed carbonaceous species while keeping carbon species only within the lattices. For comparison, BOC-VDWGs-76 was synthesized under the same conditions but without glucose addition.

### Synthesis of BOC-VDWGs-AL by a syngas-synthesis-like reaction-driven, gas-phase exfoliation of CBOC-VDWGs

CBOC-VDWGs were put into 100 mL of deionized water and gently stirred for 36 h under Ar. The suspension solution was filtered to isolate the sample out of the solution. The resulting slurry was uniformly distributed inside a porcelain boat, which was subsequently placed in a tube furnace. The tube was bubbled with an Ar/H_2_O mixed gas and then thermally treated at 360 ^o^C for 6 h at a heating rate of 2 ^o^C min^−1^.

### Synthesis of BOC-VDWGs-AL-Li by a modified electrochemical lithiation method^48^

15 mg of BOC-VDWGs-AL was added into the mixture of 0.6 mL of deionized water, 0.5 mL of ethanol and 0.2 mL of Nafion (5 wt %) solution. The mixture was sonicated for 20 min and dropped onto a carbon paper. The slurry-coated carbon paper was placed within a culture dish with a cover until the water and ethanol were naturally evaporated, forming the final cathode. Li foil was used as anode and 1 mol L^−1^ LiPF_6_ as electrolyte. The electrochemical Li intercalation into BOC-VDWGs-AL was performed in a battery testing system at ambient conditions.

### Characterization

The X-ray diffraction (XRD) patterns of all sample powders were carried out by a Bruker D8 Advance diffractometer having monochromatized Cu Kα radiation with *λ* = 0.15418 nm. The morphologies were surveyed by a field-emission scanning electron microscope (FE-SEM; JEOL 6700-F), by a transmission electron microscope (TEM; Tecnai G2 F30, FEI), and by a high-resolution TEM (HRTEM; JEOL JSM-2010). Information on the atomic arrangements was obtained by a double-aberration-corrected high-angle annular dark-field scanning TEM (HAADF-STEM; JEOL JEM-2200FS). Chemical compositions and states were determined by X-ray photoelectron spectroscopy (XPS) (ThermoScientific ESCLAB 250Xi). All binding energies were referenced to the C 1 s peak (284.6 eV) arising from the adventitious carbon. Positron annihilation spectroscopy (PAS) was performed by a conventional ORTEC-265 fast–fast coincident system at room temperature. The thicknesses of all samples were measured by an atomic force microscopy (AFM, Dimension edge, Bruker). X-ray absorption fine structure (XAFS) spectra were performed, at beamline 1W1B station in Beijing Synchrotron Radiation Facility (BSRF) and at beamline14W1 station in Shanghai Synchrotron Radiation Facility (SSRF). Raman spectra were extracted from a confocal laser micro-Raman spectrometer (Thermo DXR Microscope, USA) excited by a 532 nm laser. Electron paramagnetic resonance (EPR) spectra were conducted by a Bruker EMX EPR Spectrometer (Billerica, MA). The photoluminescence (PL) and time-resolved fluorescence decay spectra (TRFL) were obtained on a steady-state and time-resolved Fluorescence Spectrometers (FLS1000, Edinburgh Instruments). In situ Fourier-transform infrared spectroscopy (FTIR) measurement was recorded by Nicolet iS50 spectrometer (Thermo, USA) with a MCT detector in a designed reaction cell. UV-visible diffused reflectance spectra of the samples were obtained for the dry-pressed film samples using a UV-visible spectrophotometer (UV-2550, Shimadzu, Japan) with BaSO_4_ as the reflectance standard.

### Photocatalytic CO_2_ reduction

The photocatalytic CO_2_ reduction experiments were conducted in an online system (Labsolar-6A, Beijing Perfectlight Technology Co., Ltd.). Before the photocatalytic CO_2_ reduction process, 50 mg of sample was suspended in 100 mL of deionized water with high-purity CO_2_ gas bubbled through the solution for one hour. The visible light source was supplied by a 300 W Xe lamp with a 400 nm cut-filter to filter UV radiations. The temperature of the whole reaction system was kept around 20 ± 0.5 °C through a recirculating cooling water system.

During the light irradiation, the gas products were qualitatively analyzed by gas chromatograph equipped with two gas analysis channels. The first channel analyzed hydrocarbons, using a HP PLOT Al_2_O_3_ column for separations, He as the carrier gas and a flame ionization detector (FID). The second channel analyzed CO_2_, N_2_, Ar, O_2_, CH_4_, and CO, using a micropacket Haysep Q, H-N column and a Molsieve 13× column for separations, He as carrier gas and a thermal conductivity detector (TCD).

### Apparent quantum yield (AQY)

The AQY was calculated by the well-established equation:

$${{{{{\rm{AQY}}}}}}( \% )\, =	\,\frac{{{{{{\rm{number}}}}}}\,{{{{{\rm{of}}}}}}\,{{{{{\rm{reacted}}}}}}\,{{{{{\rm{electrons}}}}}}}{{{{{{\rm{number}}}}}}\,{{{{{\rm{of}}}}}}\,{{{{{\rm{incident}}}}}}\,{{{{{\rm{photos}}}}}}}\times 100 \% \\ \,=	\,\frac{2\,\times\,{{{{{\rm{number}}}}}}\,{{{{{\rm{of}}}}}}\,{{{{{\rm{evolved}}}}}}\,{{{{{\rm{CO}}}}}}\,{{{{{\rm{molecules}}}}}}}{{{{{{\rm{number}}}}}}\,{{{{{\rm{of}}}}}}\,{{{{{\rm{incident}}}}}}\,{{{{{\rm{photos}}}}}}}\times 100 \%$$

The number of incident photons was calculated according to the equation:

$$N\,=\,(E)/(hc)$$

where *E*, *λ*, *h*, and *c* are the energy of incident photos, the wavelength of the incident monochromatic light, the Planck constant, and the light speed, respectively.

### ^13^C isotopic labeling experiment

The ^13^C isotopic labeling experiment was also carried out in an online system (Labsolar-6A, PerfectLight). Before irradiation, 50 mg of sample was suspended in 100 mL of deionized water with high-purity ^13^CO_2_ gas bubbled through the solution for one hour. The visible light source was supplied by a 300 W Xe lamp with a 400 nm cut-filter to filter UV radiations. The temperature of the whole reaction system was kept around 20 ± 0.5 °C through a recirculating cooling water system. The gas products were analyzed by gas chromatography-mass spectrometry. The column (HP-MOLESIEVE, 30 m × 0.32 mm × 25 μm, Agilent Technologies, USA) was used for detecting ^13^CO.

### Turnover number

The turnovers number (TON) of BOC−VDWGs−76, CBOC−VDWGs and BOC−VDWGs−AL was re-calculated by the following equations reported by Xie et al.^65^:

$${{{{{{\rm{TON}}}}}}}_{{{{{{{\rm{CO}}}}}}}_{2}-{{{{{\rm{CO}}}}}}}=\frac{{{{{{\rm{Number}}}}}}\,{{{{{\rm{of}}}}}}\,{{{{{\rm{reacted}}}}}}\,{{{{{\rm{electrons}}}}}}}{{{{{{\rm{Number}}}}}}\,{{{{{\rm{of}}}}}}\,{{{{{\rm{active}}}}}}\,{{{{{\rm{sites}}}}}}}$$

$${{{{{\rm{Number}}}}}}\,{{{{{\rm{of}}}}}}\,{{{{{\rm{active}}}}}}\,{{{{{\rm{sites}}}}}}=\frac{2* {{m}}* {S}_{{{{{{\rm{BET}}}}}}}* {N}_{{{{{{\mathrm{O}}}}}}}* {P}_{{{{{{\rm{Oxygen}}}}}}\,{{{{{\rm{vacancy}}}}}}}}{{S}_{{{{{{\rm{unit}}}}}}\,{{{{{\rm{cell}}}}}}}}$$

In these questions, *m* is the mass of the catalysts used in the photocatalytic reaction system, *N*_O_ is the number of surface oxygen atoms in the (010)-BiOCl per unit cell, 2 refers as one oxygen vacancy creates two Bi active sites, *P*_Oxygen vacancy_ is the proportion of oxygen vacancy calculated from the high-resolution O1*s* XPS spectra, *S*_BET_ is the surface area determined by BET surface area plot and *S*_*unit cell*_ is the theoretical surface area of the (010)-BiOCl unit cell.

## Supplementary information

Supplementary Information

## Data Availability

All data generated in this study are provided in the Article and Supplementary Information. The other data that support the findings of this study are available from the corresponding author upon reasonable request.
