# Supplementary material for: Van Der Waals gap-rich BiOCl atomic layers realizing efficient, pure-water CO2-to-CO photocatalysis
Source: Nat Commun. 2021 Oct 11;12:5923. doi: 10.1038/s41467-021-26219-6 (PMC8505634; doi:10.1038/s41467-021-26219-6)
Supplement: Supplementary file 1 — Supplementary Information [file 41467_2021_26219_MOESM1_ESM.pdf]

Supplementary Information for

Van Der Waals Gap-Rich BiOCl Atomic Layers Realizing Efficient, Pure-Water CO_2-to-CO Photocatalysis

Yanbiao Shi[1‡], Jie Li[1‡*], Chengliang Mao[1‡], Song Liu[2], Xiaobing Wang[1], Xiufan Liu[1], Shengxi Zhao[1], Xiao Liu[1], Yanqiang Huang[2] & Lizhi Zhang[1*]

[1]*Key Laboratory of Pesticide & Chemical Biology of Ministry of Education, Institute of Environmental & Applied Chemistry, College of Chemistry, Central China Normal University, 152 Luoyu Road, Wuhan 430079, China*

[2]*State Key Laboratory of Catalysis, Dalian Institute of Chemical Physics, Chinese Academy of Sciences, Dalian 116023, China*

[‡]*These authors contributed equally: Yanbiao Shi, Jie Li, Chengliang Mao*

[*]Correspondence and requests for materials should be addressed to Jie Li (leejay20102010@163.com) or Lizhi Zhang (zhanglz@mail.ccnu.edu.cn)

Supplementary Figures

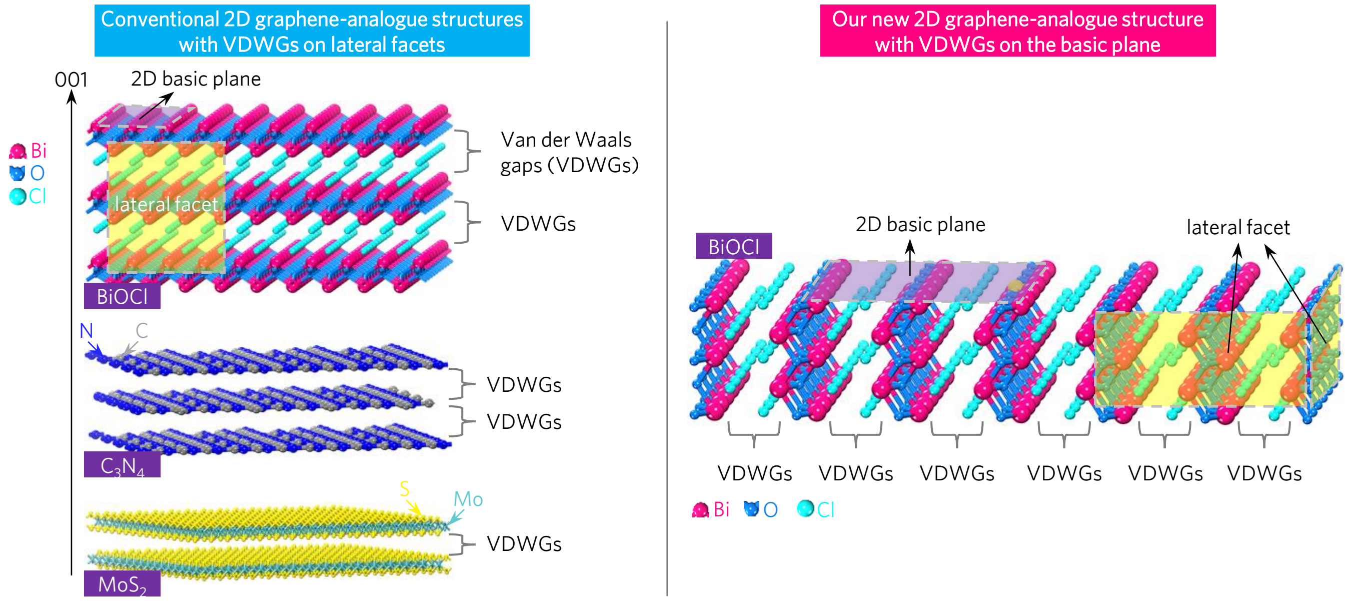

Supplementary Figure 1. Schematic illustration of the structures of conventional 2D graphene-analogues and our developed 2D graphene-analogue. Conventional 2D graphene-analogues have a preferential exposure of (001)-faceted nanosheets with VDWGs on their lateral facets. This means that the exposure ratio of VDWGs in conventional 2D graphene-analogues is very low. Our developed 2D graphene-analogue has abundant VDWGs on the 2D basic plane. It is worth noting that, although exposing VDWGs on 2D basic planes has been enabled on BiOCl nanosheets with thinkness larger than 35 nm, we deliver the report that realizes the exposure of VDWGs on the dominant facet of an ultrathin nanosheet with thickness less than 3 nm.

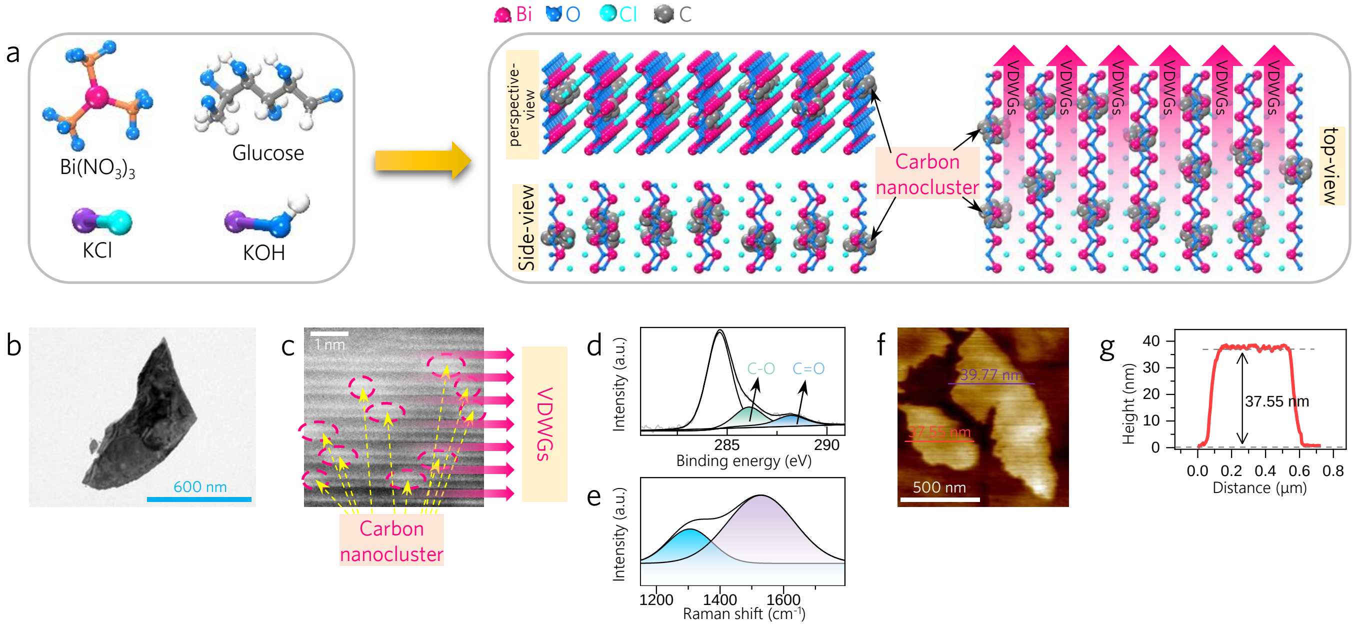

Supplementary Figure 2. Characterizations of CBOC-VDWGs. (a) Schematic illustration of the synthetic process and crystalline structure of CBOC-VDWGs. (b) TEM image, (c) quasi-atomic-resolved HAADF-STEM image, (d) C 1*s* XPS, (e) Raman spectrum, (f) AFM image, and (g) intensity profile (taken along the line in image f) of CBOC-VDWGs. The XPS result reveals clear carbon species in CBOC-VDWGs. As indicated from the Raman peaks emerging at 1343 and 1586 cm^{-1}, the carbon species exist as carbon nanoclusters.

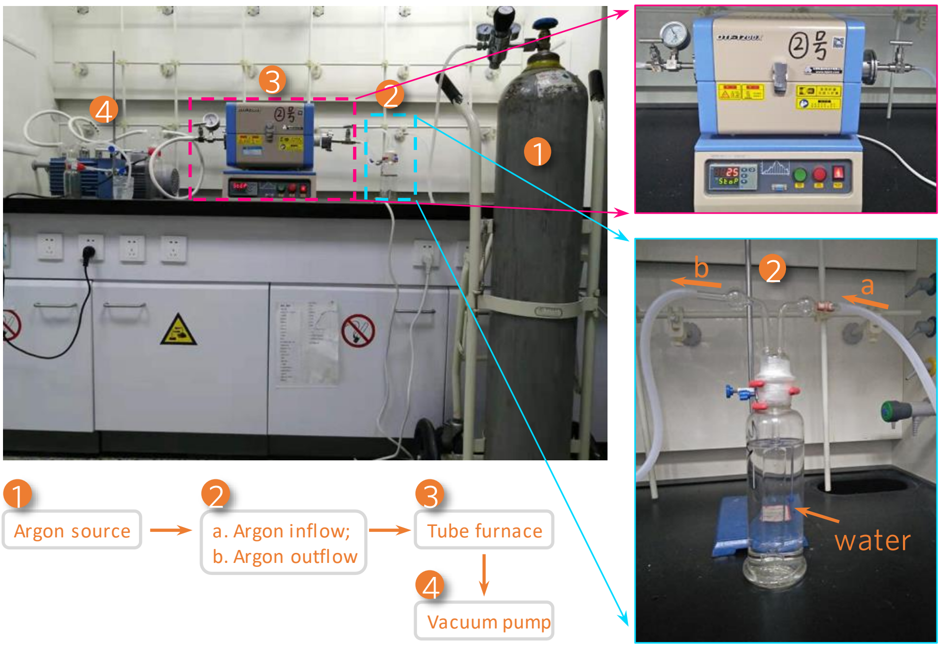

Supplementary Figure 3. Photograph of the entire equipment used for the syngas-synthesis-like reaction-driven, gas-phase exfoliation. Ar gas goes through a water-contained scrubbing bottle to form a mixture of Ar and water.

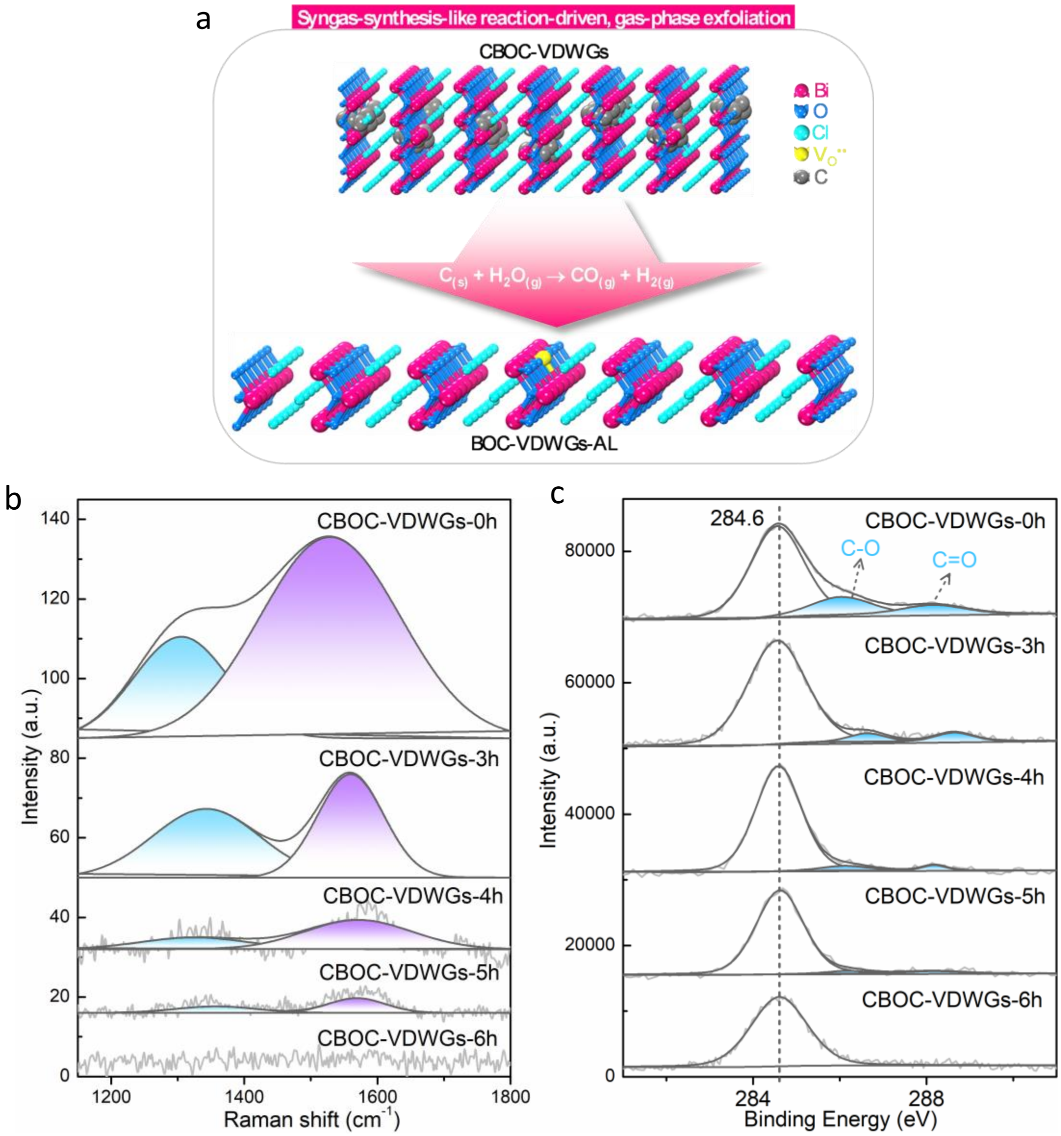

Supplementary Figure 4. (a) Schematic illustration of the syngas-synthesis-like reaction-driven, gas-phase exfoliation of CBOC-VDWGs into BOC-VDWGs-AL. (b) Raman spectra and (c) C 1*s* XPS spectra of CBOC-VDWGs via our developed syngas-synthesis-like reaction-driven, gas-phase exfoliation method. After reacting in Ar/H_2O atmosphere for 6h, the lattice-embedded carbon clusters of CBOC-VDWGs were completely removed, thus CBOC-VDWGs-6h (BOC-VDWGs-AL) was obtained.

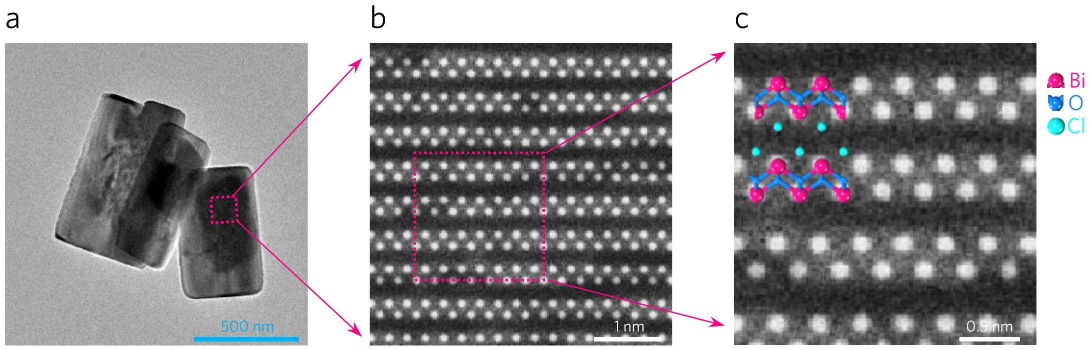

Supplementary Figure 5. (a) TEM and (b,c) HAADF-STEM images of BOC-VDWGs-76.

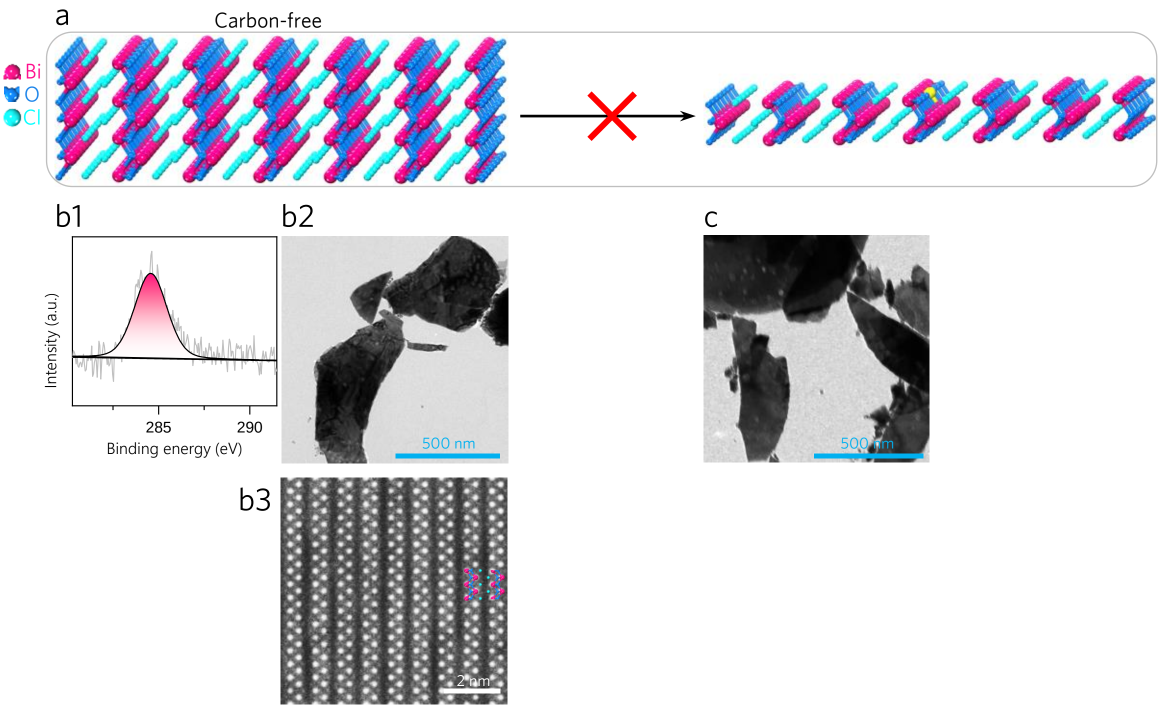

Supplementary Figure 6. Demonstration of the importance of carbon species in the our newly-developed syngas-synthesis-like reaction-driven, gas-phase exfoliation. (a) Schematic illustration of the exfoliation of CBOC-VDWGs-O_2. (b1) XPS, (b2) TEM image and (b3) HAADF-STEM images of CBOC-VDWGs-O_2. (c) TEM image of the exfoliated product of CBOC-VDWGs-O_2. CBOC-VDWGs-O_2 was synthesized by calcination of CBOC-VDWGs in O_2 at 450 ^oC for 8 hours. The XPS result shows that CBOC-VDWGs-O_2 owns no carbon species. The TEM images indicate that, even using the syngas-synthesis-like reaction-driven, gas-phase exfoliation, CBOC-VDWGs-O_2 cannot be exfoliated into ultrathin nanosheet. These results demonstrate the crucial role that the carbon species play in the process of our new exfoliation strategy.

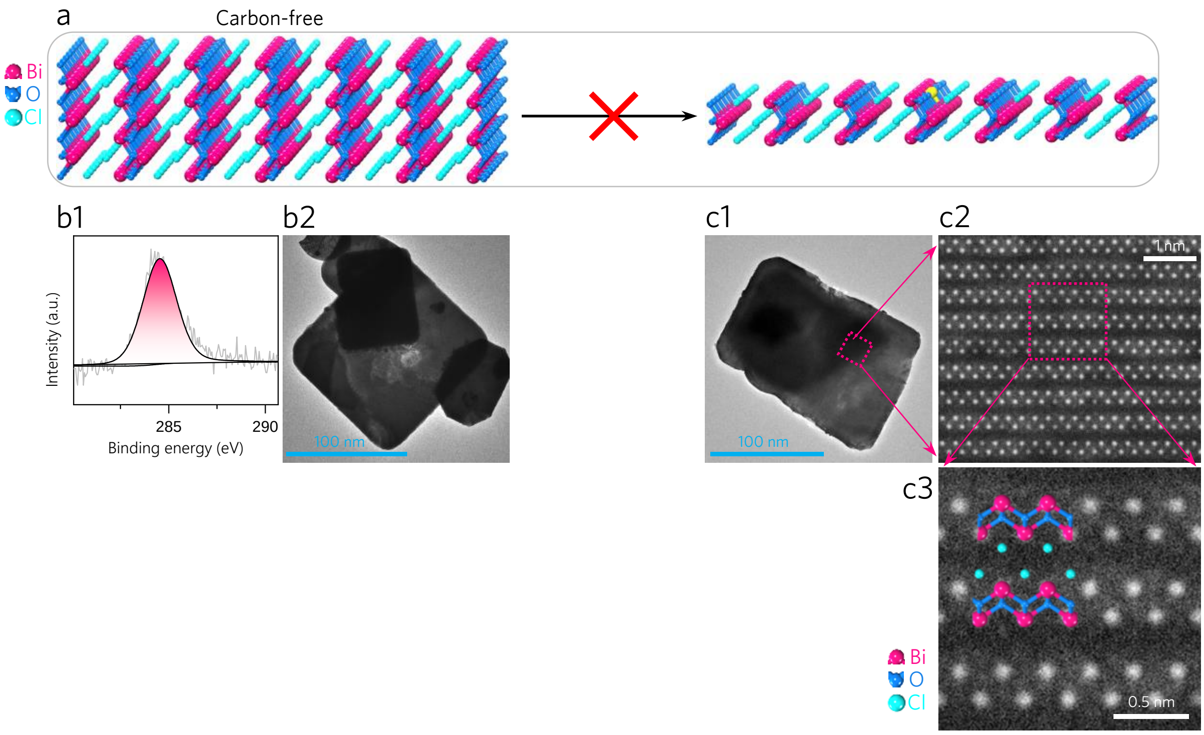

Supplementary Figure 7. Demonstration of the importance of carbon species in our developed syngas-synthesis-like reaction-driven, gas-phase exfoliation. (a) Schematic illustration of the exfoliation of BOC-VDWGs-76. (b1) XPS and (b2) TEM image of BOC-VDWGs-76. (c) TEM and HAADF-STEM images of the exfoliated product of BOC-VDWGs-76. The precursors used for synthesizing BOC-VDWGs-76 are all carbon-free. The XPS result shows that BOC-VDWGs-76 contains no carbon species. The TEM images indicate that, even using the syngas-synthesis-like reaction-driven, gas-phase exfoliation, BOC-VDWGs-76 cannot be exfoliated into ultrathin nanosheet. These results demonstrate the crucial role that the carbon species play in the process of our new exfoliation strategy.

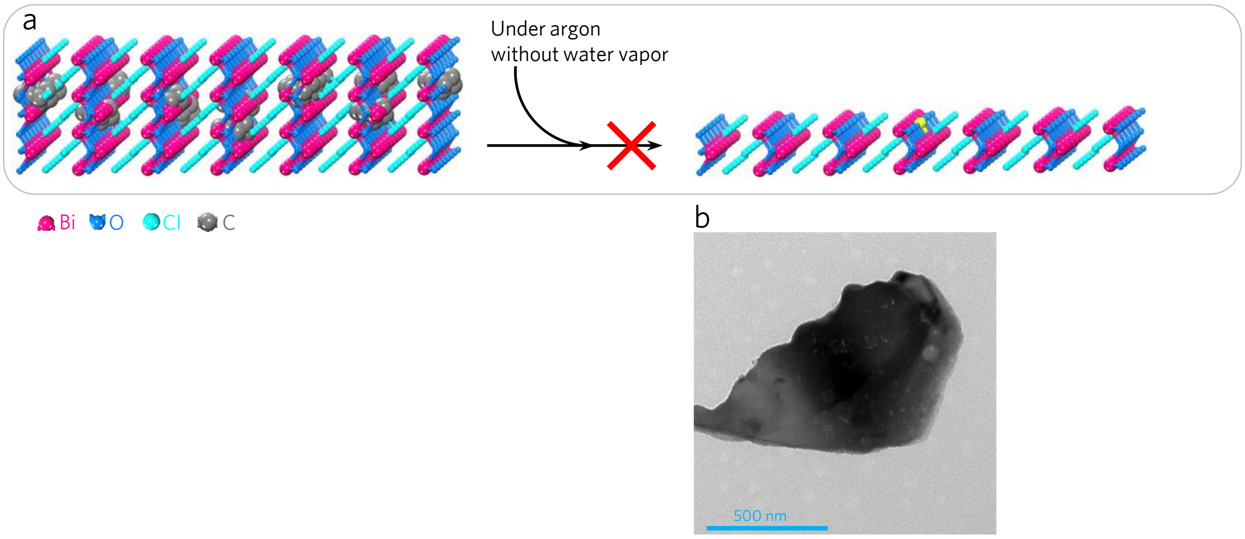

Supplementary Figure 8. Demonstration of the importance of water vapor in the our newly-developed syngas-synthesis-like reaction-driven, gas-phase exfoliation. (a) Schematic illustration of the exfoliation of CBOC-VDWGs in the absence of water vapor. (b) TEM image of the exfoliated product of CBOC-VDWGs in the absence of water vapor. The TEM images indicate that, when water vapor is in absence, CBOC-VDWGs cannot be exfoliated into ultrathin nanosheet. These results confirm the reaction happening between carbon species and water vapor.

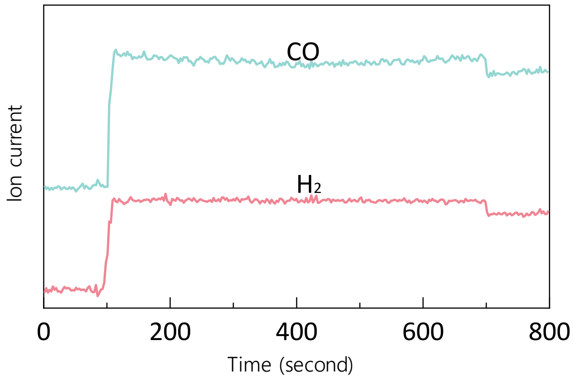

Supplementary Figure 9. The compositions of the gases generated during the exfoliation process determined by gas analysis mass spectrometer. This provides evidence for the exfoliation reaction following $C_{(s)} + H_2O_{(g)} \rightarrow CO_{(g)} + H_{2(g)}$.

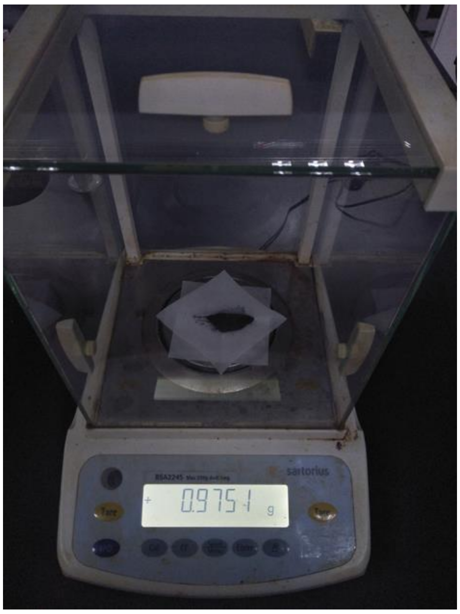

Supplementary Figure 10. Photograph of the product after our developed syngas-synthesis-like reaction-driven, gas-phase exfoliation.

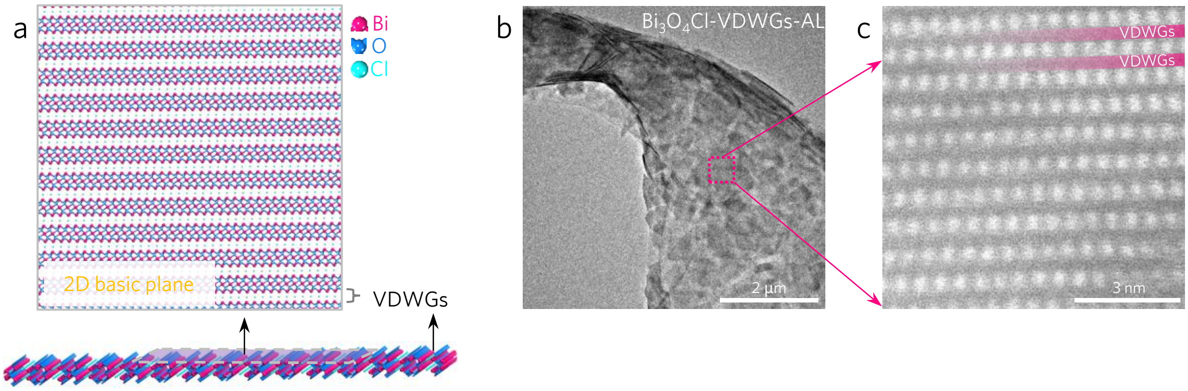

Supplementary Figure 11. Characterizations of Bi_3O_4Cl-VDWGs-AL. (a) Schematic illustration of the crystalline structure of Bi_3O_4Cl-VDWGs-AL. (b) TEM image and (c) quasi-atomic-resolved HAADF-STEM image of Bi_3O_4Cl-VDWGs-AL. Bi_3O_4Cl-VDWGs-AL has the morphology of an ultrathin nanosheet. Clear VDWGs were observed on the two-dimensional facets. These results demonstrate that our syngas-synthesis-like reaction-driven, gas-phase exfoliation can be applicable to Bi_3O_4Cl.

Carbon-modified Bi_3O_4Cl was synthesized by hydrothermal treatment of the mixed solution (pH = 10.6) of glucose, $Bi(NO_3)_3$•$5H_2O$, and KCl at 180 °C for 24 hours. The carbon-modified Bi_3O_4Cl were then subjected to the syngas-synthesis-like reaction-driven, gas-phase exfoliation with the operating parameters with which BOC-VDWGs-AL was synthesized. The observed atomic columns consisting of bright spots are [Bi_3O_4] layers, because Bi has the highest atomic number (83) than that of O (8) and Cl (17). Two neighboring [Bi_3O_4Cl] layers are stacked together via van der Waals force between [Cl] layers. Therefore, the dark region between two [Bi_3O_4] layers could be identified as VDWGs.

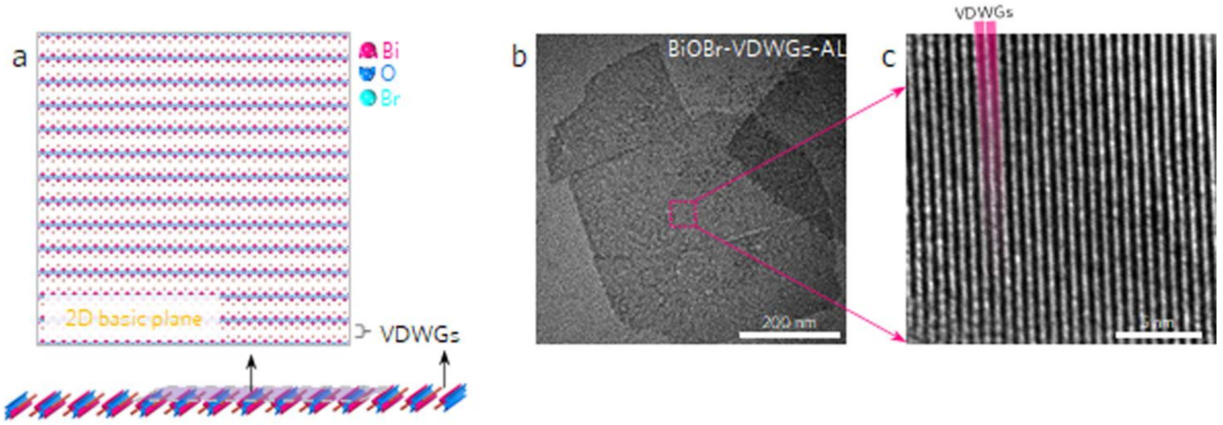

Supplementary Figure 12. Characterizations of BiOBr-VDWGs-AL. (a) Schematic illustration of the crystalline structure of BiOBr-VDWGs-AL. (b) TEM image and (c) quasi-atomic-resolved HAADF-STEM image of BiOBr-VDWGs-AL. BiOBr-VDWGs-AL has the morphology of an ultrathin nanosheet. Clear VDWGs were observed on the two-dimensional facets. These results demonstrate that our syngas-synthesis-like reaction-driven, gas-phase exfoliation can be applicable to BiOBr.

Carbon-modified BiOBr was synthesized by solvothermal treatment of ethylene glycol (pH = 6.5) containing glucose, $Bi(NO_3)_3{\bullet}5H_2O$, and KBr at 160 °C for 18 hours. The carbon-modified BiOBr were then subjected to the syngas-synthesis-like reaction-driven, gas-phase exfoliation with the operating parameters with which BOC-VDWGs-AL was synthesized. The observed atomic columns consisting of bright spots are [Bi_2O_2] layers, because Bi has the highest atomic number (83) than that of O (8) and Br (35). Two neighboring [BiOBr] layers are stacked together via van der Waals force between [Br] layers. Therefore, the dark region between two [Bi_2O_2] layers could be identified as VDWGs.

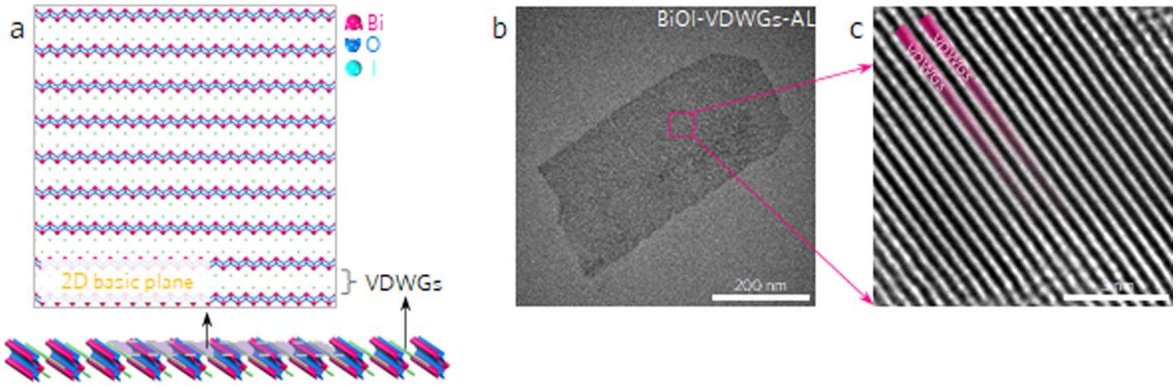

Supplementary Figure 13. Characterizations of BiOI-VDWGs-AL. (a) Schematic illustration of the crystalline structure of BiOI-VDWGs-AL. (b) TEM image and (c) quasi-atomic-resolved HAADF-STEM image of BiOI-VDWGs-AL. BiOI-VDWGs-AL has the morphology of an ultrathin nanosheet. Clear VDWGs were observed on the two-dimensional facets. These results demonstrate that our syngas-synthesis-like reaction-driven, gas-phase exfoliation can be applicable to BiOI.

Carbon-modified BiOI was synthesized by hydrothermal treatment of the mixed solution (pH = 6.5) of water and ethanol containing glucose, $Bi(NO_3)_3{\bullet}5H_2O$, and KI at 120 °C for 12 hours. The carbon-modified BiOI were then subjected to the syngas-synthesis-like reaction-driven, gas-phase exfoliation with the operating parameters with which BOC-VDWGs-AL was synthesized. The observed atomic columns consisting of bright spots are [Bi_2O_2] layers, because Bi has the highest atomic number (83) than that of O (8) and I (53). Two neighboring [BiOI] layers are stacked together via van der Waals force between [I] layers. Therefore, the dark region between two [Bi_2O_2] layers could be identified as VDWGs.

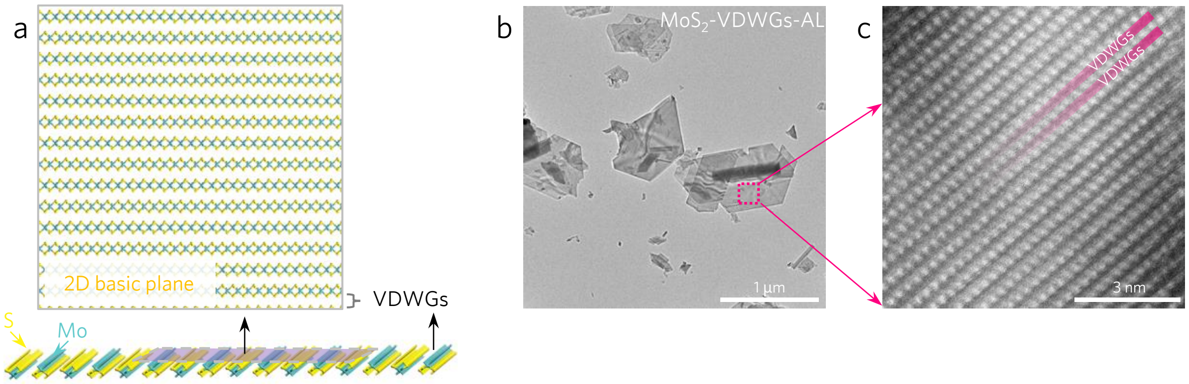

Supplementary Figure 14. Characterizations of MoS_2-VDWGs-AL. (a) Schematic illustration of the crystalline structure of MoS_2-VDWGs-AL. (b) TEM image and (c) quasi-atomic-resolved HAADF-STEM image of MoS_2-VDWGs-AL. MoS_2-VDWGs-AL has the morphology of an ultrathin nanosheet. Clear VDWGs were observed on the two-dimensional facets. These results demonstrate that our syngas-synthesis-like reaction-driven, gas-phase exfoliation can be applicable to MoS_2.

Carbon-modified MoS_2 was synthesized by hydrothermal treatment of the mixed solution of glucose, $(NH_4)_6Mo_7O_{24}\cdot 4H_2O$, and thiourea at 220 °C for 36 hours. The carbon-modified MoS_2 were then subjected to the syngas-synthesis-like reaction-driven, gas-phase exfoliation with the operating parameters with which BOC-VDWGs-AL was synthesized. The observed atomic columns consisting of bright spots are [Mo] layers, because Mo has the higher atomic number (42) than that of S (16). Two neighboring [MoS_2] layers are stacked together via van der Waals force between [S] layers. Therefore, the dark region between two [MoS_2] layers could be identified as VDWGs.

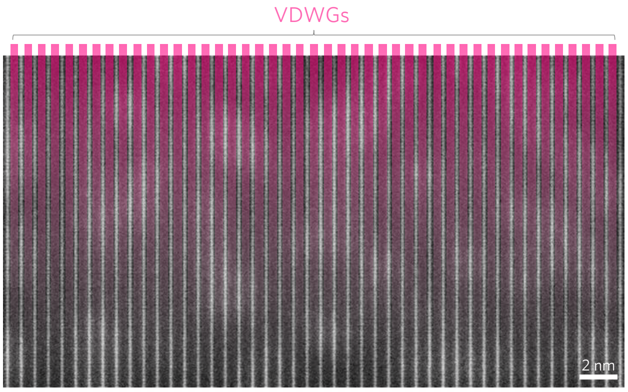

Supplementary Figure 15. Large-area HAADF-STEM image of MoS_2-VDWGs-AL.

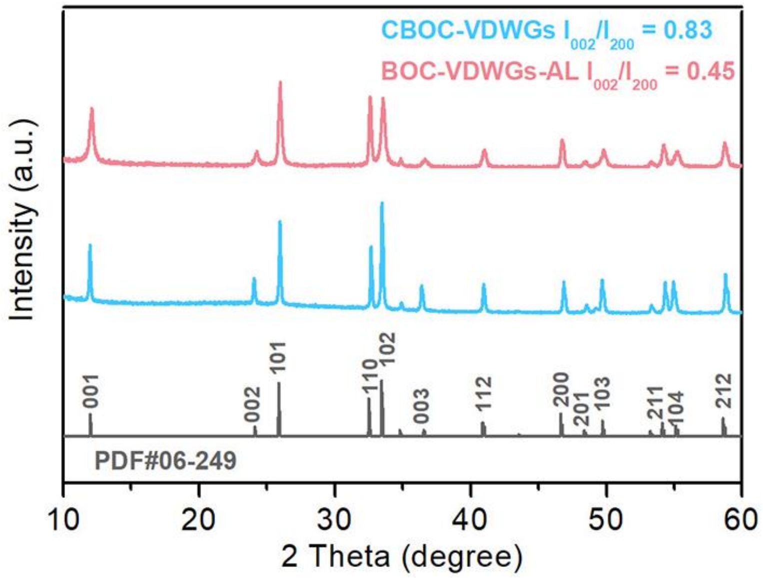

Supplementary Figure 16. XRD patterns of CBOC-VDWGs and BOC-VDWGs-AL. The inset shows the ratios of the intensity of (002) to (200) peaks. The lower ratio indicates more exposure of (010) facets that have abundant VDWGs.

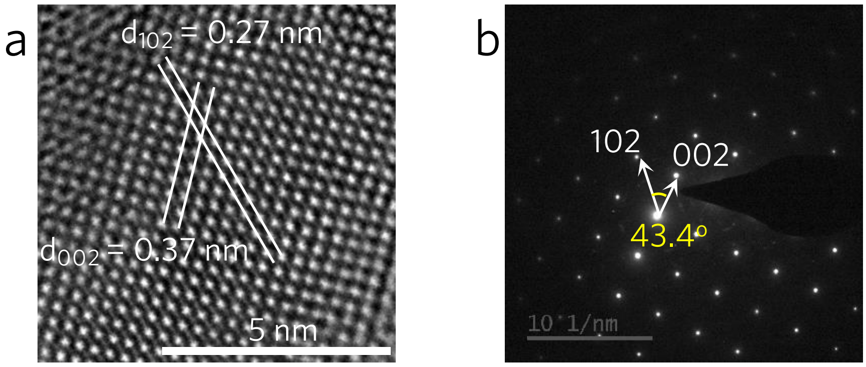

Supplementary Figure 17. (a) HRTEM image and (b) SAED pattern of BOC-VDWGs-AL.

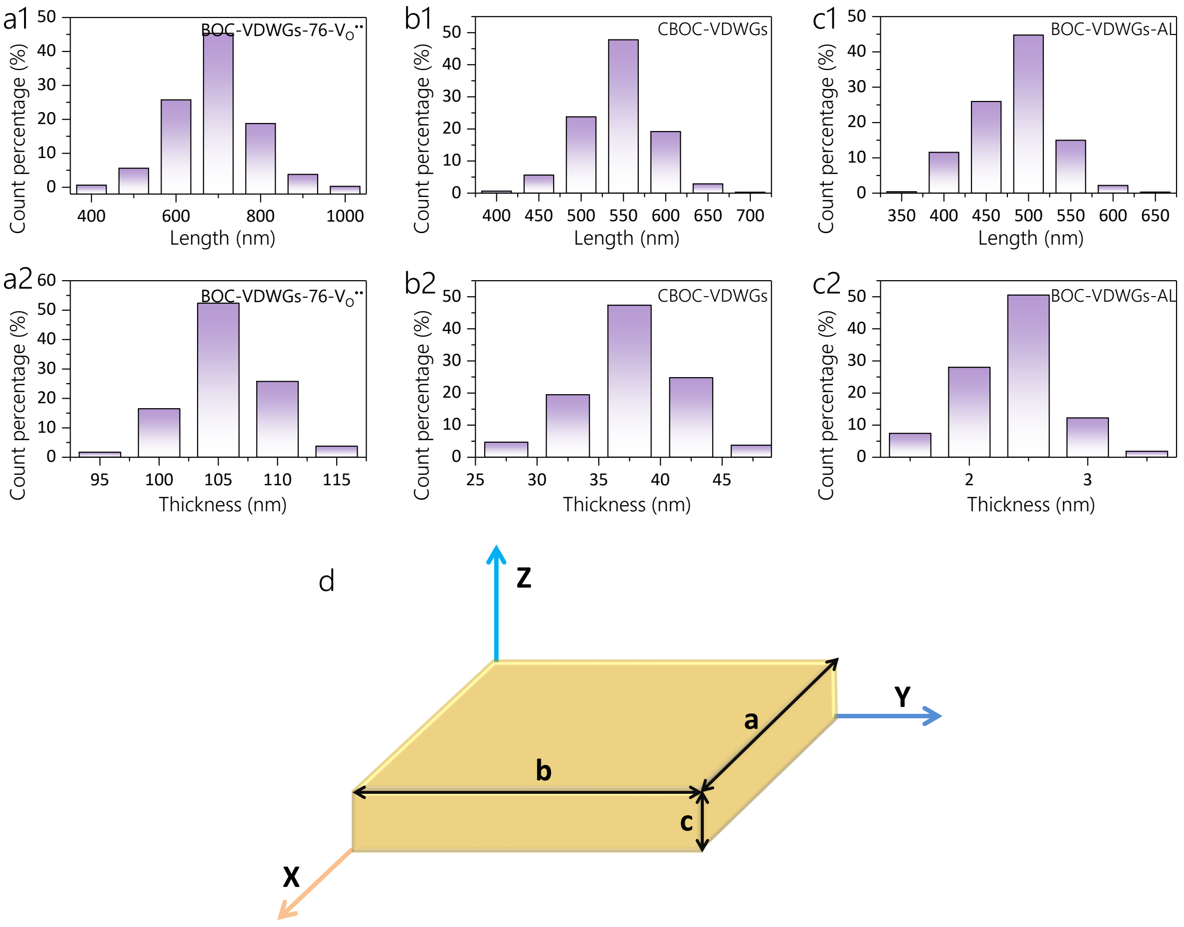

$$\textbf{Percentages of \{010\} facets} = \frac{ab}{ab + ac + bc} \times 100\%$$

Supplementary Figure 18. (a-c) Statistical results of the length and thickness of BOC-VDWGs-76-$V_O^{\bullet\bullet}$, CBOC-VDWGs, and BOC-VDWGs-AL. (d) Schematic illustration of the method used for calculating the percentage of (010) facet exposure. In this calculation method, we assumed $a = b$, since the as-prepared nanosheets are irregular in 2D shape. a and b represent the length of the nanosheets, while c is the thickness. The values of the lengths and thicknesses of the nanosheets were extracted from their SEM and TEM images.

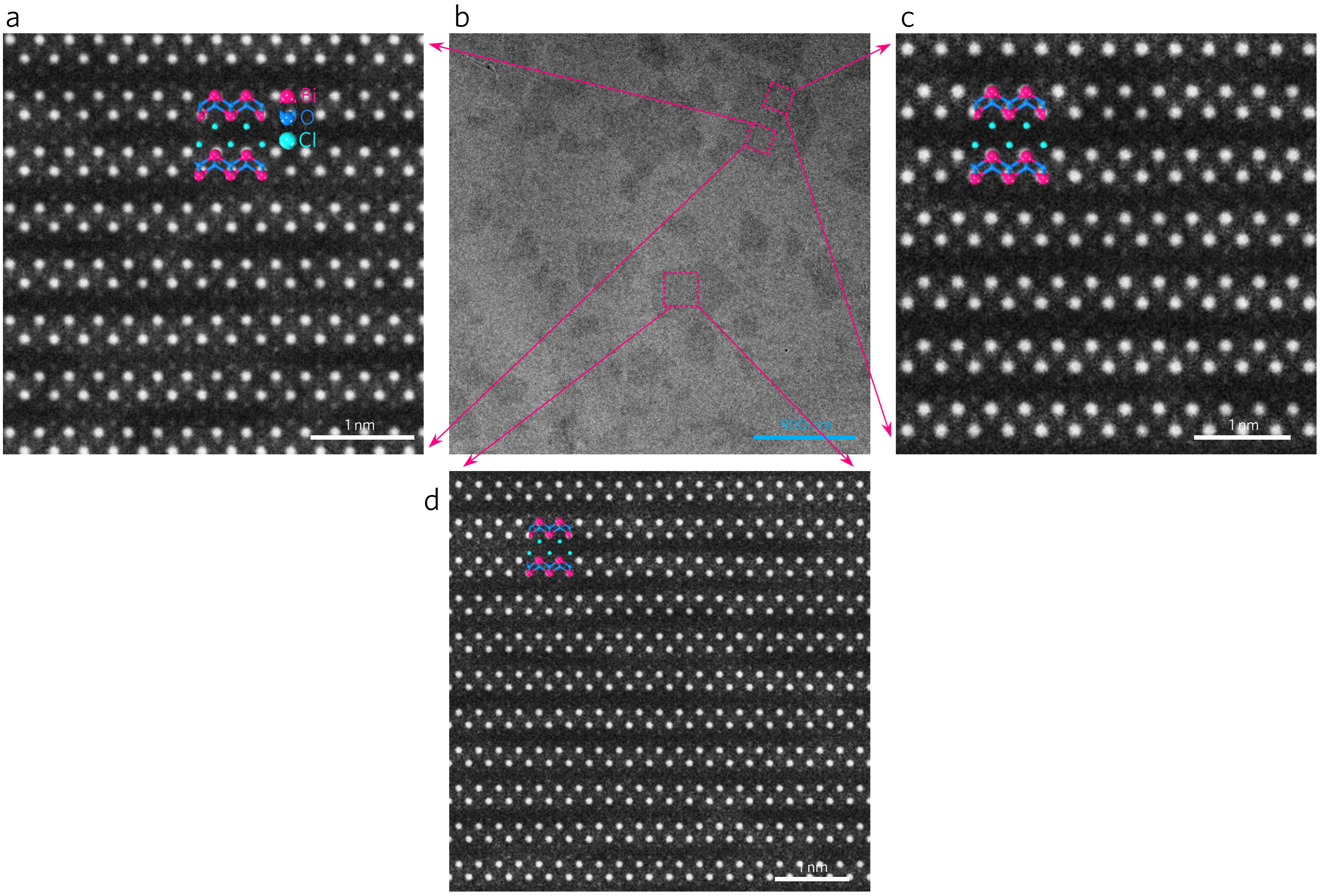

Supplementary Figure 19. (a,c,d) HAADF-STEM and (b) TEM images of BOC-VDWGs-AL.

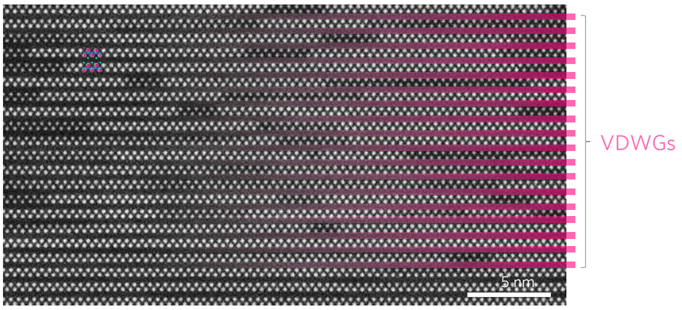

Supplementary Figure 20. Large-area HAADF-STEM image of BOC-VDWGs-AL.

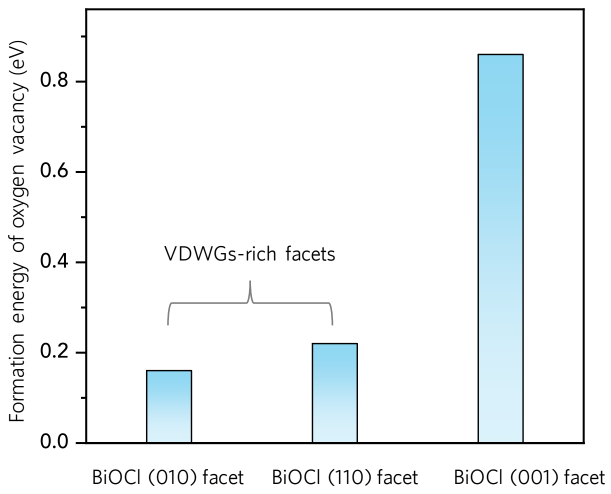

Supplementary Figure 21. Comparison of the calculated formation energies of oxygen vacancy on BiOCl (010), (110), and (001) facets.

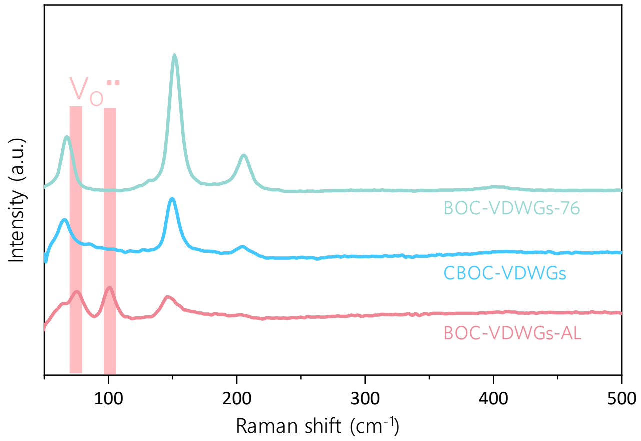

Supplementary Figure 22. Raman spectra of BOC-VDWGs-AL, CBOC-VDWGs, and BOC-VDWGs-76.

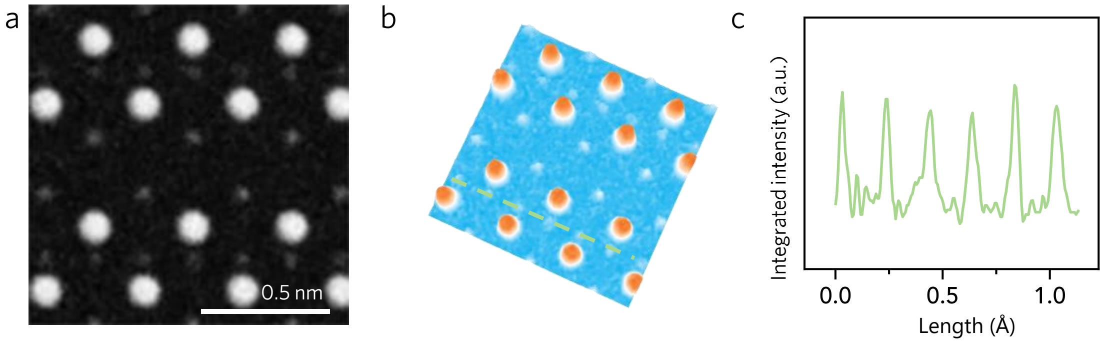

Supplementary Figure 23. Characterization of $V_O^{\bullet\bullet}$-free BOC-VDWGs-AL. (a) HAADF-STEM images, (b) 3D topographic color-coded intensity image (converted from **a**), and (c) intensity profile of BOC-VDWGs-AL-O_2. BOC-VDWGs-AL-O_2 was synthesized by calcining BOC-VDWGs-AL under O_2.

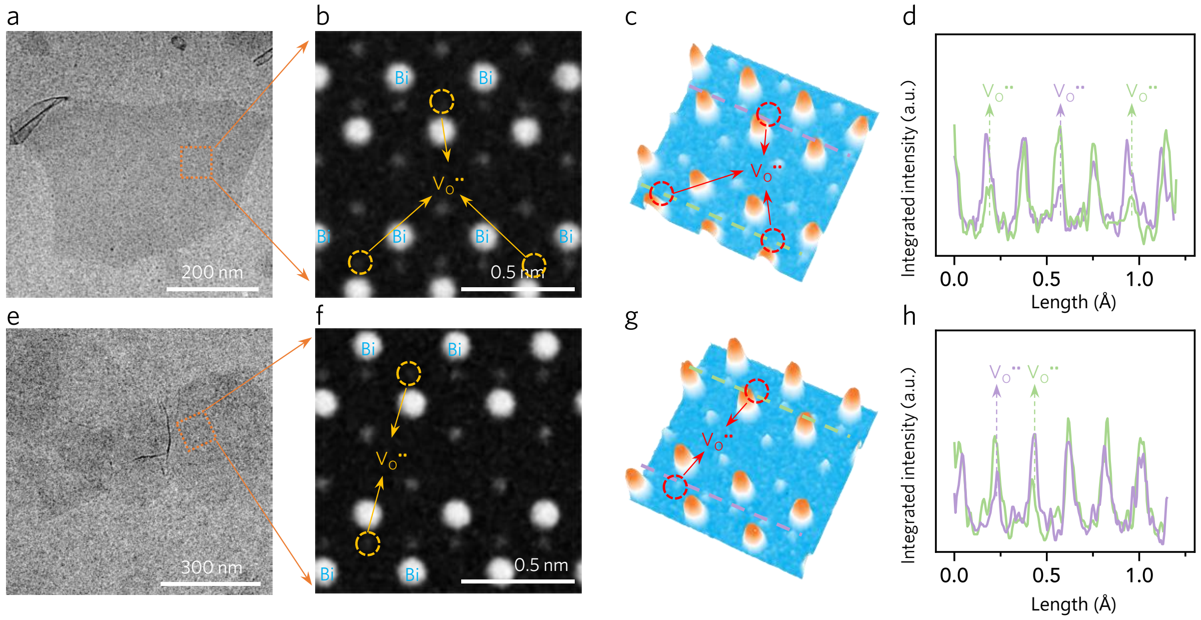

Supplementary Figure 24. Using more BOC-VDWGs-AL samples to confirm the presence of VDWG-Bi-$V_O^{\bullet\bullet}$-Bi defect. (a,e) TEM images, (b,f) HAADF-STEM images, (c,g) 3D topographic color-coded intensity images (converted from **b** and **f**), and (d,h) intensity profiles of another two batches of BOC-VDWGs-AL samples.

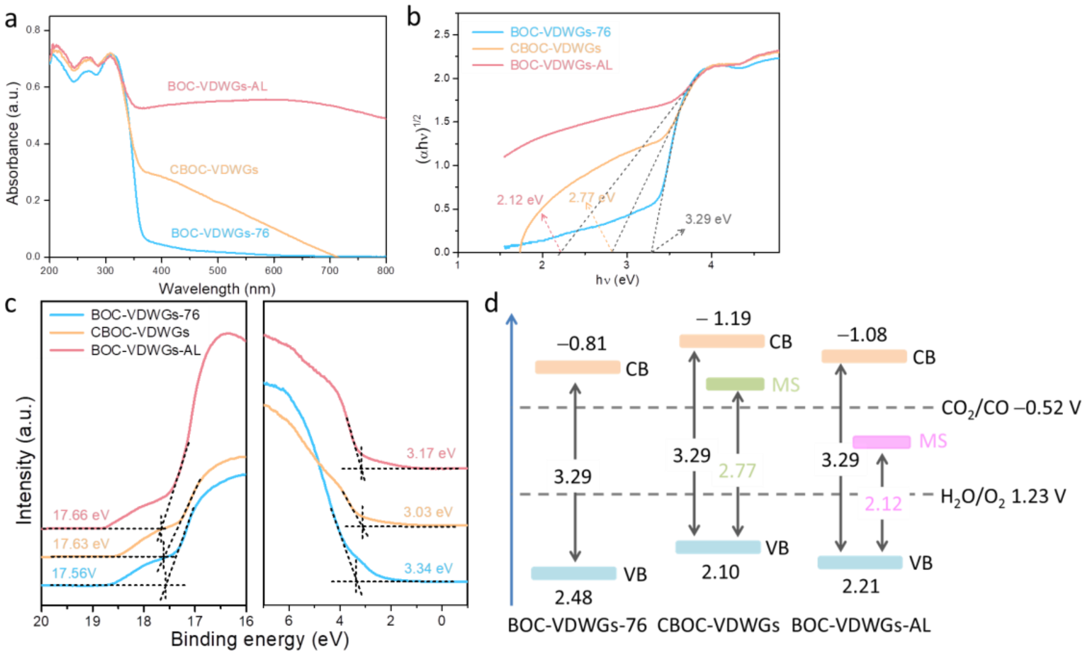

Supplementary Figure 25. (a) UV-visible absorption spectra, (b) plots of $(\alpha h\nu)^{1/2}$ versus energy (hν) for the band-gap energies, (c) valence-band XPS spectra, and (d) band structure alignments of BOC-VDWGs-AL and BOC-VDWGs-76.

According to the UV-visible absorption spectra, BOC–VDWGs–76, CBOC–VDWGs and BOC–VDWGs–AL have the same band gap, which is about 3.29 eV based on the Kubelka-Munk-plot (Supplementary Figure 25a-b). The step-like absorption tail extending to 700 nm appears in the UV-visible DRS of the CBOC–VDWGs, which is ascribed to the carbon incorporation-related midgap state (MS). Moreover, a bandgap of 2.77 eV, corresponding to the electronic transition between the valence bands (VB) and MS, was identified for the CBOC–VDWGs (*Nat. Energy*, *2021*, *6*, *388–397*). As for BOC–VDWGs–AL, a step-like absorption tail covers the whole visible light region, indicating that defect-related MS with a bandgap of 2.12 eV is generated in the forbidden band of BOC–VDWGs–AL.

Meanwhile, the VBs of BOC–VDWGs–76, CBOC–VDWGs and BOC–VDWGs–AL were measured by ultraviolet photoelectron spectra (UPS) because the correlation between the photoelectrode potential and the absolute potential of electrons has already been established (*Pure Appl. Chem. 1986*, *58*, *955-966; J. Phys. Chem. B 2003*, *107*, *1798-1803*). UPS was carried out in an ultrahigh vacuum (UHV) apparatus of a PHI5000 VersaProbe III (Scanning ESCA Microprobe) electron energy spectrometer with a base pressure of $< 5 \times 10^{-2}$ mbar. The sample was cleaned several times by argon–ion sputtering (1000 eV, 60 min) to remove the surface contaminants. UPS was measured using He I excitation (21.2 eV) and recorded with a constant pass bias of –5 eV. The overall resolution of the measurement was 0.04 eV. All of the measurements were carried out at ambient temperature. As demonstrated in Supplementary Figure 25c, the end energy level (EEL) of BOC–VDWGs–76, CBOC–VDWGs and BOC–VDWGs–AL are 17.56, 17.63 and 17.66 eV, respectively. Thus, based on the Fermi level (E_F) and equation of $\Phi = 21.2 - \text{EEL}$ (*Acta Phys. Sin. 2002*, *51*, *2644*), the work functions (Φ) of BOC-VDWGs-76, CBOC-VDWGs

and BOC-VDWGs-AL, were determined to be 3.64, 3.57 and 3.54 eV, respectively. The valence-band spectra revealed that the VB of BOC−VDWGs−76, CBOC−VDWGs and BOC−VDWGs−AL is 3.34, 3.03 and 3.17 eV below the Fermi level (EF), respectively. Thus, VB of the BOC−VDWGs−76, CBOC−VDWGs and BOC−VDWGs−AL is determined as 6.98, 6.60 and 6.71 eV vs. vacuum level, respectively. The relationship between the absolute electron potential of an electrode (E_{abs}) and the standard electrode potential (E°) is expressed as follows: $E_{abs} = - E° - 4.50$ (298 K), where the electron energy is 0 eV in a vacuum (*J. Phys. Chem. C 2007, 111, 4727-4733*). Therefore, the VB of BOC−VDWGs−76, CBOC−VDWGs and BOC−VDWGs−AL is estimated to be 2.48, 2.10, and 2.21 eV (vs NHE), respectively. The band structure of samples was portrayed by the band gap and VB obtained from UPS.

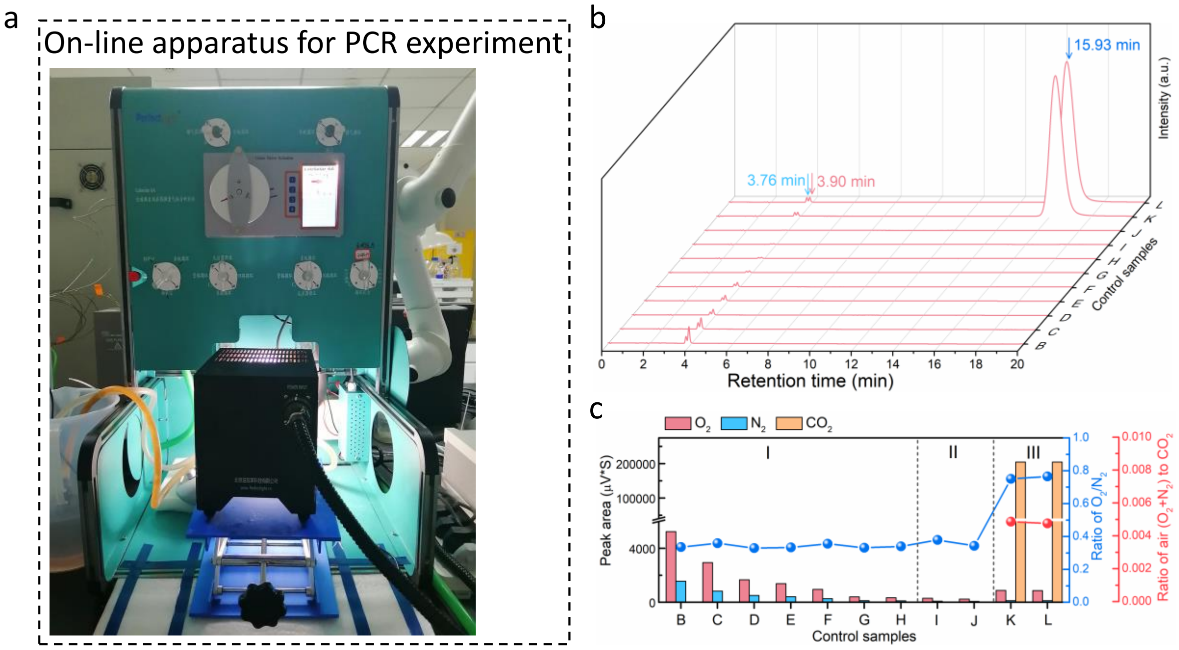

Supplementary Figure 26. (a) Photographs of the on-line equipment used for pure-water PCR. (b) The GC signals of the detected gases in our photoreactor with or without the attendance of CO_2. The peaks emerged at 3.76, 3.90 and 15.93 min were assigned to O_2, N_2, and CO_2, respectively. Control samples were set as the following patterns (B: before the vacuum treatment; C: vacuum treatment for 5 min; D: vacuum treatment for 10 min; E: vacuum treatment for 15 min; F: vacuum treatment for 20 min; G: vacuum treatment for 25 min; H: vacuum treatment for 30 min; I: holding the vacuum for 60 min; J: holding the vacuum for 120 min; K: bubbling the high-purity CO_2 gas through the solution for 60 min; L: holding the pressure of high-purity CO_2 gas in the photoreactor for another 180 min). (c) The variation of signal intensity of detected gases, ratio of O_2 to N_2 and air (O_2 + N_2) to bubbled CO_2.

In order to check the effect of adventitious carbon on CO_2 reduction products, we monitored the GC signals variation of detectable gases in our photoreactor during the whole reaction process because the permeation of trace gas into the photoreactor would result in the sharp variation of GC signals. The whole operating process consisted of three sections:

(i) Vacuum treatment (from B to H)**:** Before the vacuum treatment (B), the peaks of air (O_2 and N_2) emerged simultaneously and no peak of CO_2 could be found, whose volume fraction in air is less than 0.03% (Supplementary Figure 26b). The ratio of N_2 and O_2 in air is 3.51:1, corresponding to their theoretical ratio in air (Supplementary Figure 26c). After the vacuum treatment for 5 min (C), we noticed that the peaks intensity of O_2 and N_2 decreased obviously while the ratio of O_2 to N_2 almost remains unchanged. With the prolonged vacuum treatment time (from D to G), the peaks intensity of O_2 and N_2 decreased persistently while the ratio of O_2 to N_2 is still close to their theoretical ratio in air. No obvious GC signals of O_2 and N_2 could be observed with the further increase of vacuum time (H), suggesting that the residual air in the photoreactor was almost completely removed.

(ii) Holding vacuum (from I to J): In order to check the air impermeability of our measurement setup for a long time, we hold the vacuum for another two hours (I and J). Excitingly, the peaks intensity of O_2 and N_2 still kept unchanged and the ratio of O_2 to N_2 is constant, manifesting the superior air impermeability of our measurement setup.

(iii) Bubbling CO_2 (from K to L): With the bubbling of high-purity CO_2 gas through the solution for 60 min, a prominent peak of CO_2 emerged at 15.93 min (K). Although the peaks of O_2 and N_2 increased slightly, the ratio of air (O_2 and N_2) to CO_2 is less than 0.004:1, suggesting that the bubbling of high-purity CO_2 gas could not bring air into the photoreactor. After holding the pressure of high-purity CO_2 gas in the photoreactor for another 180 min (L), the peaks intensity of detectable gases (O_2, N_2 and CO_2) and ratio of air (O_2 and N_2) to CO_2 still remained unchanged, excluding the possibility that exotic gases permeated into the measurement setup.

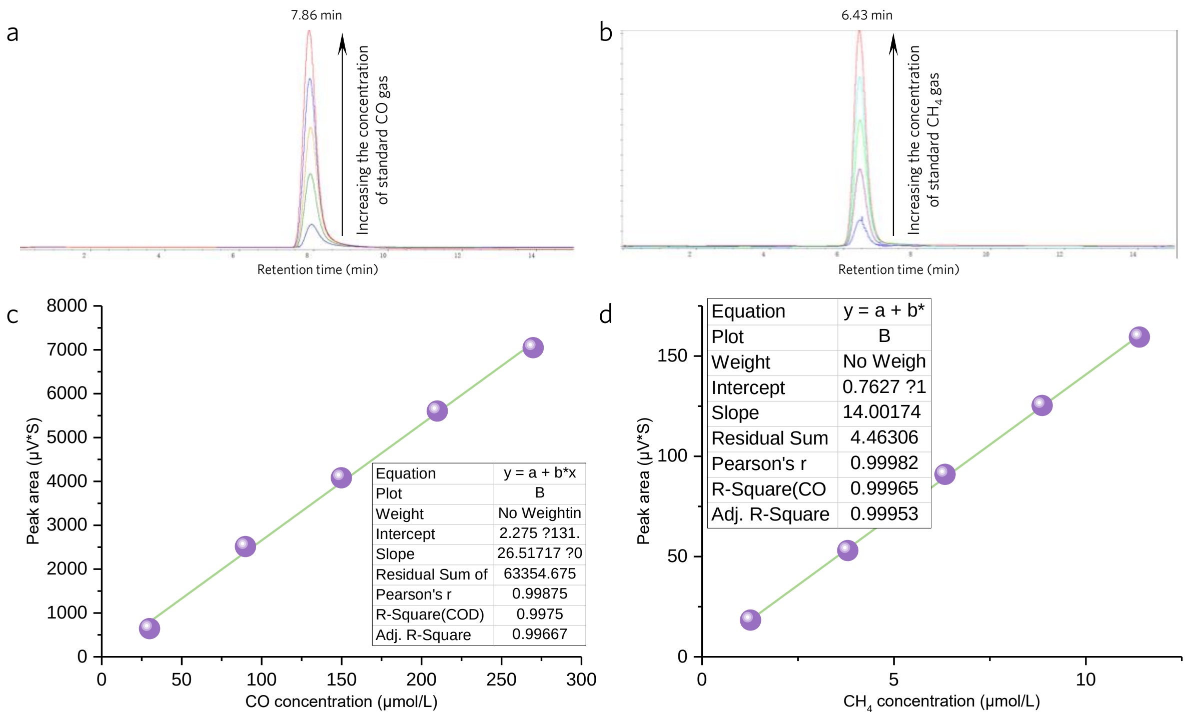

Supplementary Figure 27. (a,b) The GC signals of standard CO (a) and CH_4 (b) with different concentrations. (c,d) The standard curves showing the correlation of CO (c) and CH_4 (d) concentrations with the areas of the GC peaks. A flame ionization detector (FID) was mainly used to quantify the concentrations of CO and CH_4.

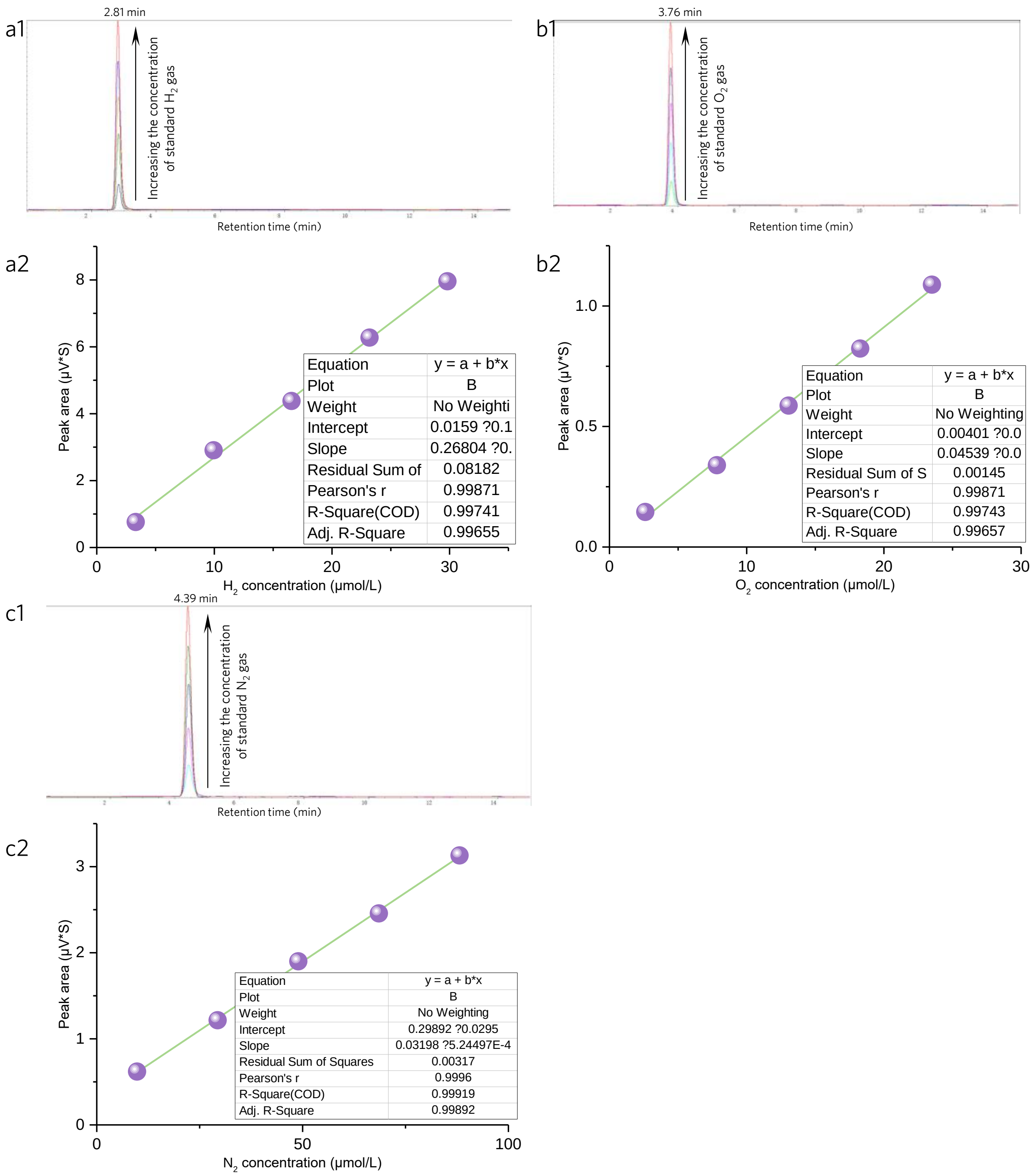

Supplementary Figure 28. (a1,b1,c1) The GC signals of standard H_2 (a1), O_2 (b1), and N_2 (c1) with different concentrations. (c,d) The standard curves showing the correlation of H_2 (a2), O_2 (b2), and N_2 (c2) concentrations with the areas of the GC peaks. A thermal conductivity detector (TCD) was mainly used to quantify the concentrations of H_2, O_2, and N_2.

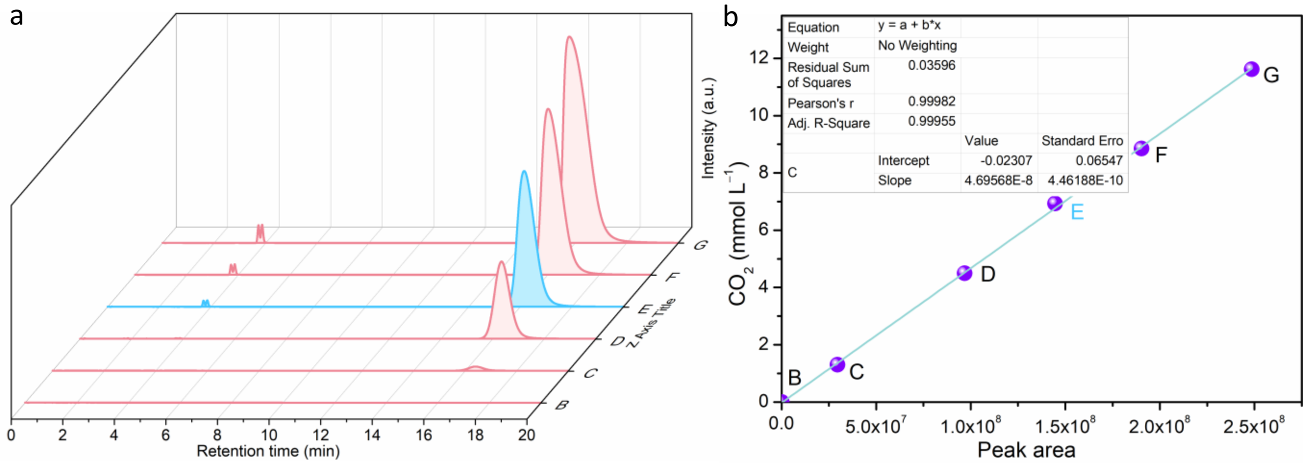

Supplementary Figure 29. (a) The GC signals of high-purity CO_2 with different concentrations; (b) The standard curves showing the correlation of CO_2 concentration with the areas of the GC peaks. B–D and F–G are the standard CO_2 gas with different concentration. E is the purged CO_2 concentration during our photocatalytic CO_2 reduction process.

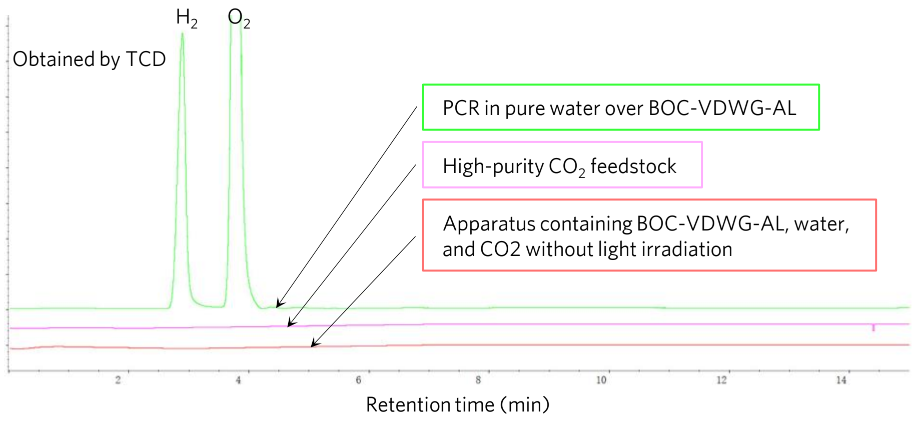

Supplementary Figure 30. The GC signals of two control samples. The first sample was taken from high-purity CO_2 feedstock, while the second was obtained from the apparatus containing BOC-VDWG-AL, water, and CO_2 without light irradiation. The GC signal of PCR in pure water over BOC-VDWG-AL was used as a reference.

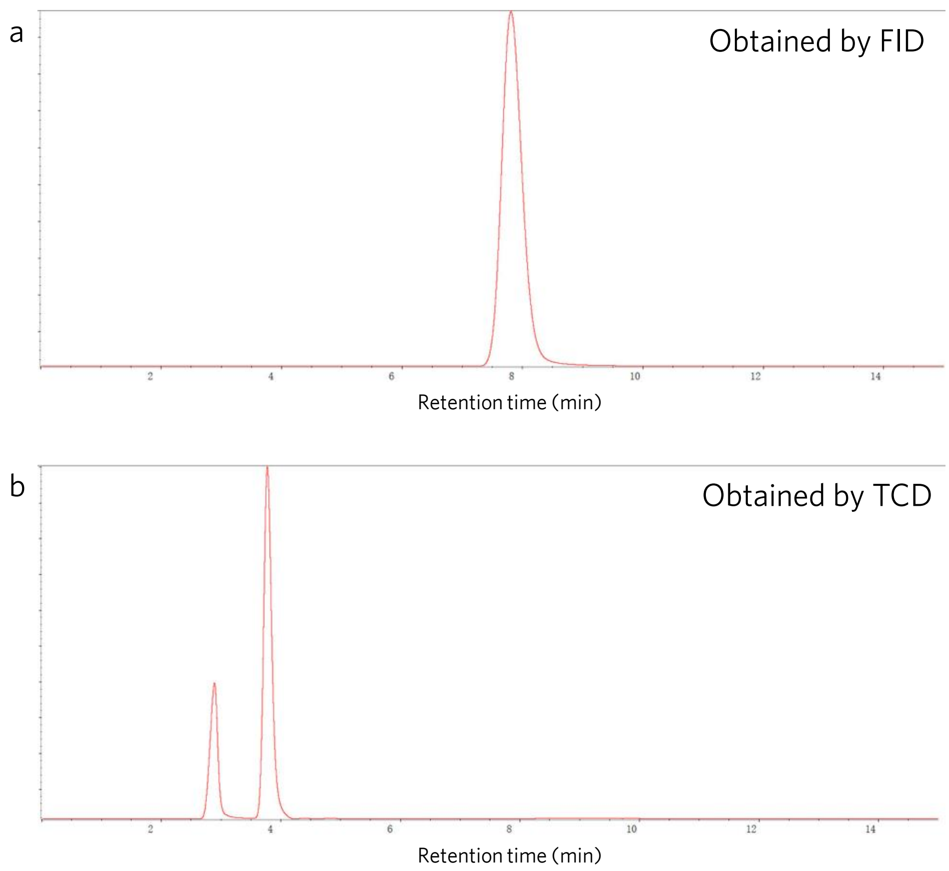

Supplementary Figure 31. The GC signals of the gases generated from PCR in pure water over BOC-VDWG-AL. (a) The pattern obtained from FID. Only CO at 7.86 min was detected. (b) The pattern obtained from TCD. The peak of H_2 and O_2 emerged at 2.81 and 3.76 min, respectively.

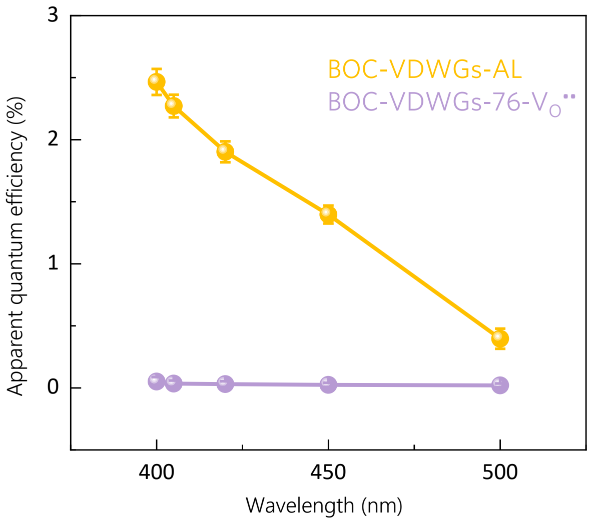

Supplementary Figure 32. The PCR quantum yield as a function of the irradiation wavelength of BOC-VDWGs-AL and BOC-VDWGs-76-$V_O^{••}$. The error bars derived from triplicate experiments.

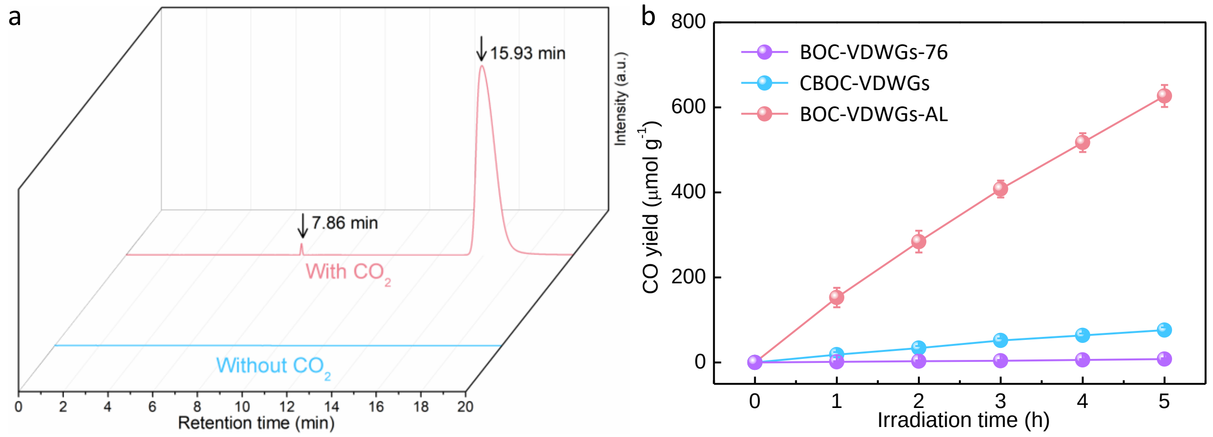

Supplementary Figure 33. (a) The GC signals of the gases generated from PCR in pure water over BOC-VDWG-AL with or without the attendance of CO_2. The peak of CO and CO_2 emerged at 7.86 and 15.93 min, respectively. (b) Comparison of CO evolving rate of BOC-VDWGs-AL, CBOC-VDWGs, and BOC-VDWGs-76, under AM1.5G simulated sunlight. The error bars derived from triplicate experiments.

An obvious signal of CO emerged under AM1.5G simulated sunlight irradiation in the presence of CO_2. However, no any peak could be detected in the absence of CO_2 despite BOC–VDWGs–AL was irradiated under AM1.5G simulated sunlight for 2 h, manifesting that BOC–VDWGs–AL could maintain superior stability and no any decomposition product was detected under UV light irradiation.

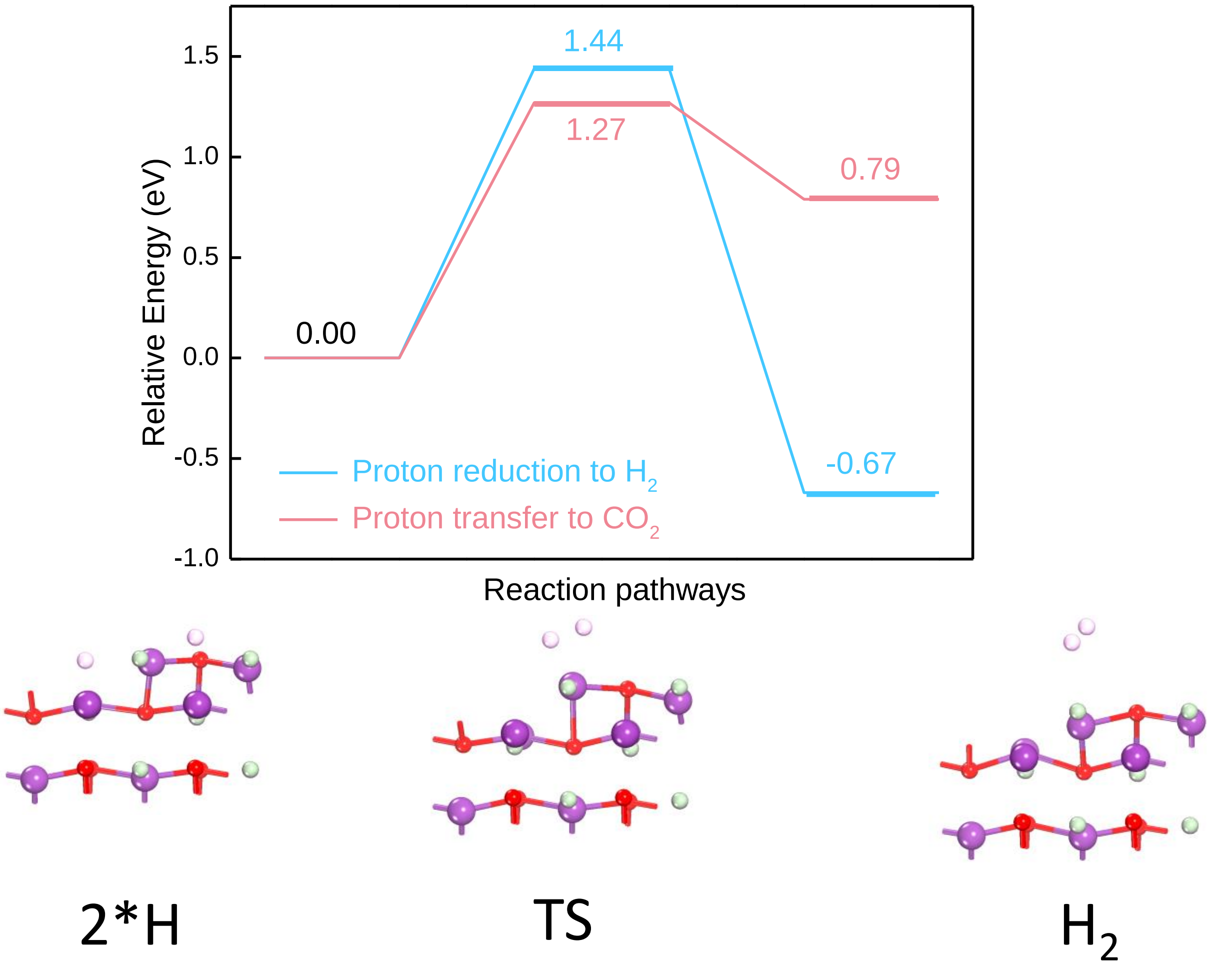

Supplementary Figure 34. Gibbs free energy diagrams of proton transfer to CO_2 versus proton dimerization to H_2 over VDWG-Bi-$V_O^{\bullet\bullet}$-Bi. As for VDWG-Bi-$V_O^{\bullet\bullet}$-Bi, the H_2 formation is exothermic by 0.67 with an activation energy barrier (E_a) of 1.44 eV, while the proton transfer to an O atom of the absorbed CO_2 is endothermic by 0.79 eV with E_a = 1.27 eV. Therefore, on VDWG-Bi-$V_O^{\bullet\bullet}$-Bi, the proton tends to transfer to the absorbed CO_2 because of the lower E_a.

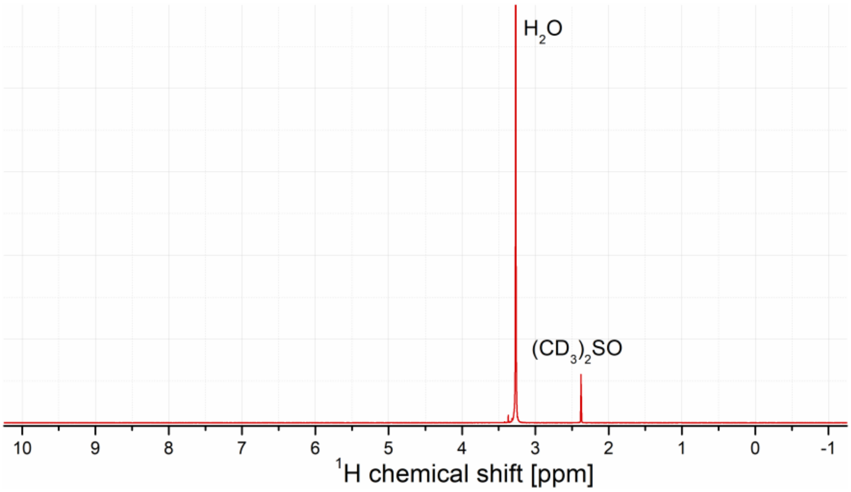

Supplementary Figure 35. Representative ^{1}HNMR spectrum of the liquid after 8h CO_2 photocatalysis for the BOC-VDWGs-AL. DMSO is used as an internal standard. The peaks at 3.35 ppm and 2.50 ppm were assigned to H_2O and $(CD_3)_2SO$, respectively. This result clearly showed that no liquid products were detected by the NMR measurement.

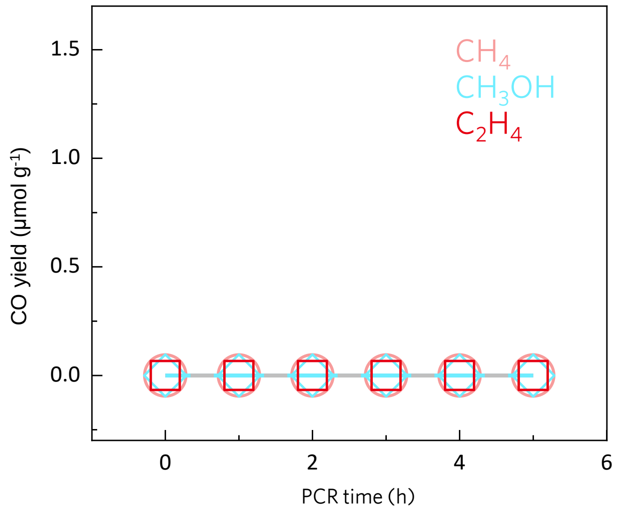

Supplementary Figure 36. The yield of methane, methanol, and ethylene as a function of the time of PCR over BOC-VDWGs-AL.

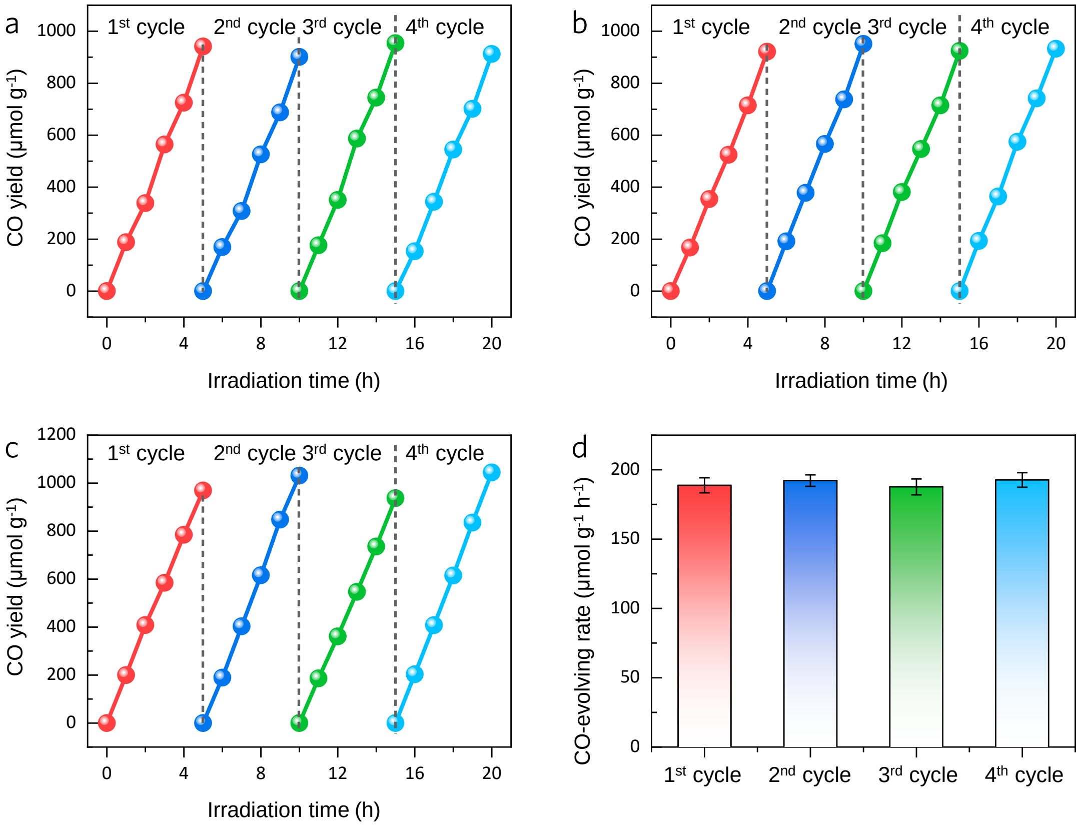

Supplementary Figure 37. (a-c) Repeating the cycling test for three times. (d) The averaged pure-water PCR rates of each cycling test. The error bars derived from triplicate experiments. The used photocatalyst is BOC-VDWGs-AL. After each cycling test, the reaction cell was vacuumized with the water renewed completely, while the catalyst was not subject to any treatments.

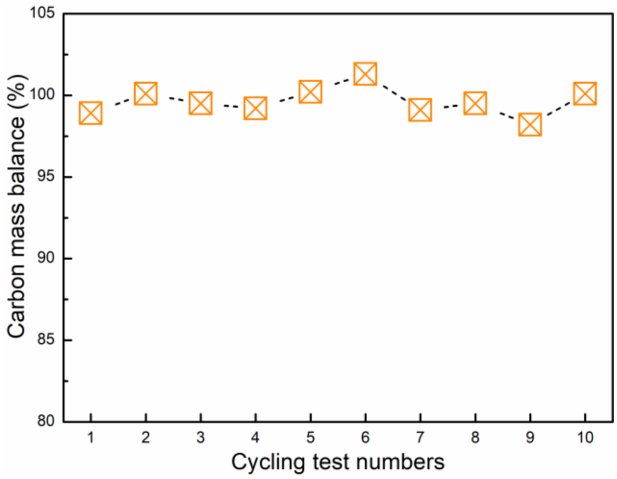

Supplementary Figure 38. The carbon mass balance of CO_2 photoreduction over BOC-VDWGs-AL.

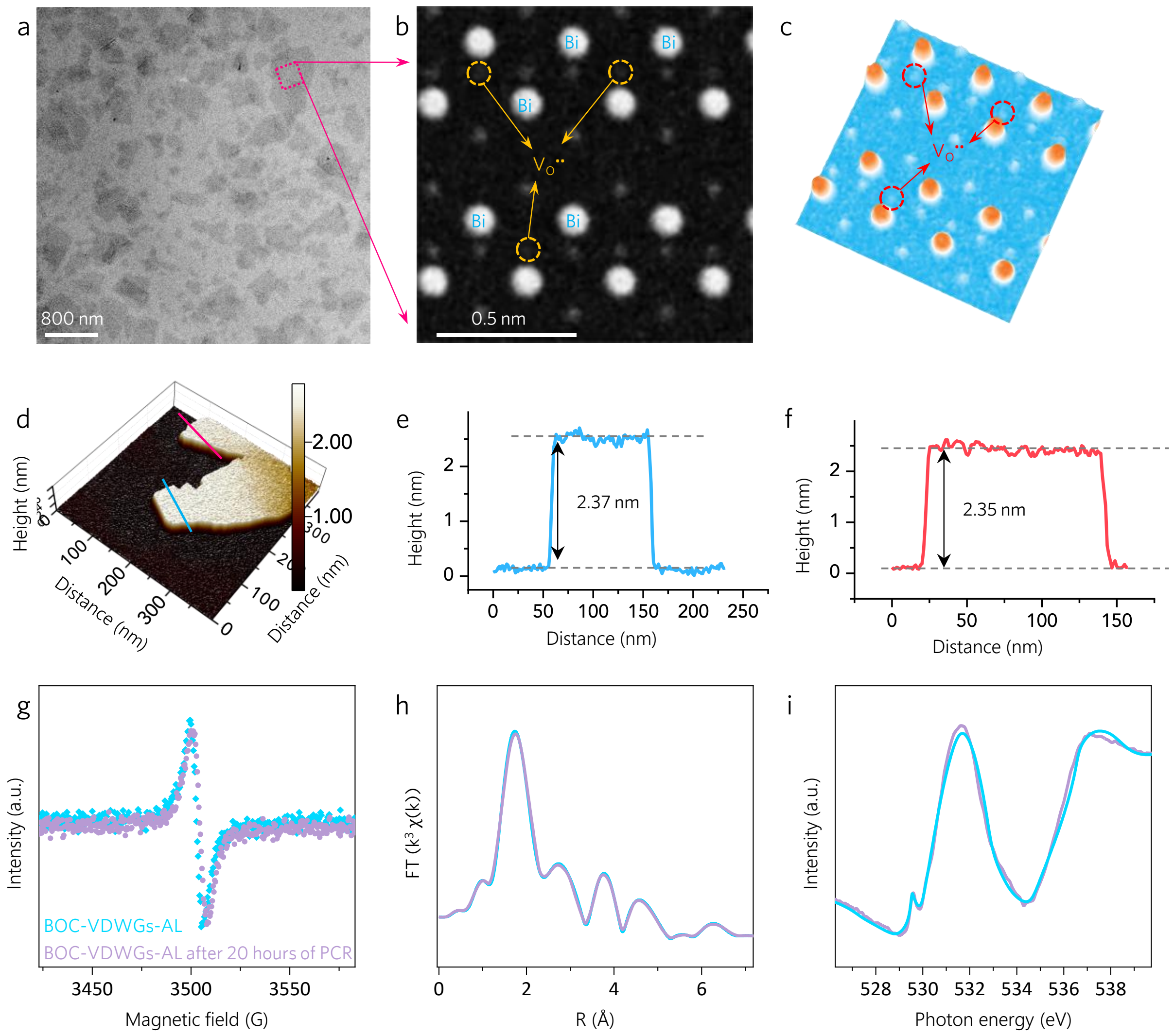

Supplementary Figure 39. Characterizations of BOC-VDWGs-AL after 20 hours of pure-water PCR, confirming the stability. (a) TEM image, (b) atom-resolved HAADF-STEM image, (c) 3D topographic color-coded intensity image (converted from **b**), (d) AFM image, (e,f) intensity profiles (along lines in panel **d**), (g) EPR, (h) Bi L_3-edge EXAFS, and (i) O K-edge XANES of BOC-VDWGs-AL after 20 hours of PCR.

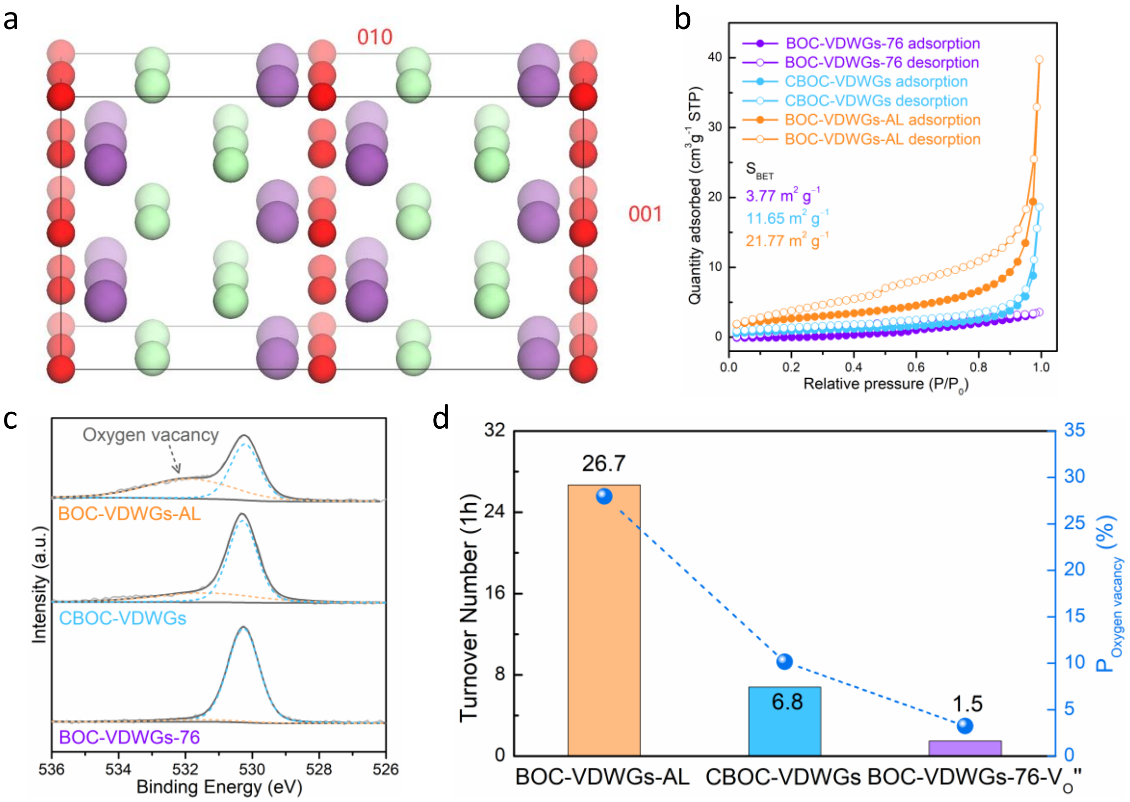

Supplementary Figure 40. (a) Unit cell of (010)-BiOCl with lattice parameters of 3.891 Å *3.891 Å *7.369 Å, and the red, green and purple balls represent O, Cl and Bi, respectively. The (010) surface bears a surface area of 2.867*10^{-19} m^2 ($S_{unit\ cell}$ = 28.67 $Å^2$ = 0.2867 nm^2 =2.867*10^{-19} m^2) and 4 surface oxygen atoms ($N_{O(per\ unit\ cell)}$= 1 + 1(4) + 2(4) = $4_{(per\ unit\ cell)}$). (b) BET plots, (c) High-resolution O 1*s* XPS spectra and (d) the turnover number (TON) of BOC-VDWGs-76-$V_O^{\bullet\bullet}$, CBOC-VDWGs and BOC-VDWGs-AL.

According to the configuration in Supplementary Figure 40a, we noticed that the $S_{unit\ cell}$ of (010)-BiOCl is 2.867*10^{-19} m^2 while the N_O of (010)-BiOCl per unit cell is 4. The S_{BET} of BOC-VDWGs-76-$V_O^{\bullet\bullet}$, CBOC-VDWGs and BOC-VDWGs-AL are 3.77, 11.65 and 21.77 m^2 g^{-1}, respectively (Supplementary Figure 40b). As displayed in the high-resolution O 1*s* XPS spectra, the $P_{Oxygen\ vacancy}$ of BOC-VDWGs-76-$V_O^{\bullet\bullet}$, CBOC-VDWGs and BOC-VDWGs-AL is 3.24%, 10.15% and 27.95%, respectively (Supplementary Figure 40c). Therefore, the TON of CO product for the BOC-VDWGs-AL reached 26.7, surpassing those of CBOC-VDWGs (6.8) and BOC-VDWGs-76-$V_O^{\bullet\bullet}$ (1.5) (Supplementary Figure 40d). More importantly, the calculated TON of BOC-VDWGs-76-$V_O^{\bullet\bullet}$, CBOC-VDWGs and BOC-VDWGs-AL were also greater than one, manifesting that an actual catalytic reaction proceeded.

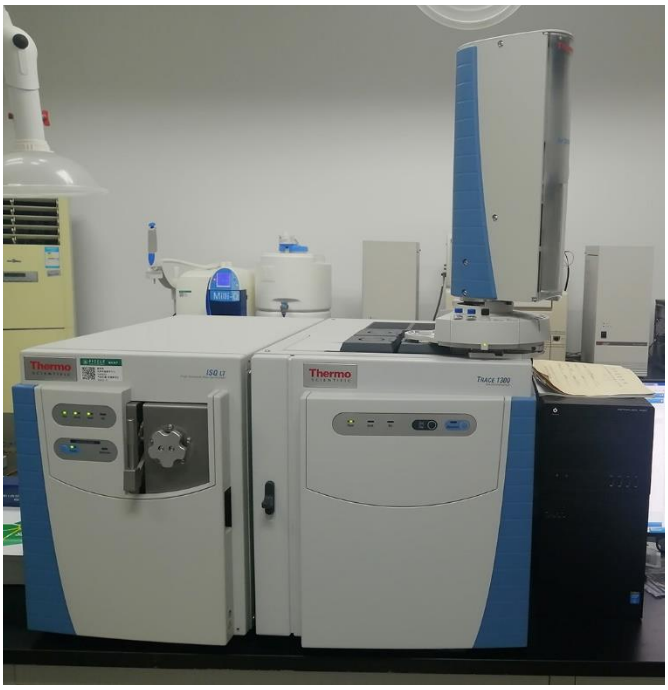

Supplementary Figure 41. Photograph of GC-mass spectrometry used for identifying ^{13}CO.

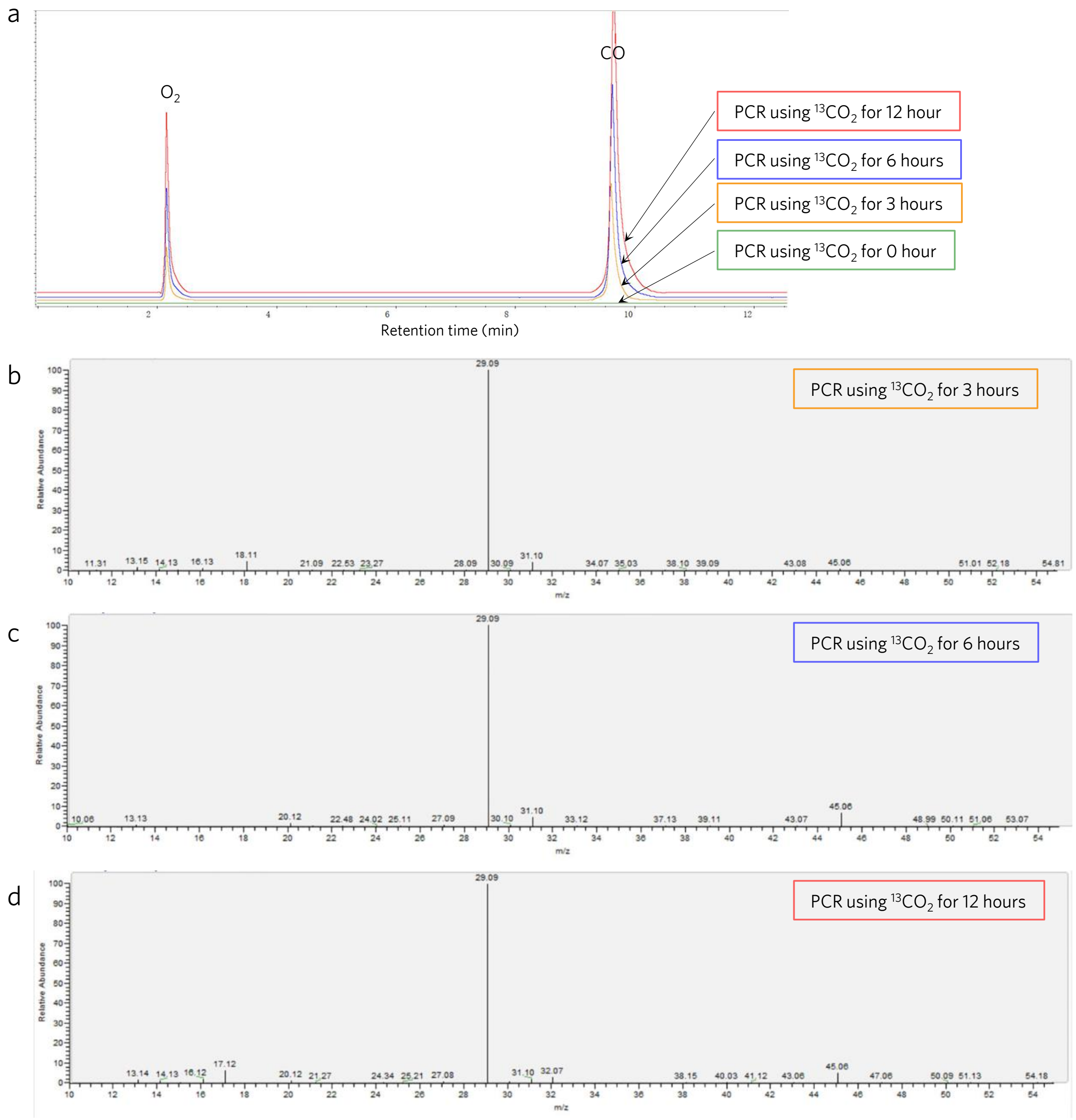

Supplementary Figure 42. The GC-MS data of the product obtained from the PCR experiment driven by BOC-VDWGs-AL using $^{13}CO_2$ as the source gas. (a) The GC patterns of O_2 and ^{13}CO generated during PCR over BOC-VDWGs-AL using $^{13}CO_2$ for 0, 3, 6, and 12 hours. (b-d) The corresponding MS patterns.

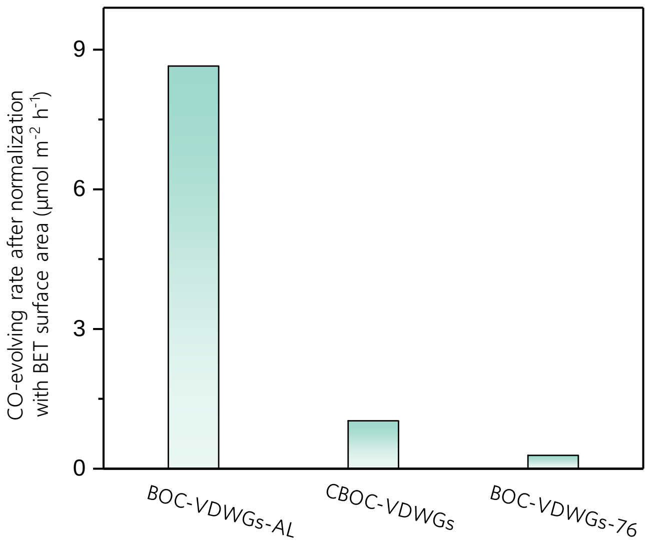

Supplementary Figure 43. Comparison of the PCR rates of BOC-VDWGs-AL, CBOC-VDWGs and BOC-VDWGs-76 after normalization with BET surface area.

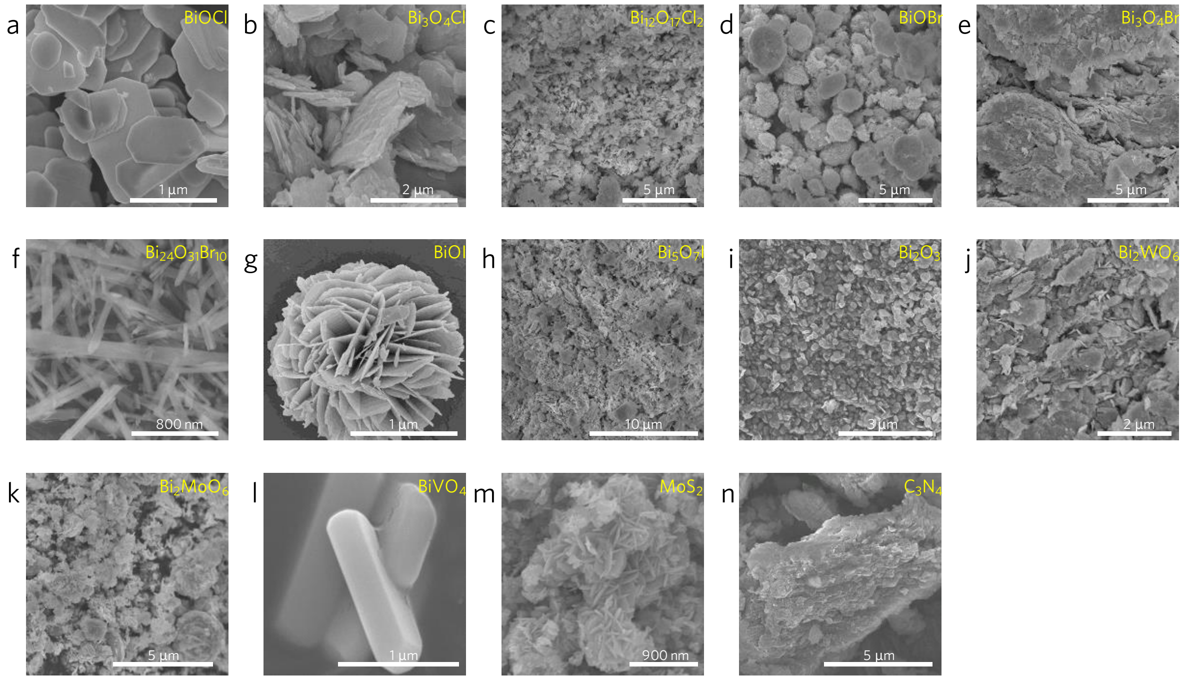

Supplementary Figure 44. SEM images of some representative layered photocatalysts as control samples.

BiOCl was synthesized by hydrothermal treatment of the mixed solution of $Bi(NO_3)_3$•$5H_2O$ and KCl at 160 °C for 18 hours.

Bi_3O_4Cl was synthesized by hydrothermal treatment of the mixed solution (pH = 10.6) of water and NH_3•H_2O containing $Bi(NO_3)_3$•$5H_2O$ and KCl at 180 °C for 24 hours.

$Bi_{12}O_{17}Cl_2$ was synthesized by wet-chemistry treatment of the mixed solution (pH = 13.4) of water and ethanol containing $BiCl_3$ and NaOH, followed by heating at 450 °C for 2 hours.

BiOBr was synthesized by solvothermal treatment of ethylene glycol (pH = 6) containing $Bi(NO_3)_3$•$5H_2O$, and KBr at 160 °C for 18 hours.

Bi_3O_4Br was synthesized by hydrothermal treatment of the mixed solution (pH = 10.9) of water and NH_3•H_2O containing $Bi(NO_3)_3$•$5H_2O$ and KBr at 180 °C for 24 hours.

$Bi_{24}O_{31}Br_{10}$ was synthesized by wet-chemistry treatment of the mixed solution (pH = 12.3) of water and ethanol containing $BiBr_3$ and NaOH, followed by heating at 350 °C for 2 hours.

BiOI was synthesized by hydrothermal treatment of the mixed solution (pH = 6) of water and ethanol containing $Bi(NO_3)_3$•$5H_2O$ and KI at 120 °C for 12 hours.

Bi_5O_7I was synthesized by wet-chemistry treatment of the mixed solution (pH = 11.5) of water and ethanol containing BiI_3 and NaOH, followed by heating at 410 °C for 2 hours.

Bi_2O_3 was synthesized by wet-chemistry treatment of the mixed solution (pH = 14) of water and ethanol containing $BiCl_3$ and NaOH, followed by heating at 800 °C for 2 hours.

Bi_2WO_6 was synthesized by hydrothermal treatment of the mixed solution of $Bi(NO_3)_3$•$5H_2O$ and Na_2WO_4•$2H_2O$ at 180 °C for 24 hours.

Bi_2MoO_6 was synthesized by solvothermal treatment of ethylene glycol containing $Bi(NO_3)_3$•$5H_2O$, Na_2MoO_4•$2H_2O$, and ethylenediamine at 160 °C for 24 hours.

$BiVO_4$ was synthesized by hydrothermal treatment of the mixed solution (pH = 2) of $Bi(NO_3)_3$•$5H_2O$, NH_4VO_3, and nitric acid at 200 °C for 24 hours.

MoS_2 was synthesized by hydrothermal treatment of the mixed solution of $(NH_4)_6Mo_7O_{24}\cdot 4H_2O$ and thiourea at 220 °C for 36 hours.

C_3N_4 was synthesized by thermal treatment of melamine at 550 °C for 4 hours.

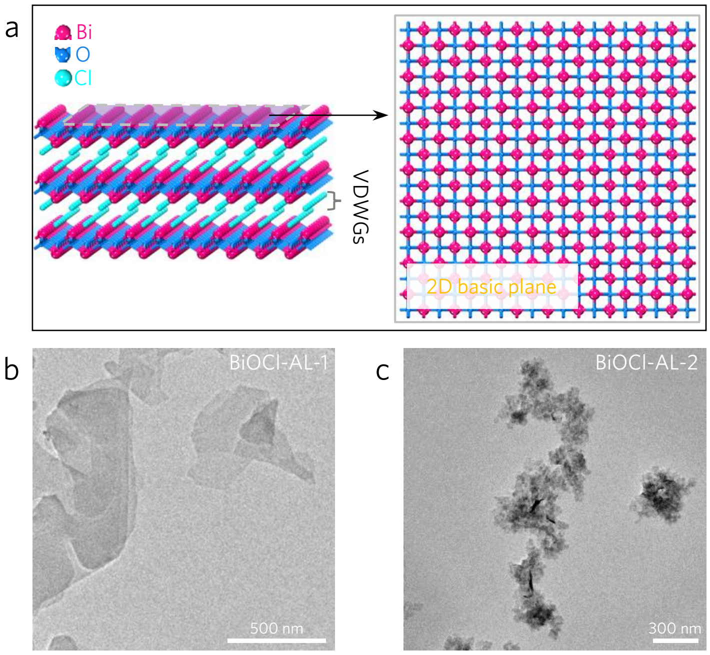

Supplementary Figure 45. Characterizations of ultrathin BiOCl nanosheets with VDWGs on their lateral facets as control samples. (a) Schematic illustration of the crystalline structure of BiOCl-AL-1 and BiOCl-AL-2. (b) TEM images of BiOCl-AL-1 and BiOCl-AL-2.

BiOCl-AL-1 was synthesized by ultrasonication (driven by an ultrusonic cell disrupter system) of BiOCl for 12 hours.

BiOCl-AL-2 was synthesized by hydrothermal treatment of the mixed solution of mannitol, $Bi(NO_3)_3 \cdot 5H_2O$ and hexadecyltrimethylammonium chloride at 160 °C for 6 hours.

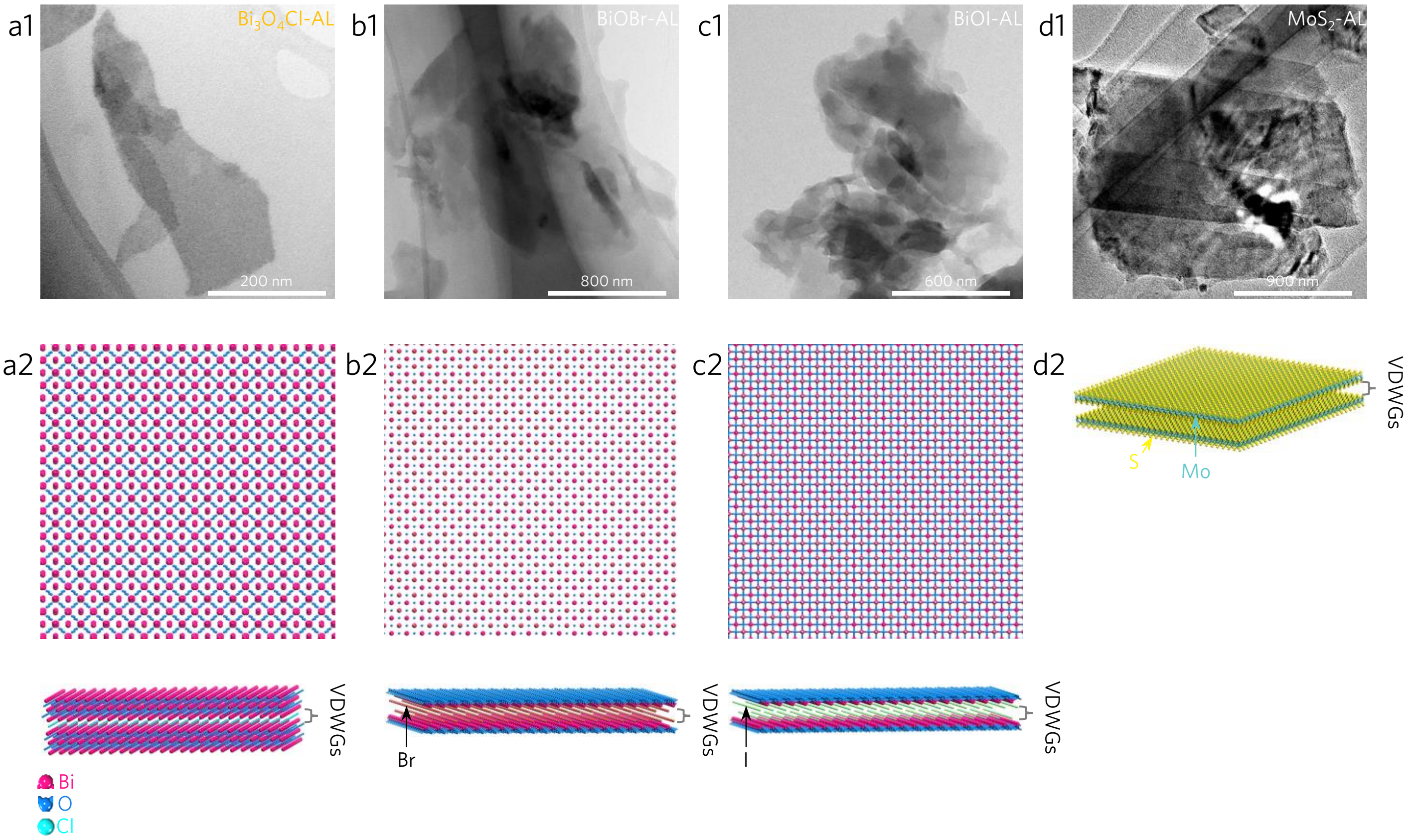

Supplementary Figure 46. Characterizations of ultrathin Bi_3O_4Cl, BiOBr, BiOI, and MoS_2 nanosheets with VDWGs on their lateral facets as control samples. (a1-d1) TEM images of Bi_3O_4Cl-AL, BiOBr-AL, BiOI-AL, and MoS_2-AL. (a2-d2) Schematic illustration of the crystalline structure of Bi_3O_4Cl-AL, BiOBr-AL, BiOI-AL, and MoS_2-AL.

Bi_3O_4Cl-AL, BiOBr-AL, BiOI-AL, and MoS_2-AL were synthesized by ultrasonication (driven by an ultrusonic cell disrupter system) of Bi_3O_4Cl, BiOBr, BiOI, and MoS_2 bulk crystals for 12 hours, respectively.

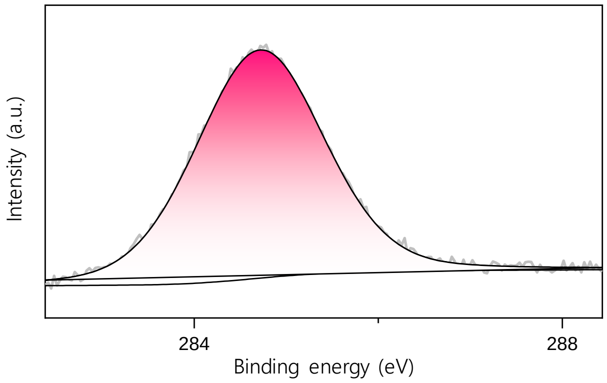

Supplementary Figure 47. Additional characterizations to confirm that BOC-VDWGs-AL is carbon-free. C 1*s* XPS of BOC-VDWGs-AL.

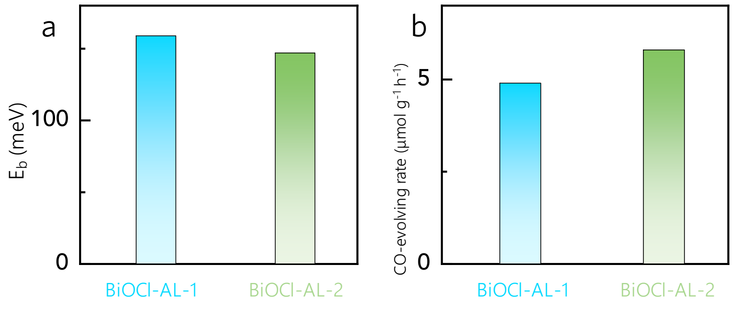

Supplementary Figure 48. (a) E_b, and (b) CO-evolving rates of BiOCl-AL-1 and BiOCl-AL-2.

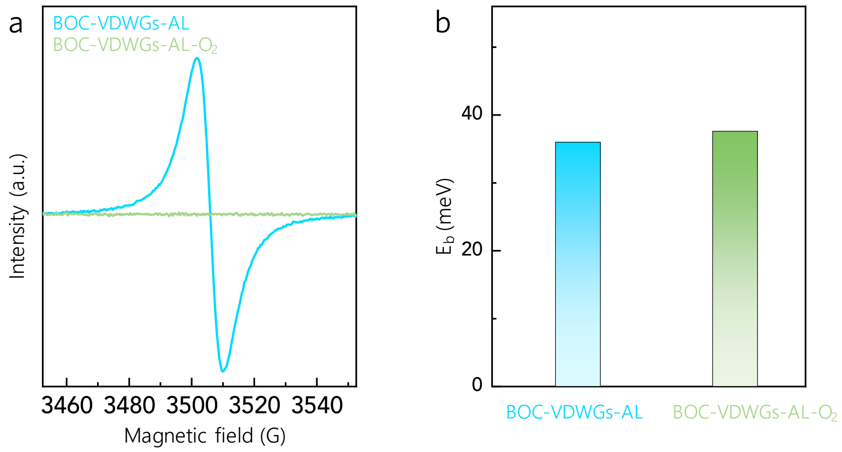

Supplementary Figure 49. Characterizations of BOC-VDWGs-AL-O_2, suggesting its oxygen-vacancy-free nature. (a) EPR and (b) E_b of BOC-VDWGs-AL-O_2 and BOC-VDWGs-AL. BOC-VDWGs-AL-O_2 was synthesized by calcination of BOC-VDWGs-AL in O_2 at 300 ^oC for 12 hours.

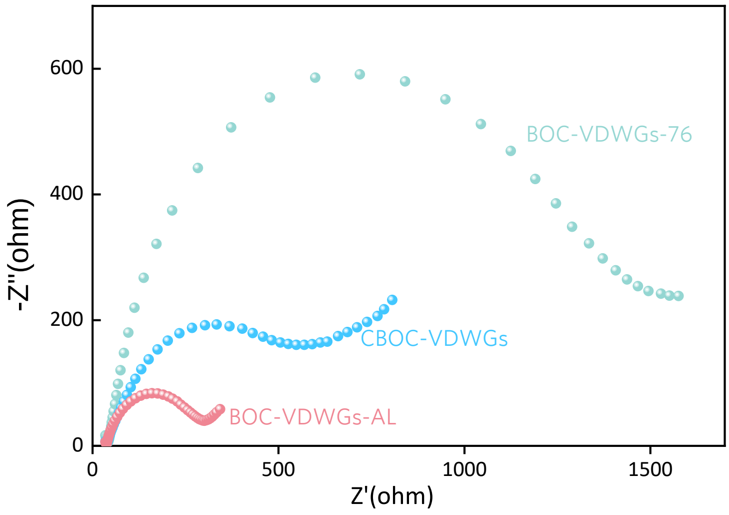

Supplementary Figure 50. Characterization of the impact of the VDWG exposure percentage on the charge transfer process. Electrochemical impedance spectroscopy of BOC-VDWGs-AL, CBOC-VDWGs, and BOC-VDWGs-76. Nyquist plots at −0.2 V in a solution containing 0.1 M KCl, 0.01 M $K_3Fe(CN)_6$ and 0.01 M $K_4Fe(CN)_6$.

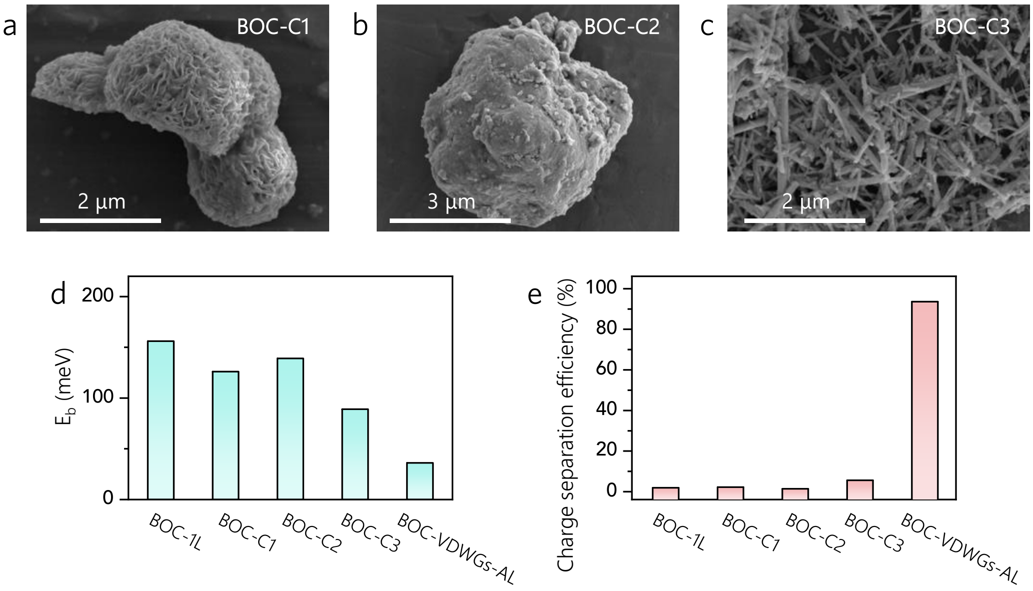

Supplementary Figure 51. (a-c) SEM images, (d) E_b, and (e) η_{bulk} of BiOCl of microsphere (BOC-C1), microparticle (BOC-C2), and nanowire (BOC-C3).

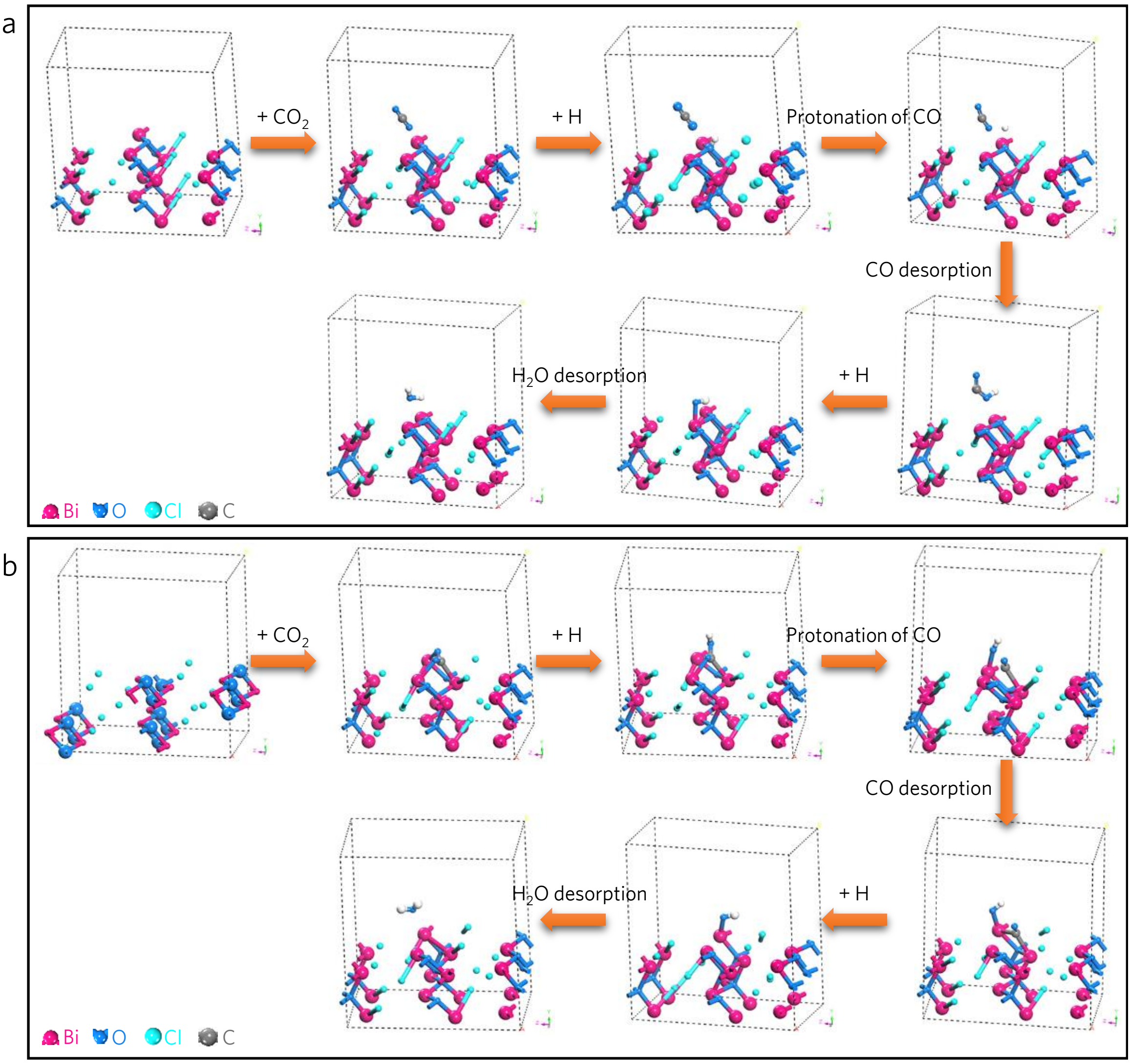

Supplementary Figure 52. Optimized geometric structures of intermediates generated during CO_2-to-CO catalysis over VDWG-Bi-O-Bi (a) and VDWG-Bi-$V_O^{••}$-Bi (b).

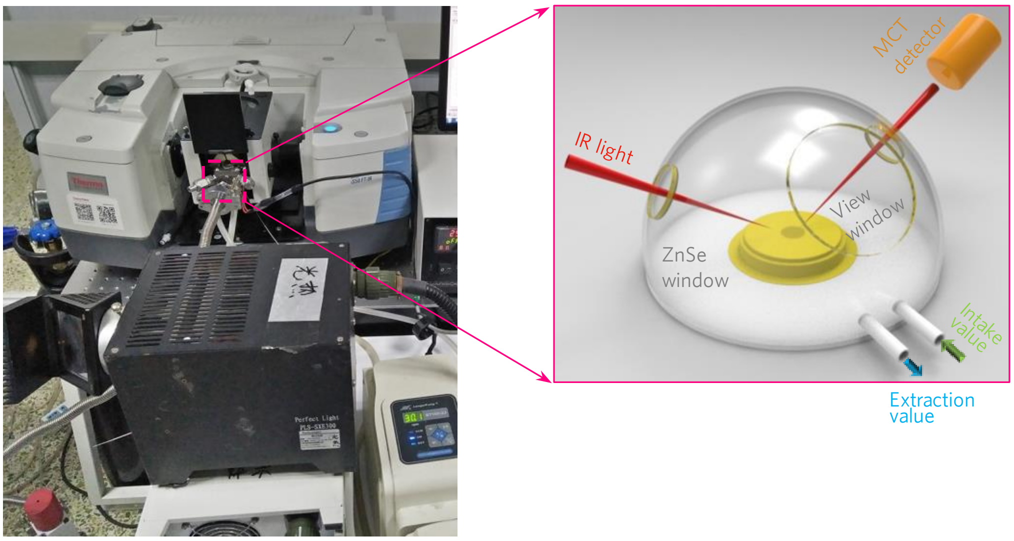

Supplementary Figure 53. Photograph of the in situ FTIR used for detecting intermediates generated during PCR.

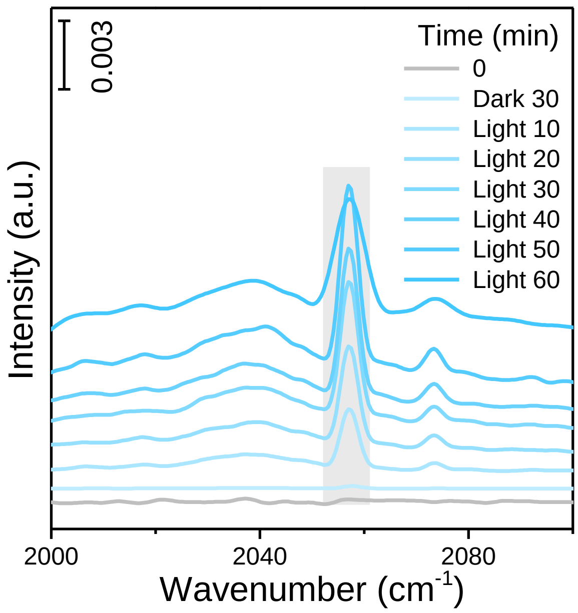

Supplementary Figure 54. Characterization of the produced CO. In situ FTIR of CO product generated from BOC-VDWGs-AL as a function of PCR time. The peak at 2058 cm^{-1} is assigned to the generated CO product.

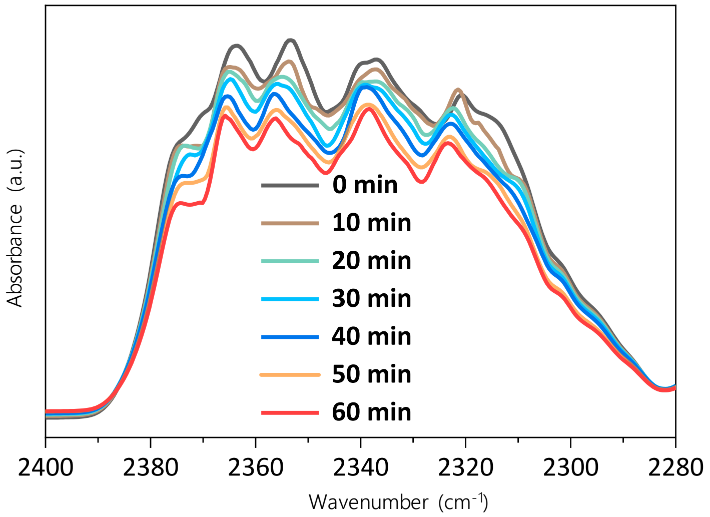

Supplementary Figure 55. Characterization of the variation of CO_2 concentration. *In situ* FTIR of CO_2 as a function of PCR time. The peak at 2300-2380 cm^{-1} is assigned to CO_2. The CO_2-to-CO photocatalysis consumes CO_2, resulting in a continuous reduction in CO_2 concentration.

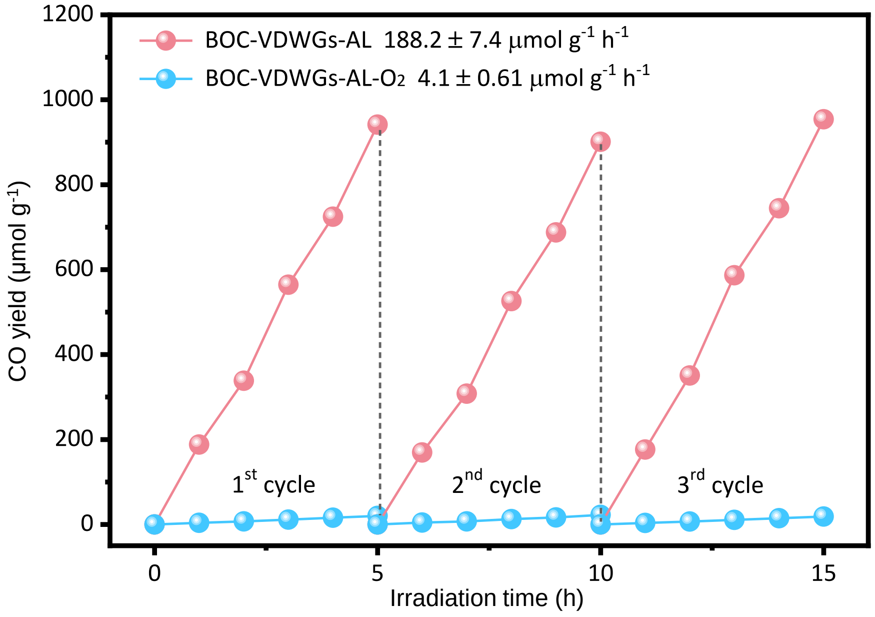

Supplementary Figure 56. Comparison of the PCR rate of BOC-VDWGs-AL and BOC-VDWGs-AL-O_2 under visible light.

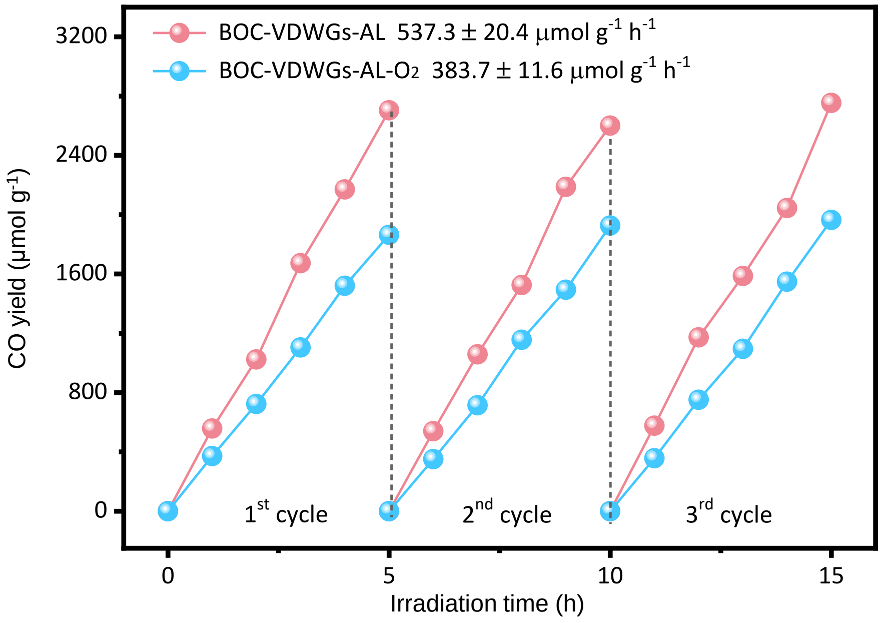

Supplementary Figure 57. Comparison of the PCR rate of BOC-VDWGs-AL and BOC-VDWGs-AL-O_2 under ultraviolet light.

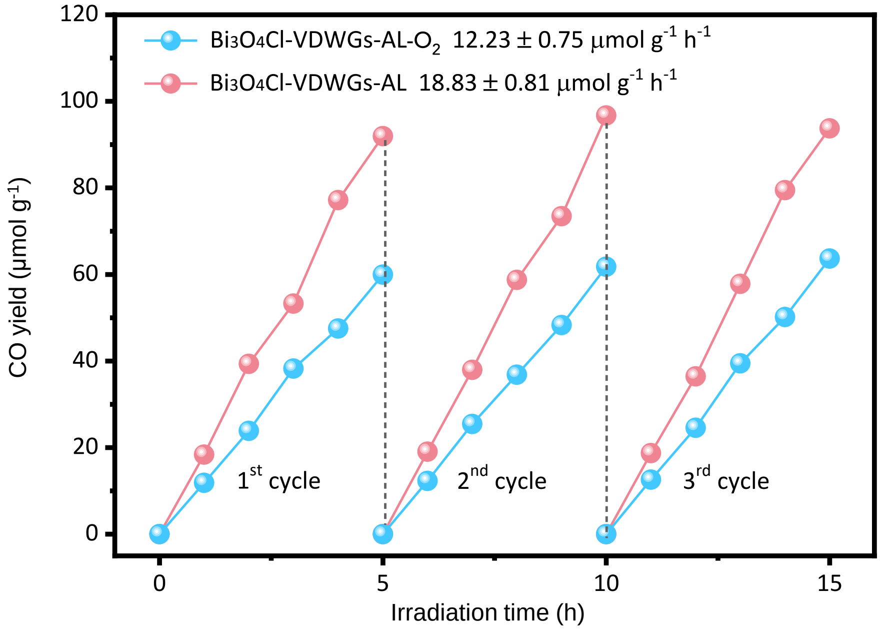

Supplementary Figure 58. Comparison of the PCR rate of Bi_3O_4Cl-VDWGs-AL and Bi_3O_4Cl-VDWGs-AL-O_2 under 420 nm monochromatic light.

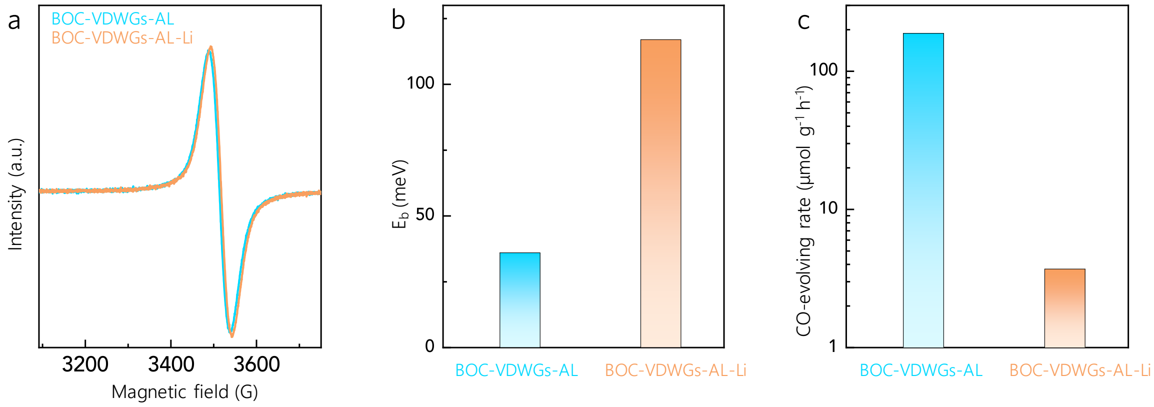

Supplementary Figure 59. Characterizations of BOC-VDWGs-AL-Li. (a) EPR, (b) E_b, and (c) CO-evolving rates of BOC-VDWGs-AL-Li and BOC-VDWGs-AL.

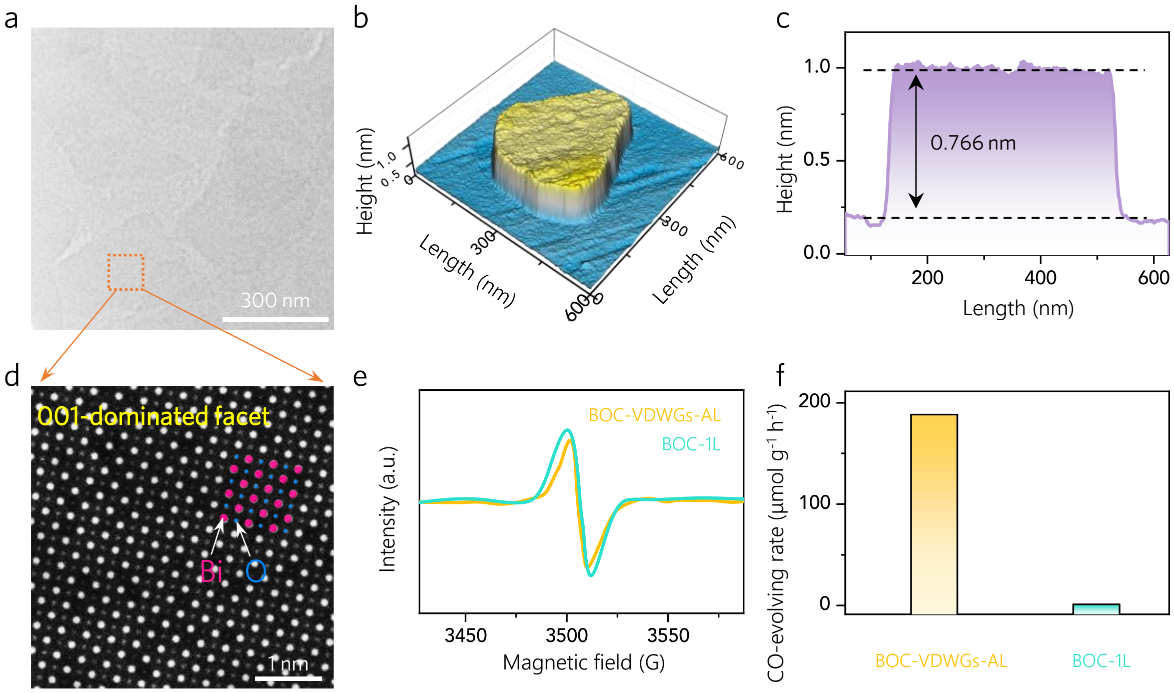

Supplementary Figure 60. Characterization and PCR performance of (001)-dominated single-layered BiOCl nanosheet (BOC-1L). (a) TEM image, (b) AFM image, (c) intensity profile, (d) HAADF-STEM image, (e) EPR, and CO-evolving rate of BOC-1L.

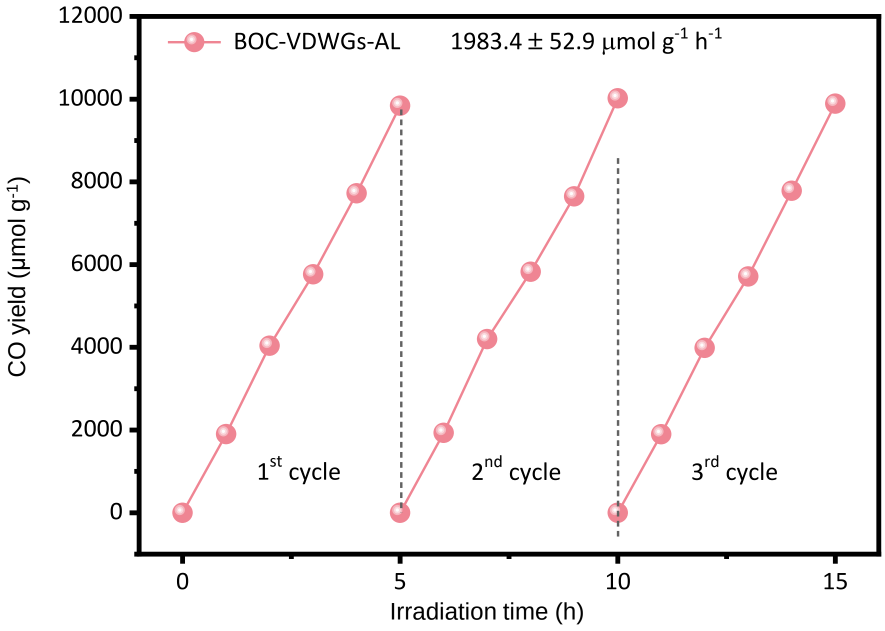

Supplementary Figure 61. PCR performance under visible light of BOC-VDWGs-AL in the presence of Ag (as cocatalyst) and triethanolamine (as hole scavenger).

Supplementary Tables

Supplementary Table 1. Positron lifetime parameters of BOC-VDWGs-AL and BOC-VDWGs-76.

Sample	τ_1 (ps)	τ_2 (ps)	τ_3 (ns)	I_1 (%)	I_2 (%)	I_3 (%)
BOC-VDWGs-AL	192	396	2.36	86.3	9.8	3.9
BOC-VDWGs-76	196	393	2.39	39.9	57.3	2.8

The theoretical positron lifetimes in our cases were firstly calculated based on the atomic superposition (ATSUP) method [*J. Phys. F 1983, 13, 333; J. Phys.: Condens. Matter 2007, 19, 176222*]. Considering that the non-self-consistent superposition of free atom electron density and Coulomb potential in the absence of the positron, the electron density and the positron crystalline Coulomb potential were constructed. The electron-positron enhancement factor was used in the positron lifetime calculations, which was described within the generalized gradient approximation by Barbiellini et al [*Phys. Rev. B 1996, 53, 16201*]. Once the collected PAS data fitted into two or four positron lifetime components, an incorrect program prompt would pop up. Therefore, the measured positron lifetime of BOC-VDWGs-AL was rationally fitted into three positron lifetime components and τ_1 (192 ps) was assigned to the $V_O^{\bullet\bullet}$ based on the theoretically calculated lifetime of positrons. τ_2 (396 ps) and τ_3 (2.36 ns) was assigned to cluster-like vacancies, and interfaces or grain boundaries, highly agreeing with some previously reported results [*J. Am. Chem. Soc. 2013, 135, 10411-10417; J. Mater. Chem. A, 2017, 5, 23453-23459*]. The high proportion (86.3%) of τ_1 for BOC-VDWGs-AL manifests that VDWG-Bi-$V_O^{\bullet\bullet}$-Bi is a dominated defect, distinct from that in BOC-VDWGs-76.

Supplementary Table 2. Comparison of the pure-water, PCR performance of van der Waals gap-rich BiOCl atomic layer with those of some representative photocatalysts without the involvement of co-catalyst, hole scavenger, and organic solvent.

Photocatalyst	Light source	Catalyst dosage	Product	Yield (μmol g^{-1} h^{-1})	CO_2 source	Reference
$BiOIO_3$	300 W Xe (UV-vis)	0.02 g	CO	17.33	H_2SO_4 + $NaHCO_3$	*Adv. Mater.* ***2020***, *32*, *1908350*
Co-Bi_3O_4Br atomic layer	300 W Xe (UV-vis)	0.03 g	CO	107.1	high-purity CO_2	*Nat. Commun.* **2019**, *10*, 2840.
Br-grafted $Bi_2O_2(OH)(NO_3)$ NSs	300 W Xe (UV-vis)	0.02 g	CO	8.12	H_2SO_4 + $NaHCO_3$	*Adv. Mater.* **2019**, 1900546.
UN-$BiOIO_3$	300 W Xe (UV-vis)	0.05 g	CO	5.42	H_2SO_4 + $NaHCO_3$	*Adv. Funct. Mater.* **2018**, 1804284.
$Bi_{12}O_{17}Cl_2$ NTs	300 W Xe (UV-vis)	0.03 g	CO	48.6	high-purity CO_2	*Angew. Chem. Int. Ed.* **2018**, *130*, 1.
BiOBr-OVs Atomic Layers	300 W Xe (>400 nm)	0.10 g	CO	87.4	high-purity CO_2	*Angew. Chem. Int. Ed.* **2018**, *130*, 8855.
$Sr_2Bi_2Nb_2TiO_{12}$-OVs	300 W Xe (UV-vis)	0.10 g	CO	11.7	H_2SO_4 + $NaHCO_3$	*Angew. Chem. Int. Ed.***2019**, *58*, 3880.
$BiOBr_xCl_{1-x}$	Xe (0.2 W cm^{-2})	0.01 g	CO	15.86	high-purity CO_2	*Appl. Catal., B* **2019**, *243*, 734.
BiOCl	500 W Xe (UV-vis)	0.10 g	CO	1.01	high-purity CO_2	*Nano Res.* **2014**, *8*, 821.
BiOBr	Xe (0.2 W cm^{-2})	0.05 g	CO	4.45	H_2SO_4 + $NaHCO_3$	*Catal. Sci. Technol.* **2017**, *7*, 265.
BiOI	300 W Xe	0.05 g	CO	5.18	H_2SO_4 + $NaHCO_3$	*Chem. Eng. J.* **2016**, *291*, 39.
Van Der Waals Gap-Rich BiOCl Atomic Layer	300W Xe (>400 nm)	0.05 g	CO	188.2	high-purity CO_2	This work

Supplementary Table 3. Comparison of the PCR performance of van der Waals gap-rich BiOCl atomic layer with those of some representative photocatalysts with the involvement of co-catalyst, hole scavenger, or organic solvent.

Photocatalyst	Light source	Product	Yield (μmol g^{-1} h^{-1})	Co-catalyst	Hole scavenger	Reaction medium	Reference
g-C_3N_4	300 W Xe lamp (> 400 nm)	CO	17	Co-porphyrin	TEOA	CH_3CN	*Appl. Catal., B* **2017,** *200*, 141.
CdS	300 W Xe lamp (≥ 400 nm)	CO	50.4	Co-ZIF-9	TEOA	CH_3CN + H_2O	*Appl. Catal., B* **2015,** *162*, 494.
MOF-525	300 W Xe lamp (≥ 400 nm)	CO	200.6	Single atom Co	TEOA	CH_3CN	*Angew. Chem. Int. Ed.* **2016,** *55*, 14310.
g-C_3N_4	Xe lamp (≥ 420 nm)	CO	20.8	Co-ZIF-9	TEOA	Bipyridine + CH_3CN + H_2O	*Phys. Chem. Chem. Phys.* **2014,** *16*, 14656.
o-PCN	300 W Xe lamp (> 400 nm)	CO<br>CH_4	286<br>136	Pt	Na_2S + Na_2SO_3	H_2O	*Angew. Chem. Int. Ed.* **2019,** *58*, 14549.
UiO-66/C_3N_4	300 W Xe lamp (> 400 nm)	CO	59.4	-	TEOA	CH_3CN	*Adv. Funct. Mater.* **2015,** *25*, 5360.
BIF-20@g-C_3N_4	300 W Xe lamp (> 400 nm)	CO<br>CH_4	53.869<br>15.524	-	TEOA	CH_3CN	*ACS Nano* **2018,** *12*, 5333.
Co tuned Au nanoclusters	300 W Xe lamp (≥ 420 nm)	CO	3.451	-	TEOA	H_2O	*J. Am. Chem. Soc.* **2018,** *140*, 16514.
Ni doped CdS quantum dots	300 W Xe lamp (> 400 nm)	CO	~9.5	-	TEOA	H_2O	*Angew. Chem. Int. Ed.* **2018,** *57*, 16447.
CdS	300 W Xe lamp (≥ 420 nm)	CO	34.51	dinuclear cobalt complex	TEOA	H_2O	*ACS Catal.* **2018,** *8*, 11815.
mpg-CN_x	Xe lamp (> 400 nm)	CO	18.99	$CoPPc_{12}$	TEOA	CH_3CN	*Angew. Chem. Int. Ed.* **2019,** *131*, 12308.

$CsPbBr_3$/GO	100 W Xe lamp (150 mW cm^{-2})	CO	23.7	-	-	Ethyl acetate	*J. Am. Chem. Soc.* **2017,** *139*, 5660.
$CsPbBr_3$/g-C_3N_4	300 W Xe lamp (≥ 420 nm)	CO	149	-	-	Acetonitrile/water and ethyl acetate/water	*Angew. Chem. Int. Ed.* **2018,** *130*, 13758.
$Cs_2AgBiBr_6$	AM 1.5G, 150 mW cm^{-2}	CO	105	-	-	Ethyl acetate	*Small* **2018,** *14*, 1703762.
$CsPbBr_3$/TiO_2	150 W Xe lamp (> 420 nm)	CO	11.71	-	Isopropanol	Ethyl acetate	*Adv. Mater. Interfaces* **2018,** *5*, 1801015.
Van Der Waals Gap-Rich BiOCl Atomic Layer	300W Xe (>400 nm)	CO	188.2	-	-	H_2O	This work

Supplementary Table 4. CO yield by CO_2 photoreduction using BOC-VDWGs-AL, as well as some comparisons to previous literatures.

Sample	Illumination condition	CO yield (μmol g^{-1} h^{-1})	Reference
Van Der Waals Gap-Rich BiOCl	AM 1.5 G	152.92	This work
$Cs_3Bi_2Br_9$	AM 1.5 G	26.95	*ACS Nano 2020, 14, 13103-13114.*
mpg-CN_x/CoPPc	AM 1.5 G	12.64	*Angew. Chem. 2019, 131, 12308-12312.*
MnO_x@TiO_2@CuPt	AM 1.5 G	84.2	*Chem. Sci., 2018, 9, 5334-5340.*
$Cs_2AgBiBr_6$	AM 1.5 G	105	*Small 2018, 14, 1703762.*
$CsPbBr_3$ QDs	AM 1.5 G	4.3	*Chem. Eur. J. 2017, 23, 9481-9485.*
$CsPbBr_3$ QDs/GO	AM 1.5 G	4.9	*J. Am. Chem. Soc. 2017, 139, 5660-5663.*
V_{Zn}-rich one-unit-cell $ZnIn_2S_4$ layers	AM 1.5 G	33.2	*J. Am. Chem. Soc., 2017, 139, 7586-7594.*
GO-O-rich TiO_2	AM 1.5 G	1.86	*Chem. Eng. J. 2017, 308, 248-255.*

Supplementary Table 5. Comparison of PCR stability tests in pure water with some representative photocatalysts.

Photocatalysts	Light source	Product	CO yield (μmol g^{-1} h^{-1})	Cycling test time (h)	Reaction medium	Reference
Van Der Waals Gap-Rich BiOCl Atomic Layer	>400 nm	CO	188.2	50	H_2O	This work
V_{Bi}-BiOBr NSs	UV-vis	CO	20.1	20	H_2O	*ACS Appl. Mater. Inter.* **2019**, *11*, 30876.
BiOCl@Bi_2O_3	>420 nm	CO	30	36	H_2O	*Adv. Mater.* **2020**, 2004311.
Br-grafted $Bi_2O_2(OH)(NO_3)$ NSs	UV-vis	CO	8.12	12	H_2O (vapor)	*Adv. Mater.* **2019**, 1900546.
$BiOIO_3$-OV	UV-vis	CO	17.33	12	H_2O (vapor)	*Adv. Mater.* **2020**, *32*, 1908350.
$Sr_2Bi_2Nb_2TiO_{12}$-OV	UV-vis	CO	17.11	4	H_2O (vapor)	*Angew. Chem. Int. Ed.* **2019**, *58*, 3880.
$Bi_4Ti_3O_{12}$-UOV	UV-vis	CO	11.7	4	H_2O (vapor)	*Sci. Bull.* **2020**, *65*, 934.
BiOBr-OVs Atomic Layers	>400 nm	CO	87.4	60	H_2O	*Angew. Chem. Int. Ed.* **2018**, *130*, 8855.
$Bi_{12}O_{17}Cl_2$ NTs	UV-vis	CO	48.6	12	H_2O	*Angew. Chem. Int. Ed.* **2018**, *130*, 1.
Partially oxidized SnS_2 atomic layers	>420 nm	CO	12.28	20	H_2O (vapor)	*J. Am. Chem. Soc.* **2017,** *139*, 18044.
BP@g-C_3N_4	UV-vis	CO	6.54	8	H_2O (vapor)	*Sci. China Mater.* **2018,** *61*, 1159.
SiC-NW/C	UV-vis	CO	5.87	9	H_2O (vapor)	*Adv. Mater.* **2020**, *32*, 2001560.
Ni-SA-5/ZrO_2	UV-vis	CO	11.8	25	H_2O (vapor)	*Adv. Energy Mater.* **2020**, 2002928.

Supplementary Methods

Computational Details. The theoretical calculations were performed using CASTEP package in which the plane-wave pseudopotential approach and ultrasoft pseudopotentials was employed for all the atoms with corresponding accuracy set as medium. The generalized gradient approximation (GGA) with the Perdew-Burkle-Ernzerhof (PBE) exchange-correlation function was employed. A (2×2) BiOCl (010) supercell with a slab thickness of four atoms (the Bi, O and Cl are roughly considered as locating in the same atomic layer) and a vacuum thickness being larger than 10 Å was used. All the models were first fully relaxed via geometry optimization and then applied for the energy calculation. All the transitional state (TS) search were conducted via a complete LST/QST protocol (the max number of QST step was set at 20) with 10 fragments from the initial state to the final state. The adsorption energies of adsorbates were defined as $E_{ad}(m) = E_{m\text{-}s} - E_s - E_m$, where m represents molecular adsorbate and s represents the surface of BiOCl (111).

Measurement of bulk charge separation efficiency (η_{bulk}). The indium doped tin oxide (ITO, China Southern Glass Co., Ltd., Shenzhen, China) substrates were first ultrasonically cleaned in distilled water, absolute ethanol, and isopropanol for 15 min sequentially. Both edges of the conducting glass substrates were then covered with adhesive tape. Typically, the aqueous slurries of the samples were spread on an ITO glass substrate with a glass rod, using adhesive tapes as spaces. The suspension was prepared by grinding 20 mg of samples, 40 μL of PEDOT-PSS (Sigma-Aldrich, 1.3-1.7%) aqueous solution, and 200 μL of water. The resulting film was dried in air and annealed at 150 °C for 10 min, yielding an electrode with catalyst loading amount of ca. 0.254 mg cm^{-2}. The photocurrents were measured by an electrochemical analyzer (CHI660D, Shanghai, China) in a standard three-electrode system with the samples as the working electrodes, a Pt foil as the counter electrode, and a saturated calomel electrode (SCE) as a reference electrode. A 300 W Xe arc lamp equipped with a 400 nm cutoff filter ($\lambda \geq 400$ nm) was utilized as a light source. We measured the η_{bulk} of BOC-VDWGs-AL, CBOC-VDWGs, and BOC-VDWGs-76 as follows. Briefly, the measured photocurrent densities (J) obey the following equation of $J = J_{abs} \times \eta_{bulk} \times \eta_{surface}$, where J_{abs} is the current

density converted from the absorbed photons when assuming that the absorbed photons were completely converted into electrons, η_{bulk} is the efficiency of e-h separation in the bulk of photocatalyst, and $\eta_{surface}$ is the efficiency of e-h separation on the surface of photocatalyst. When adding 1 M Na_2SO_3 as hole scavenger to the above system of photocurrent measurement, we assume that Na_2SO_3 can completely hinder the e-h recombination on the surface of photocatalyst without affecting e-h separation in the bulk of photocatalyst. In this case $\eta_{surface}$ is assumed to be 1, so η_{bulk} can be determined via $\eta_{bulk} = J_{sulfite}/J_{abs}$, where $J_{sulfite}$ is the photocurrent density in the presence of Na_2SO_3.
